# Supplementary material for: Comparative genomic analysis of innate immunity reveals novel and conserved components in crustacean food crop species
Source: BMC Genomics. 2017 May 18;18:389. doi: 10.1186/s12864-017-3769-4 (PMC5437397; doi:10.1186/s12864-017-3769-4)
Supplement: Supplementary file 1 — Phylogenetic relationship of Malacostraca. Malacostraca is shown within the Pancrustacea clade. Malacostraca tree is adapted from Melands and Willassen 2007. Decapod phylogeny is adapted from Scholtz and Richter 1995 and Schram [43]. Representative species are shown at each branch. Species denoted in purple are edible food crops. (PDF 153 kb) [file 12864_2017_3769_MOESM1_ESM.pdf]

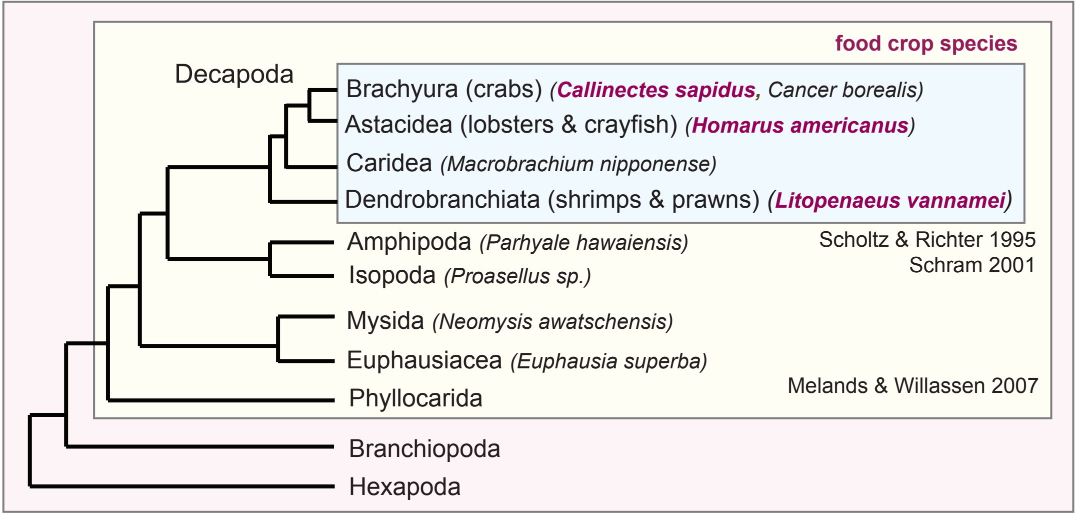
food crop species
Decapoda
Brachyura (crabs) (Callinectes sapidus, Cancer borealis)
Astacidea (lobsters & crayfish) (Homarus americanus)
Caridea (Macrobrachium nipponense)
Dendrobranchiata (shrimps & prawns) (Litopenaeus vannamei)
Amphipoda (Parhyale hawaiensis)
Isopoda (Proasellus sp.)
Scholtz & Richter 1995
Schram 2001
Mysida (Neomysis awatschensis)
Euphausiacea (Euphausia superba)
Phyllocarida
Melands & Willassen 2007
Branchiopoda
Hexapoda
