# Supplementary material for: Comparative genomic analysis of innate immunity reveals novel and conserved components in crustacean food crop species
Source: BMC Genomics. 2017 May 18;18:389. doi: 10.1186/s12864-017-3769-4 (PMC5437397; doi:10.1186/s12864-017-3769-4)
Supplement: Supplementary file 2 — A complete list of all 69 malacostracan transcriptome datasets used in this study along with information on tissue types, developmental stages, accession IDs and total number of transcripts for each transcriptome. (PDF 65 kb) [file 12864_2017_3769_MOESM2_ESM.pdf]

Additional file 1. A complete list of all 69 malacostracan transcriptome datasets used in this study.

Order	Species	Tissue type/conditions	Developmental stages	TSA sequence set	Accession	Number of sequences	References (for those available at the time of manuscript preparation)
Amphipoda	Parhyale hawaiensis	whole organism	NA	Kao et al., 2016	*P. hawaiensis transcriptome was generated from a combination of gene predictions from the genome assembly and RNAseq datasets.	28,666	http://dx.doi.org/10.7554/eLife.20062
Amphipoda	Echinogammarus veneris	NA	NA	GARO01000000	GARO01000001-GARO01031520	31,520	NA
Amphipoda	Gammarus chevreuxi	NA	embryo	HADC01000000	HADC01000001-HADC01040391	40,391	http://dx.doi.org/10.1016/j.margen.2016.02.002
Amphipoda	Gammarus pulex	Gammarus pulex males and females coming from uncontaminated river and contaminated river	NA	HAFM01000000	HAFM01000001-HAFM01010860	10,860	http://dx.doi.org/10.1016/j.gdata.2016.04.002
Amphipoda	Hyalella azteca_1	Exposed to pyrethroid insecticides	NA	GAJP01000000	GAJP01000001-GAJP01011022	11,022	doi: 10.1073/pnas.1302023110
Amphipoda	Hyalella azteca_2	Exposed to pyrethroid insecticides	NA	GAJQ01000000	GAJQ01000001-GAJQ01017788	17,788	doi: 10.1073/pnas.1302023110
Amphipoda	Hyalella azteca_3	whole organism	NA	GEHV01000000	GEHV01000001-GEHV01133409	133,409	http://dx.doi.org/10.1016/j.ygcen.2014.07.010
Amphipoda	Melita plumulosa	whole organism. RNA was harvested from control amphipods, juvenile amphipods, and from amphipods exposed to either metal or diesel contaminated sediments	NA	GAKD01000000	GAKD01000001-GAKD01025449	25,449	DOI:10.1016/j.aquatox.2013.11.022
Amphipoda	Talitrus saltator	brain	male and female	GDUJ01000000	GDUJ01000001-GDUJ01156706	156,706	DOI:10.7717/peerj.2555
Decapoda	Astacus astacus	hepatopancreas, ovaries, green glands, abdominal musculature	NA	GEDF01000000	GEDF01000001-GEDF01045338	45,338	http://dx.doi.org/10.1016/j.margen.2016.02.006
Decapoda	Astacus leptodactylus_1	hypodermis; Y organ. RNA samples were extracted from male Y organ and its neighboring hypodermis in all stages of the molt cycle	Adult	GAFS01000000	GAFS01000001-GAFS01074877	74,877	DOI: 10.1016/j.ygcen.2013.09.003
Decapoda	Astacus leptodactylus_2	hepatopancreas	Adult	GAFY01000000	GAFY01000001-GAFY01039935	39,935	DOI: 10.1371/journal.pone.0065176
Decapoda	Astacus leptodactylus_3	hypodermis, Y organ, hepatopancreas, gills, hematocytes, muscle	NA	GBEI01000000	GBEI01000001-GBEI01106362	106,362	DOI: 10.1371/journal.pone.0065176
Decapoda	Callinectes sapidus	gill 7. RNA-Seq in anterior (non-osmoregulatory) and posterior (osmoregulatory) gills during high to low salinity transfer in the blue crab	NA	GEID01000000	GEID01000001-GEID01229686	229,686	http://dx.doi.org/10.1016/j.cbd.2016.06.002
Decapoda	Cancer borealis	nervous system. equenced and performed de novo assembly for the nervous system transcriptomes of two decapod crustaceans: the Jonah crab (Cancer borealis) and the American lobster (Homarus americanus).	NA	GEFB01000000	GEFB01000001-GEFB01043216	43,216	DOI: 10.1186/s12864-016-3215-z
Decapoda	Carcinus maenas	Twelve sequence libraries corresponding to 12 pooled tissue samples from adult male and female C. maenas	NA	GBXE01000000	GBXE01000001-GBXE01212322	212,322	DOI: 10.1186/s12864-015-1667-1

Decapoda	Cherax quadricarinatus_1	hypodermis and gastrolith disc. Inter-molt crayfish were held in individual cages and endocrinologically induced to enter pre-molt through removal of the X organ–sinus gland (XO–SG) complex or, specifically for the 454-sequencing, through daily injection of 0.3 μg α-ecdysone per 1 g animal mass.	Adult	GADE01000000	GADE01000001-GADE01017518	17,518	DOI: 10.1242/jeb.080945
Decapoda	Cherax quadricarinatus_2	heart, kidney, liver, nerve, testis. Approximately 50 mg of heart, kidney, hepatopancreas, central nerve cord, and testis tissues were dissected from the euthanized crayfish	NA	HACB02000000	HACB02000001-HACB02105211	105,211	DOI: 10.1007/s13127-015-0237-3
Decapoda	Cherax quadricarinatus_3	heart, kidney, liver, nerve, testis. Approximately 50 mg of heart, kidney, hepatopancreas, central nerve cord, and testis tissues were dissected from the euthanized crayfish	NA	HACK01000000	HACK01000001-HACK01075424	75,424	DOI: 10.1007/s13127-015-0237-3
Decapoda	Eriocheir sinensis_1	RNA sequencing was applied to Z5 and M of E. sinensis	NA	GBUF01000000	GBUF01000001-GBUF01018686	18,686	DOI: 10.1016/j.cbd.2014.10.002
Decapoda	Eriocheir sinensis_2	eyestalk, Y-organ, and hepatopancreas. To obtain the complete transcriptome of the eyestalk, the whole eyestalk tissue was used for total RNA extraction.	NA	GBZW01000000	GBZW01000001-GBZW01041124	41,124	DOI: 10.1016/j.gene.2015.05.075
Decapoda	Eriocheir sinensis_3	hepatopancreas. The pooled total RNA from hepatopancreas of the precocious juvenile crabs, normal juvenile crabs and normal matured crabs was used to obtain a reference transcriptome.	NA	GEFT01000000	GEFT01000001-GEFT01040883	40,883	DOI: 10.1111/are.13189
Decapoda	Farfantepenaeus aztecus	hepatopancreas	NA	GEUA01000000	GEUA01000001-GEUA01127810	127,810	doi: 10.1093/icb/icw088
Decapoda	Homarus americanus	nervous system. Following anesthetization, the brain (supraoesophageal ganglion), ventral nerve cord, cardiac ganglion and complete stomatogastric nervous system (which includes the paired commissural ganglia and the single oesophageal and stomatogastric ganglia) were dissected out of each individual	NA	GEBG01000000	GEBG01000001-GEBG01067690	67,690	DOI: 10.1371/journal.pone.0145964

Decapoda	Hyas araneus_1	adult. Tissue samples comprising of all 6 gill-arches, tegument, heart, hepatopancreas, testis and pincer muscle were collected from four to six animals per treatment and directly frozen in liquid nitrogen.	NA	HAAI01000000	HAAI01000001-HAAI01019199	19,199	DOI: 10.1016/j.cbd.2013.09.004
Decapoda	Hyas araneus_2	gill	NA	HACH01000000	HACH01000001-HACH01064504	64,504	DOI: 10.1016/j.cbd.2013.09.004
Decapoda	Litopenaeus vannamei_1	abdominal muscle, hepatopancreas, gills and pleopods	NA	NA	NA	110,474	doi:10.1038/srep07081
Decapoda	Litopenaeus vannamei_2	hepatopancreas. Hepatopancreas mRNA of juvenile L. vannamei exposed to air-saturated water, low O2, or low O2/high CO2 for 4 or 24 h was pooled, sequenced (HiSeq 2500)	NA	GETD01000000	GETD01000001-GETD01052042	52,042	DOI: 10.1152/physiolgenomics.00031.2015
Decapoda	Litopenaeus vannamei_3	hepatopancreas		GETZ01000000	GETZ01000001-GETZ01101404	101,404	doi: 10.1093/icb/icw088
Decapoda	Litopenaeus vannamei_4. To better understand the virus-host interactions at the molecular level, the transcriptome profiles in hemocytes of unchallenged and WSSV-challenged shrimp (Litopenaeus vannamei) were compared using a short-read deep sequencing method (Illumina).	hemocytes	NA	HAAW01000000	HAAW01000001-HAAW01042151	42,151	http://dx.doi.org/10.1371/journal.pone.0076718
Decapoda	Macrobrachium nipponense	The control group was maintained under normoxic conditions (6.5 ± 0.2 mg O2/L). Hypoxic (2.5 ± 0.1 mg/L dissolved oxygen) conditions in the treatment tanks were maintained for 7 days by bubbling with N2 gas until the desired O2 concentrations were reached	NA	GCVG01000000	GCVG01000001-GCVG01062604	62,604	DOI: 10.1186/s12864-015-1701-3
Decapoda	Pacifastacus leniusculus	Brain, HPT, Hemocyte, Hepatopancreas	NA	GBYW01000000	GBYW01000001-GBYW01075939	75,939	NA
Decapoda	Palaemon argentinus	whole organism	NA	GEFN01000000	GEFN01000001-GEFN01024378	24,378	NA
Decapoda	Penaeus monodon_1	hepatopancreas	NA	GEEP01000000	GEEP01000001-GEEP01087484	87,484	NA
Decapoda	Penaeus monodon_2	hepatopancreas	NA	GEME01000000	GEME01000001-GEME01087496	87,496	NA
Decapoda	Procambarus clarkii_1	Eyestalk. Illumina RNA-sequencing was applied to characterize the eyestalk transcriptome and identify its most characterizing genes.	Adult	GARH01000000	GARH01000001-GARH01046670	46,670	DOI: 10.1016/j.gene.2014.12.001

Decapoda	Procambarus clarkii_2	Eyestalk, brain, hemocytes, gills, testis, ovary, hepatopancreas, heart, green gland, ventralganglia, Y-organ, hypodermis, muscle. The hypodermis underlying the anterior part of the carapace was sampled, taking care not to sample muscles adhered to the cuticle at that region, and immediately homogenized in RNA extraction solution	NA	GBEV01000000	GBEV01000001-GBEV01254561	254,561	DOI: 10.1242/jeb.109009
Decapoda	Scylla olivacea	Na	NA	GDRN01000000	GDRN01000001-GDRN01159835	159,835	NA
Decapoda	Scylla paramamosain	gill	NA	GEUT01000000	GEUT01000001-GEUT01013856	13,856	NA
Euphausiacea	Euphausia superba	NA	NA	HACF01000000	HACF01000001-HACF01058581	58,581	DOI: 10.1111/1755-0998.12408
Euphausiacea	Meganyctiphanes norvegica	adult	NA	GETT01000000	GETT01000001-GETT01405497	405,497	NA
Isopoda	Asellus aquaticus	Four samples were subjected to high-throughput sequencing: the head from a surface dwelling male, the head from a cave dwelling male, the head from a hybrid male (generated from a cave male and a surface female), and around thirty pooled surface individuals from 70% of embryonic development to hatching.	NA	GDKY01000000	GDKY01000001-GDKY01021684	21,684	http://dx.doi.org/10.1371/journal.pone.0140484
Isopoda	Bragasellus molinai	whole organism	NA	HAEM01000000	HAEM01000001-HAEM01209410	209,410	NA
Isopoda	Bragasellus peltatus	whole organism	NA	HAEN01000000	HAEN01000001-HAEN01107461	107,461	NA
Isopoda	Proasellus aragonensis	whole organism	NA	HAEO01000000	HAEO01000001-HAEO01094268	94,268	NA
Isopoda	Proasellus arthrodilus	whole organism	NA	HAEQ01000000	HAEQ01000001-HAEQ01125375	125,375	NA
Isopoda	Proasellus assaforensis	whole organism	NA	HAEP01000000	HAEP01000001-HAEP01092692	92,692	NA
Isopoda	Proasellus beticus	whole organism	NA	HAFL01000000	HAFL01000001-HAFL01228024	228,024	NA
Isopoda	Proasellus cantabricus	whole organism	NA	HAER01000000	HAER01000001-HAER01089661	89,661	NA
Isopoda	Proasellus cavaticus	whole organism	NA	HAES01000000	HAES01000001-HAES01150764	150,764	NA
Isopoda	Proasellus coiffaiti	whole organism	NA	HAEU01000000	HAEU01000001-HAEU01125332	125,332	NA
Isopoda	Proasellus coxalis	whole organism	NA	HAET01000000	HAET01000001-HAET01106655	106,655	NA
Isopoda	Proasellus ebrensis	whole organism	NA	HAEW01000000	HAEW01000001-HAEW01102589	102,589	NA
Isopoda	Proasellus escolai	whole organism	NA	HAEV01000000	HAEV01000001-HAEV01182024	182,024	NA
Isopoda	Proasellus grafi	whole organism	NA	HAEX01000000	HAEX01000001-HAEX01093309	93,309	NA
Isopoda	Proasellus granadensis	whole organism	NA	HAEY01000000	HAEY01000001-HAEY01147352	147,352	NA
Isopoda	Proasellus hercegovinensis	whole organism	NA	HAEZ01000000	HAEZ01000001-HAEZ01152457	152,457	NA
Isopoda	Proasellus ibericus	whole organism	NA	HAFA01000000	HAFA01000001-HAFA01150785	150,785	NA
Isopoda	Proasellus jaloniacus	whole organism	NA	HAFB01000000	HAFB01000001-HAFB01105212	105,212	NA
Isopoda	Proasellus karamani	whole organism	NA	HAFC01000000	HAFC01000001-HAFC01177517	177,517	NA
Isopoda	Proasellus margalefi	whole organism	NA	HAFD01000000	HAFD01000001-HAFD01098107	98,107	NA
Isopoda	Proasellus meridianus	whole organism	NA	HAFE01000000	HAFE01000001-HAFE01157215	157,215	NA
Isopoda	Proasellus ortizi	whole organism	NA	HAFF01000000	HAFF01000001-HAFF01088335	88,335	NA
Isopoda	Proasellus parvulus	whole organism	NA	HAFG01000000	HAFG01000001-HAFG01215610	215,610	NA
Isopoda	Proasellus racovitzai	whole organism	NA	HAFH01000000	HAFH01000001-HAFH01127707	127,707	NA
Isopoda	Proasellus rectus	whole organism	NA	HAFI01000000	HAFI01000001-HAFI01134529	134,529	NA
Isopoda	Proasellus solanasi	whole organism	NA	HAFJ01000000	HAFJ01000001-HAFJ01117044	117,044	NA

Isopoda	Proasellus spelaeus	whole organism	NA	HAFK01000000	HAFK01000001-HAFK01083849	83,849	NA
Mysida	Neomysis awatschensis	whole organism	NA	GDFV01000000	GDFV01000001-GDFV01022141	22,141	http://dx.doi.org/10.1016/j.margen.2016.05.001

7 Arthropoda proteomes

Class (subphylum)	Species	Tissue type	Accession	Number of sequences
Insecta	Drosophila melanogaster	whole organism	UP000000803	22,017
Insecta	Anopheles gambiae	whole organism	UP000007062	13,072
Insecta	Aedes aegypti	whole organism	UP000008820	16,654
Chilopoda (Myriapoda)	Strigamia maritima	whole organism	UP000014500	14,972
Arachnida (Chelicerata)	Mesobuthus martensii	whole organism	Cao et al., 2013	http://lifecenter.sgst.cn/main/en/scorpion.jsp
Arachnida (Chelicerata)	Ixodes scapularis	whole organism	UP000001555	20,473
Branchiopoda (Crustacea)	Daphnia pulex	whole organism	UP000000305	30,137
