# Supplementary material for: Comparative genomic analysis of innate immunity reveals novel and conserved components in crustacean food crop species
Source: BMC Genomics. 2017 May 18;18:389. doi: 10.1186/s12864-017-3769-4 (PMC5437397; doi:10.1186/s12864-017-3769-4)
Supplement: Supplementary file 3 — List of species used in graphs along with their corresponding number IDs. (PDF 46 kb) [file 12864_2017_3769_MOESM3_ESM.pdf]

Additional file 2. List of species used in graphs along with their corresponding number IDs.

Number ID	Species	Order
1	*Echinogammarus veneris*	Amphipoda
2	*Gammarus chevreuxi*	Amphipoda
3	*Gammarus pulex*	Amphipoda
4	*Hyalella azteca*	Amphipoda
5	*Melita plumulosa*	Amphipoda
6	*Parhyale hawaiensis*	Amphipoda
7	*Talitrus saltator*	Amphipoda
8	*Astacus astacus*	Decapoda
9	*Astacus leptodactylus*	Decapoda
10	*Callinectes sapidus*	Decapoda
11	*Cancer borealis*	Decapoda
12	*Carcinus maenas*	Decapoda
13	*Cherax quadricarinatus*	Decapoda
14	*Eriocheir sinensis*	Decapoda
15	*Farfantepenaeus aztecus*	Decapoda
16	*Homarus americanus*	Decapoda
17	*Hyas araneus*	Decapoda
18	*Litopenaeus vannamei*	Decapoda
19	*Macrobrachium nipponense*	Decapoda
20	*Pacifastacus leniusculus*	Decapoda
21	*Palaemon argentinus*	Decapoda
22	*Penaeus monodon*	Decapoda
23	*Procambarus clarkii*	Decapoda
24	*Scylla olivacea*	Decapoda
25	*Scylla paramamosain*	Decapoda
26	*Euphausia superba*	Euphausiacea
27	*Meganyctiphanes norvegica*	Euphausiacea
28	*Asellus aquaticus*	Isopoda
29	*Bragasellus molinai*	Isopoda
30	*Bragasellus peltatus*	Isopoda
31	*Proasellus aragonensis*	Isopoda
32	*Proasellus arthrodilus*	Isopoda
33	*Proasellus assaforensis*	Isopoda
34	*Proasellus beticus*	Isopoda
35	*Proasellus cantabricus*	Isopoda
36	*Proasellus cavaticus*	Isopoda
37	*Proasellus coiffaiti*	Isopoda
38	*Proasellus coxalis*	Isopoda
39	*Proasellus ebrensis*	Isopoda
40	*Proasellus escolai*	Isopoda
41	*Proasellus grafi*	Isopoda
42	*Proasellus granadensis*	Isopoda

43	*Proasellus hercegovinensis*	Isopoda
44	*Proasellus ibericus*	Isopoda
45	*Proasellus jaloniacus*	Isopoda
46	*Proasellus karamani*	Isopoda
47	*Proasellus margalefi*	Isopoda
48	*Proasellus meridianus*	Isopoda
49	*Proasellus ortizi*	Isopoda
50	*Proasellus parvulus*	Isopoda
51	*Proasellus racovitzai*	Isopoda
52	*Proasellus rectus*	Isopoda
53	*Proasellus solanasi*	Isopoda
54	*Proasellus spelaeus*	Isopoda
55	*Neomysis awatschensis*	Mysida
