# Supplementary material for: Comparative genomic analysis of innate immunity reveals novel and conserved components in crustacean food crop species
Source: BMC Genomics. 2017 May 18;18:389. doi: 10.1186/s12864-017-3769-4 (PMC5437397; doi:10.1186/s12864-017-3769-4)
Supplement: Supplementary file 4 — Malacostracans pattern recognition receptors and proPOs. (PDF 639 kb) [file 12864_2017_3769_MOESM4_ESM.pdf]

Additional file 3. Malacostracans pattern recognition receptors and prophenoloxidases.

Additional file 3A. Gram negative binding proteins.

Arthropoda

Class (subphylum)	Species	Tissue type	Total gene counts	References
Insecta	Drosophila melanogaster	whole organism	3	Palmer et al., 2015
Insecta	Anopheles gambiae	whole organism	7	McTaggart et al., 2009
Insecta	Aedes aegypti	whole organism	7	McTaggart et al., 2009
Chilopoda (Myriapoda)	Strigamia maritima	whole organism	1	Palmer et al., 2015
Arachnida (Chelicerata)	Mesobuthus martensii	whole organism	0	Palmer et al., 2015
Arachnida (Chelicerata)	Ixodes scapularis	whole organism	0	Palmer et al., 2015
Branchiopoda (Crustacea)	Daphnia pulex	whole organism	11	McTaggart et al., 2009

Malacostraca

Order	Species/Datasets	Tissue type	Total gene counts	Total number of non-redundant genes per species
Amphipoda	Echinogammarus veneris	NA	5	5
Amphipoda	Gammarus chevreuxi	NA	3	3
Amphipoda	Gammarus pulex	NA	7	7
Amphipoda	Hyalella azteca_1	NA	3	
Amphipoda	Hyalella azteca_2	NA	4	
Amphipoda	Hyalella azteca_3	whole organism	1	3
Amphipoda	Melita plumulosa	whole organism	2	2
Amphipoda	Parhyale hawaiensis	whole organism	3	3
Amphipoda	Talitrus saltator	brain	2	2
Decapoda	Astacus astacus	hepatopancreas, ovaries, green glands, abdominal musculature	2	2
Decapoda	Astacus leptodactylus_1	hypodermis; Y organ	5	
Decapoda	Astacus leptodactylus_2	hepatopancreas	4	
Decapoda	Astacus leptodactylus_3	hypodermis, Y organ, hepatopancreas, gills, hematocytes, muscle	2	7
Decapoda	Callinectes sapidus	gill 7	0	0
Decapoda	Cancer borealis	nervous system	3	3
Decapoda	Carcinus maenas	NA	1	1
Decapoda	Cherax quadricarinatus_1	hypodermis and gastrolith disc	2	
Decapoda	Cherax quadricarinatus_2	heart, kidney, liver, nerve, testis	2	
Decapoda	Cherax quadricarinatus_3	heart, kidney, liver, nerve, testis	2	2
Decapoda	Eriocheir sinensis_1	NA	3	
Decapoda	Eriocheir sinensis_2	eyestalk, Y-organ, and hepatopancreas	3	
Decapoda	Eriocheir sinensis_3	hepatopancreas	4	7
Decapoda	Farfantepenaeus aztecus	hepatopancreas	4	4
Decapoda	Homarus americanus	nervous system	2	2
Decapoda	Hyas araneus_1	adult	1	
Decapoda	Hyas araneus_2	gill	1	1
Decapoda	Litopenaeus vannamei_1	Ghaffari et al., 2014	5	
Decapoda	Litopenaeus vannamei_2	hepatopancreas	7	
Decapoda	Litopenaeus vannamei_3	hepatopancreas	6	
Decapoda	Litopenaeus vannamei_4	hemocytes	2	5
Decapoda	Macrobrachium nipponense	NA	1	1
Decapoda	Pacifastacus leniusculus	Brain, HPT, Hemocyte, Hepatopancreas	5	5
Decapoda	Palaemon argentinus	whole organism	4	4
Decapoda	Penaeus monodon_1	hepatopancreas	4	
Decapoda	Penaeus monodon_2	hepatopancreas	4	4
Decapoda	Procambarus clarkii_1	Eyestalk	3	
Decapoda	Procambarus clarkii_2	Eyestalk, brain, hemocytes, gills, testis, ovary, hepatopancreas, heart, green gland, ventralganglia, Y-organ, hypodermis, muscle	6	8
Decapoda	Scylla olivacea	Na	1	1
Decapoda	Scylla paramamosain	gill	0	0
Euphausiacea	Euphausia superba	NA	4	4
Euphausiacea	Meganyctiphanes norvegica	adult	0	0
Isopoda	Asellus aquaticus	NA	2	2
Isopoda	Bragasellus molinai	whole organism	2	2
Isopoda	Bragasellus peltatus	whole organism	2	2
Isopoda	Proasellus aragonensis	whole organism	4	4
Isopoda	Proasellus arthrodilus	whole organism	5	5
Isopoda	Proasellus assaforensis	whole organism	4	4
Isopoda	Proasellus beticus	whole organism	3	3
Isopoda	Proasellus cantabricus	whole organism	2	2
Isopoda	Proasellus cavaticus	whole organism	5	5

Isopoda	Proasellus coiffaiti	whole organism	10	10	
Isopoda	Proasellus coxalis	whole organism	8	8	
Isopoda	Proasellus ebrensis	whole organism	2	2	
Isopoda	Proasellus escolai	whole organism	3	3	
Isopoda	Proasellus grafi	whole organism	3	3	
Isopoda	Proasellus granadensis	whole organism	2	2	
Isopoda	Proasellus hercegovinensis	whole organism	3	3	
Isopoda	Proasellus ibericus	whole organism	6	6	
Isopoda	Proasellus jaloniacus	whole organism	4	4	
Isopoda	Proasellus karamani	whole organism	10	10	
Isopoda	Proasellus margalefi	whole organism	4	4	
Isopoda	Proasellus meridianus	whole organism	5	5	
Isopoda	Proasellus ortizi	whole organism	3	3	
Isopoda	Proasellus parvulus	whole organism	6	6	
Isopoda	Proasellus racovitzai	whole organism	4	4	
Isopoda	Proasellus rectus	whole organism	5	5	
Isopoda	Proasellus solanasi	whole organism	4	4	
Isopoda	Proasellus spelaeus	whole organism	2	2	
Mysida	Neomysis awatschensis	whole organism	3	3	
			**Total malacostracan genes**		202

Additional file 3B. Down Syndrome Cell Adhesion Molecule.

Arthropoda

Class (subphylum)	Species	Tissue type	Total gene counts	References
Insecta	Drosophila melanogaster	whole organism	1	McTaggart et al., 2009
Insecta	Anopheles gambiae	whole organism	1	McTaggart et al., 2009
Insecta	Aedes aegypti	whole organism	1	McTaggart et al., 2009
Chilopoda (Myriapoda)	Strigamia maritima	whole organism	60	Palmer et al., 2015
Arachnida (Chelicerata)	Mesobuthus martensii	whole organism	12	Palmer et al., 2015
Arachnida (Chelicerata)	Ixodes scapularis	whole organism	13	Palmer et al., 2015
Branchiopoda (Crustacea)	Daphnia pulex	whole organism	1	McTaggart et al., 2009

Malacostraca

Order	Species/Datasets	Tissue type	Total gene counts	Total number of non-redundant genes per species
Amphipoda	Echinogammarus veneris	NA	0	0
Amphipoda	Gammarus chevreuxi	embryo	1	1
Amphipoda	Gammarus pulex	NA	0	0
Amphipoda	Hyalella azteca_1	NA	0	
Amphipoda	Hyalella azteca_2	NA	0	
Amphipoda	Hyalella azteca_3	whole organism	multiple isoforms	1
Amphipoda	Melita plumulosa	whole organism	1	1
Amphipoda	Parhyale hawaiensis	whole organism	multiple isoforms	1
Amphipoda	Talitrus saltator	brain	multiple isoforms	1
Decapoda	Astacus astacus	hepatopancreas, ovaries, green glands, abdominal musculature	1	
Decapoda	Astacus leptodactylus_1	hypodermis; Y organ	1	
Decapoda	Astacus leptodactylus_2	hepatopancreas	1	1
Decapoda	Astacus leptodactylus_3	hypodermis, Y organ, hepatopancreas, gills, hematocytes, muscle	1	1
Decapoda	Callinectes sapidus	gill 7	1	1
Decapoda	Cancer borealis	nervous system	multiple isoforms	1
Decapoda	Carcinus maenas	NA	1	1
Decapoda	Cherax quadricarinatus_1	hypodermis and gastrolith disc	0	
Decapoda	Cherax quadricarinatus_2	heart, kidney, liver, nerve, testis	multiple isoforms	
Decapoda	Cherax quadricarinatus_3	heart, kidney, liver, nerve, testis	multiple isoforms	1
Decapoda	Eriocheir sinensis_1	NA	1	
Decapoda	Eriocheir sinensis_2	eyestalk, Y-organ, and hepatopancreas	0	
Decapoda	Eriocheir sinensis_3	hepatopancreas	1	1
Decapoda	Farfantepenaeus aztecus	hepatopancreas	1	1
Decapoda	Homarus americanus	nervous system	1	1
Decapoda	Hyas araneus_1	adult	1	
Decapoda	Hyas araneus_2	gill	multiple isoforms	1
Decapoda	Litopenaeus vannamei_1	Ghaffari et al., 2014	multiple isoforms	
Decapoda	Litopenaeus vannamei_2	hepatopancreas	multiple isoforms	
Decapoda	Litopenaeus vannamei_3	hepatopancreas	1	
Decapoda	Litopenaeus vannamei_4	hemocytes	0	1
Decapoda	Macrobrachium nipponense	NA	1	1
Decapoda	Pacifastacus leniusculus	Brain, HPT, Hemocyte, Hepatopancreas	multiple isoforms	1
Decapoda	Palaemon argentinus	whole organism	0	0
Decapoda	Penaeus monodon_1	hepatopancreas	0	
Decapoda	Penaeus monodon_2	hepatopancreas	0	0
Decapoda	Procambarus clarkii_1	Eyestalk	multiple isoforms	
Decapoda	Procambarus clarkii_2	Eyestalk, brain, hemocytes, gills, testis, ovary, hepatopancreas, heart, green gland, ventralganglia, Y-organ, hypodermis, muscle	multiple isoforms	1
Decapoda	Scylla olivacea	Na	multiple isoforms	1
Decapoda	Scylla paramamosain	gill	uniprot, not in gills	1
Euphausiacea	Euphausia superba	NA	0	0
Euphausiacea	Meganyctiphanes norvegica	adult	1	1
Isopoda	Asellus aquaticus	NA	0	0
Isopoda	Bragasellus molinai	whole organism	1	1
Isopoda	Bragasellus peltatus	whole organism	1	1
Isopoda	Proasellus aragonensis	whole organism	1	1
Isopoda	Proasellus arthrodilus	whole organism	1	1
Isopoda	Proasellus assaforensis	whole organism	1	1

Isopoda	Proasellus beticus	whole organism	multiple isoforms	1
Isopoda	Proasellus cantabricus	whole organism	multiple isoforms	1
Isopoda	Proasellus cavaticus	whole organism	1	1
Isopoda	Proasellus coiffaiti	whole organism	1	1
Isopoda	Proasellus coxalis	whole organism	1	1
Isopoda	Proasellus ebrensis	whole organism	multiple isoforms	1
Isopoda	Proasellus escolai	whole organism	1	1
Isopoda	Proasellus grafi	whole organism	1	1
Isopoda	Proasellus granadensis	whole organism	multiple isoforms	1
Isopoda	Proasellus hercegovinensis	whole organism	multiple isoforms	1
Isopoda	Proasellus ibericus	whole organism	multiple isoforms	1
Isopoda	Proasellus jaloniacus	whole organism	1	1
Isopoda	Proasellus karamani	whole organism	multiple isoforms	1
Isopoda	Proasellus margalefi	whole organism	1	1
Isopoda	Proasellus meridianus	whole organism	multiple isoforms	1
Isopoda	Proasellus ortizi	whole organism	1	1
Isopoda	Proasellus parvulus	whole organism	multiple isoforms	1
Isopoda	Proasellus racovitzai	whole organism	multiple isoforms	1
Isopoda	Proasellus rectus	whole organism	multiple isoforms	1
Isopoda	Proasellus solanasi	whole organism	multiple isoforms	1
Isopoda	Proasellus spelaeus	whole organism	multiple isoforms	1
Mysida	Neomysis awatschensis	whole organism	multiple isoforms	1
			**Total malacostracan genes**	49

Additional file 3C. Domeless.

Arthropoda

Class (subphylum)	Species	Tissue type	Total gene counts	References
Insecta	Drosophila melanogaster	whole organism	1	Palmer et al., 2015
Insecta	Anopheles gambiae	whole organism	1	ImmunoDB
Insecta	Aedes aegypti	whole organism	1	ImmunoDB
Chilopoda (Myriapoda)	Strigamia maritima	whole organism	2	Palmer et al., 2015
Arachnida (Chelicerata)	Mesobuthus martensii	whole organism	0	Palmer et al., 2015
Arachnida (Chelicerata)	Ixodes scapularis	whole organism	2	Palmer et al., 2015
Branchiopoda (Crustacea)	Daphnia pulex	whole organism	1	Palmer et al., 2015

Malacostraca

Order	Species/Datasets	Tissue type	Total gene counts	Total number of non-redundant genes per species
Amphipoda	Echinogammarus veneris	NA	0	0
Amphipoda	Gammarus chevreuxi	NA	0	0
Amphipoda	Gammarus pulex	NA	0	0
Amphipoda	Hyalella azteca_1	NA	0	
Amphipoda	Hyalella azteca_2	NA	0	
Amphipoda	Hyalella azteca_3	whole organism	1	1
Amphipoda	Melita plumulosa	whole organism	1	1
Amphipoda	Parhyale hawaiensis	whole organism	1	1
Amphipoda	Talitrus saltator	brain	1	1
Decapoda	Astacus astacus	hepatopancreas, ovaries, green glands, abdominal musculature	1	1
Decapoda	Astacus leptodactylus_1	hypodermis; Y organ	1	
Decapoda	Astacus leptodactylus_2	hepatopancreas	0	
Decapoda	Astacus leptodactylus_3	hypodermis, Y organ, hepatopancreas, gills, hematocytes, muscle	1	1
Decapoda	Callinectes sapidus	gill 7	0	0
Decapoda	Cancer borealis	nervous system	1	1
Decapoda	Carcinus maenas	NA	1	1
Decapoda	Cherax quadricarinatus_1	hypodermis and gastrolith disc	0	
Decapoda	Cherax quadricarinatus_2	heart, kidney, liver, nerve, testis	1	
Decapoda	Cherax quadricarinatus_3	heart, kidney, liver, nerve, testis	1	1
Decapoda	Eriocheir sinensis_1	NA	1	
Decapoda	Eriocheir sinensis_2	eyestalk, Y-organ, and hepatopancreas	1	
Decapoda	Eriocheir sinensis_3	hepatopancreas	1	1
Decapoda	Farfantepenaeus aztecus	hepatopancreas	1	1
Decapoda	Homarus americanus	nervous system	1	1
Decapoda	Hyas araneus_1	adult	1	
Decapoda	Hyas araneus_2	gill	1	1
Decapoda	Litopenaeus vannamei_1	Ghaffari et al., 2014	1	
Decapoda	Litopenaeus vannamei_2	hepatopancreas	1	
Decapoda	Litopenaeus vannamei_3	hepatopancreas	1	
Decapoda	Litopenaeus vannamei_4	hemocytes	1	1
Decapoda	Macrobrachium nipponense	NA	1	1
Decapoda	Pacifastacus leniusculus	Brain, HPT, Hemocyte, Hepatopancreas	1	1
Decapoda	Palaemon argentinus	whole organism	0	0
Decapoda	Penaeus monodon_1	hepatopancreas	1	
Decapoda	Penaeus monodon_2	hepatopancreas	1	1
Decapoda	Procambarus clarkii_1	Eyestalk	1	
Decapoda	Procambarus clarkii_2	Eyestalk, brain, hemocytes, gills, testis, ovary, hepatopancreas, heart, green gland, ventralganglia, Y-organ, hypodermis, muscle	1	1
Decapoda	Scylla olivacea	Na	1	1
Decapoda	Scylla paramamosain	gill	0	0
Euphausiacea	Euphausia superba	NA	1	1
Euphausiacea	Meganyctiphanes norvegica	adult	1	1
Isopoda	Asellus aquaticus	NA	0	0
Isopoda	Bragasellus molinai	whole organism	1	1
Isopoda	Bragasellus peltatus	whole organism	1	1
Isopoda	Proasellus aragonensis	whole organism	1	1
Isopoda	Proasellus arthrodilus	whole organism	1	1
Isopoda	Proasellus assaforensis	whole organism	1	1
Isopoda	Proasellus beticus	whole organism	1	1

Isopoda	Proasellus cantabricus	whole organism	1	1	
Isopoda	Proasellus cavaticus	whole organism	1	1	
Isopoda	Proasellus coiffaiti	whole organism	1	1	
Isopoda	Proasellus coxalis	whole organism	1	1	
Isopoda	Proasellus ebrensis	whole organism	1	1	
Isopoda	Proasellus escolai	whole organism	1	1	
Isopoda	Proasellus grafi	whole organism	1	1	
Isopoda	Proasellus granadensis	whole organism	1	1	
Isopoda	Proasellus hercegovinensis	whole organism	1	1	
Isopoda	Proasellus ibericus	whole organism	1	1	
Isopoda	Proasellus jaloniacus	whole organism	1	1	
Isopoda	Proasellus karamani	whole organism	1	1	
Isopoda	Proasellus margalefi	whole organism	1	1	
Isopoda	Proasellus meridianus	whole organism	1	1	
Isopoda	Proasellus ortizi	whole organism	1	1	
Isopoda	Proasellus parvulus	whole organism	1	1	
Isopoda	Proasellus racovitzai	whole organism	1	1	
Isopoda	Proasellus rectus	whole organism	1	1	
Isopoda	Proasellus solanasi	whole organism	1	1	
Isopoda	Proasellus spelaeus	whole organism	1	1	
Mysida	Neomysis awatschensis	whole organism	0	0	
			**Total malacostracan genes**		47

Additional file 3D. Galectin.

Arthropoda

Class (subphylum)	Species	Tissue type	Total gene counts	Reference
Insecta	Drosophila melanogaster	whole organism	7	ImmunoDB
Insecta	Anopheles gambiae	whole organism	11	ImmunoDB
Insecta	Aedes aegypti	whole organism	12	ImmunoDB
Chilopoda (Myriapoda)	Strigamia maritima	whole organism	2	Kao et al., 2016
Arachnida (Chelicerata)	Mesobuthus martensii	whole organism	6	Kao et al., 2016
Arachnida (Chelicerata)	Ixodes scapularis	whole organism	5	Kao et al., 2016
Branchiopoda (Crustacea)	Daphnia pulex	whole organism	3	McTaggart et al., 2009

Malacostraca

Order	Species/Datasets	Tissue type	Total gene counts	Total number of non-redundant genes per species
Amphipoda	Echinogammarus veneris	NA	1	1
Amphipoda	Gammarus chevreuxi	NA	1	1
Amphipoda	Gammarus pulex	NA	0	0
Amphipoda	Hyalella azteca_1	NA	0	
Amphipoda	Hyalella azteca_2	NA	0	
Amphipoda	Hyalella azteca_3	whole organism	1	1
Amphipoda	Melita plumulosa	whole organism	1	1
Amphipoda	Parhyale hawaiensis	whole organism	1	1
Amphipoda	Talitrus saltator	brain	1	1
Decapoda	Astacus astacus	hepatopancreas, ovaries, green glands, abdominal musculature	1	1
Decapoda	Astacus leptodactylus_1	hypodermis; Y organ	1	
Decapoda	Astacus leptodactylus_2	hepatopancreas	0	
Decapoda	Astacus leptodactylus_3	hypodermis, Y organ, hepatopancreas, gills, hematocytes, muscle	1	1
Decapoda	Callinectes sapidus	gill 7	1	1
Decapoda	Cancer borealis	nervous system	1	1
Decapoda	Carcinus maenas	NA	1	1
Decapoda	Cherax quadricarinatus_1	hypodermis and gastrolith disc	0	
Decapoda	Cherax quadricarinatus_2	heart, kidney, liver, nerve, testis	1	
Decapoda	Cherax quadricarinatus_3	heart, kidney, liver, nerve, testis	1	1
Decapoda	Eriocheir sinensis_1	NA	1	
Decapoda	Eriocheir sinensis_2	eyestalk, Y-organ, and hepatopancreas	1	
Decapoda	Eriocheir sinensis_3	hepatopancreas	1	1
Decapoda	Farfantepenaeus aztecus	hepatopancreas	1	1
Decapoda	Homarus americanus	nervous system	1	1
Decapoda	Hyas araneus_1	adult	1	
Decapoda	Hyas araneus_2	gill	1	1
Decapoda	Litopenaeus vannamei_1	Ghaffari et al., 2014	1	
Decapoda	Litopenaeus vannamei_2	hepatopancreas	1	
Decapoda	Litopenaeus vannamei_3	hepatopancreas	1	
Decapoda	Litopenaeus vannamei_4	hemocytes	1	1
Decapoda	Macrobrachium nipponense	NA	1	1
Decapoda	Pacifastacus leniusculus	Brain, HPT, Hemocyte, Hepatopancreas	0	0
Decapoda	Palaemon argentinus	whole organism	0	0
Decapoda	Penaeus monodon_1	hepatopancreas	1	
Decapoda	Penaeus monodon_2	hepatopancreas	1	1
Decapoda	Procambarus clarkii_1	Eyestalk	1	
Decapoda	Procambarus clarkii_2	Eyestalk, brain, hemocytes, gills, testis, ovary, hepatopancreas, heart, green gland, ventralganglia, Y-organ, hypodermis, muscle	1	1
Decapoda	Scylla olivacea	Na	0	0
Decapoda	Scylla paramamosain	gill	0	0
Euphausiacea	Euphausia superba	NA	1	1
Euphausiacea	Meganyctiphanes norvegica	adult	0	0
Isopoda	Asellus aquaticus	NA	2	2
Isopoda	Bragasellus molinai	whole organism	1	1
Isopoda	Bragasellus peltatus	whole organism	2	2
Isopoda	Proasellus aragonensis	whole organism	1	1
Isopoda	Proasellus arthrodilus	whole organism	1	1
Isopoda	Proasellus assaforensis	whole organism	1	1
Isopoda	Proasellus beticus	whole organism	0	0
Isopoda	Proasellus cantabricus	whole organism	1	1
Isopoda	Proasellus cavaticus	whole organism	1	1
Isopoda	Proasellus coiffaiti	whole organism	1	1
Isopoda	Proasellus coxalis	whole organism	1	1

Isopoda	Proasellus ebrensis	whole organism	1	1
Isopoda	Proasellus escolai	whole organism	1	1
Isopoda	Proasellus grafi	whole organism	1	1
Isopoda	Proasellus granadensis	whole organism	1	1
Isopoda	Proasellus hercegovinensis	whole organism	1	1
Isopoda	Proasellus ibericus	whole organism	1	1
Isopoda	Proasellus jaloniacus	whole organism	1	1
Isopoda	Proasellus karamani	whole organism	1	1
Isopoda	Proasellus margalefi	whole organism	0	0
Isopoda	Proasellus meridianus	whole organism	1	1
Isopoda	Proasellus ortizi	whole organism	1	1
Isopoda	Proasellus parvulus	whole organism	0	0
Isopoda	Proasellus racovitzai	whole organism	1	1
Isopoda	Proasellus rectus	whole organism	1	1
Isopoda	Proasellus solanasi	whole organism	1	1
Isopoda	Proasellus spelaeus	whole organism	1	1
Mysida	Neomysis awatschensis	whole organism	0	0
			**Total malacostracan genes**	47

Additional file 3E. Thioester containing protein, alpha-2 macroglobulin, macroglobulin complement related proteins.

Arthropoda

Class (subphylum)	Species	Tissue type	Total gene counts	References
Insecta	Drosophila melanogaster	whole organism	6	Palmer et al., 2015
Insecta	Anopheles gambiae	whole organism	13	ImmunoDB
Insecta	Aedes aegypti	whole organism	8	ImmunoDB
Chilopoda (Myriapoda)	Strigamia maritima	whole organism	4	Palmer et al., 2015
Arachnida (Chelicerata)	Mesobuthus martensii	whole organism	1	Palmer et al., 2015
Arachnida (Chelicerata)	Ixodes scapularis	whole organism	3	Palmer et al., 2015
Branchiopoda (Crustacea)	Daphnia pulex	whole organism	7	Palmer et al., 2015

Malacostraca

Order	Species/Datasets	Tissue type	Total gene counts	Total number of non-redundant genes per species
Amphipoda	Echinogammarus veneris	NA	4	4
Amphipoda	Gammarus chevreuxi	NA	3	3
Amphipoda	Gammarus pulex	NA	2	2
Amphipoda	Hyalella azteca_1	NA	3	
Amphipoda	Hyalella azteca_2	NA	2	
Amphipoda	Hyalella azteca_3	whole organism	5	5
Amphipoda	Melita plumulosa	whole organism	4	4
Amphipoda	Parhyale hawaiensis	whole organism	4	4
Amphipoda	Talitrus saltator	brain	8	8
Decapoda	Astacus astacus	hepatopancreas, ovaries, green glands, abdominal musculature	3	3
Decapoda	Astacus leptodactylus_1	hypodermis; Y organ	12	
Decapoda	Astacus leptodactylus_2	hepatopancreas	4	
Decapoda	Astacus leptodactylus_3	hypodermis, Y organ, hepatopancreas, gills, hematocytes, muscle	18	22
Decapoda	Callinectes sapidus	gill 7	4	4
Decapoda	Cancer borealis	nervous system	11	11
Decapoda	Carcinus maenas	NA	6	6
Decapoda	Cherax quadricarinatus_1	hypodermis and gastrolith disc	9	
Decapoda	Cherax quadricarinatus_2	heart, kidney, liver, nerve, testis	8	
Decapoda	Cherax quadricarinatus_3	heart, kidney, liver, nerve, testis	8	8
Decapoda	Eriocheir sinensis_1	NA	11	
Decapoda	Eriocheir sinensis_2	eyestalk, Y-organ, and hepatopancreas	10	
Decapoda	Eriocheir sinensis_3	hepatopancreas	9	12
Decapoda	Farfantepenaeus aztecus	hepatopancreas	9	9
Decapoda	Homarus americanus	nervous system	17	17
Decapoda	Hyas araneus_1	adult	10	
Decapoda	Hyas araneus_2	gill	14	11
Decapoda	Litopenaeus vannamei_1	Ghaffari et al., 2014	12	
Decapoda	Litopenaeus vannamei_2	hepatopancreas	16	
Decapoda	Litopenaeus vannamei_3	hepatopancreas	9	
Decapoda	Litopenaeus vannamei_4	hemocytes	14	17
Decapoda	Macrobrachium nipponense	NA	11	11
Decapoda	Pacifastacus leniusculus	Brain, HPT, Hemocyte, Hepatopancreas	8	8
Decapoda	Palaemon argentinus	whole organism	9	9
Decapoda	Penaeus monodon_1	hepatopancreas	3	
Decapoda	Penaeus monodon_2	hepatopancreas	3	3
Decapoda	Procambarus clarkii_1	Eyestalk	18	
Decapoda	Procambarus clarkii_2	Eyestalk, brain, hemocytes, gills, testis, ovary, hepatopancreas, heart, green gland, ventralganglia, Y-organ, hypodermis, muscle	20	25
Decapoda	Scylla olivacea	Na	8	8
Decapoda	Scylla paramamosain	gill	10	10
Euphausiacea	Euphausia superba	NA	8	8
Euphausiacea	Meganyctiphanes norvegica	adult	4	4
Isopoda	Asellus aquaticus	NA	7	7
Isopoda	Bragasellus molinai	whole organism	9	9
Isopoda	Bragasellus peltatus	whole organism	7	7
Isopoda	Proasellus aragonensis	whole organism	6	6
Isopoda	Proasellus arthrodilus	whole organism	8	8
Isopoda	Proasellus assaforensis	whole organism	10	10
Isopoda	Proasellus beticus	whole organism	3	3

Isopoda	Proasellus cantabricus	whole organism	7	7
Isopoda	Proasellus cavaticus	whole organism	9	9
Isopoda	Proasellus coiffaiti	whole organism	9	9
Isopoda	Proasellus coxalis	whole organism	7	7
Isopoda	Proasellus ebrensis	whole organism	9	9
Isopoda	Proasellus escolai	whole organism	8	8
Isopoda	Proasellus grafi	whole organism	8	8
Isopoda	Proasellus granadensis	whole organism	9	9
Isopoda	Proasellus hercegovinensis	whole organism	9	9
Isopoda	Proasellus ibericus	whole organism	8	8
Isopoda	Proasellus jaloniacus	whole organism	4	4
Isopoda	Proasellus karamani	whole organism	6	6
Isopoda	Proasellus margalefi	whole organism	4	4
Isopoda	Proasellus meridianus	whole organism	8	8
Isopoda	Proasellus ortizi	whole organism	5	5
Isopoda	Proasellus parvulus	whole organism	5	5
Isopoda	Proasellus racovitzai	whole organism	5	5
Isopoda	Proasellus rectus	whole organism	7	7
Isopoda	Proasellus solanasi	whole organism	5	5
Isopoda	Proasellus spelaeus	whole organism	7	7
Mysida	Neomysis awatschensis	whole organism	7	7
			**Total malacostracan genes**	432

Additional file 3F. C-type lectins.

Arthropoda

Class (subphylum)	Species	Tissue type	Total gene counts	References
Insecta	Drosophila melanogaster	whole organism	33	ImmunoDB
Insecta	Anopheles gambiae	whole organism	25	ImmunoDB
Insecta	Aedes aegypti	whole organism	40	ImmunoDB
Chilopoda (Myriapoda)	Strigamia maritima	whole organism	25	Kao et al., 2016
Arachnida (Chelicerata)	Mesobuthus martensii	whole organism	24	Kao et al., 2016
Arachnida (Chelicerata)	Ixodes scapularis	whole organism	17	Kao et al., 2016
Branchiopoda (Crustacea)	Daphnia pulex	whole organism	26	Kao et al., 2016

Malacostraca

Order	Species/Datasets	Tissue type	Total gene counts	Total number of non-redundant genes per species
Amphipoda	Echinogammarus veneris	NA	5	5
Amphipoda	Gammarus chevreuxi	NA	5	5
Amphipoda	Gammarus pulex	NA	49	49
Amphipoda	Hyalella azteca_1	NA	4	
Amphipoda	Hyalella azteca_2	NA	6	
Amphipoda	Hyalella azteca_3	whole organism	11	18
Amphipoda	Melita plumulosa	whole organism	4	4
Amphipoda	Parhyale hawaiensis	whole organism	48	48
Amphipoda	Talitrus saltator	brain	2	2
Decapoda	Astacus astacus	hepatopancreas, ovaries, green glands, abdominal musculature	14	14
Decapoda	Astacus leptodactylus_1	hypodermis; Y organ	15	
Decapoda	Astacus leptodactylus_2	hepatopancreas	21	
Decapoda	Astacus leptodactylus_3	hypodermis, Y organ, hepatopancreas, gills, hematocytes, muscle	19	41
Decapoda	Callinectes sapidus	gill 7	6	6
Decapoda	Cancer borealis	nervous system	11	11
Decapoda	Carcinus maenas	NA	19	19
Decapoda	Cherax quadricarinatus_1	hypodermis and gastrolith disc	20	
Decapoda	Cherax quadricarinatus_2	heart, kidney, liver, nerve, testis	27	
Decapoda	Cherax quadricarinatus_3	heart, kidney, liver, nerve, testis	27	42
Decapoda	Eriocheir sinensis_1	NA	17	
Decapoda	Eriocheir sinensis_2	eyestalk, Y-organ, and hepatopancreas	21	
Decapoda	Eriocheir sinensis_3	hepatopancreas	17	29
Decapoda	Farfantepenaeus aztecus	hepatopancreas	45	45
Decapoda	Homarus americanus	nervous system	13	13
Decapoda	Hyas araneus_1	adult	4	
Decapoda	Hyas araneus_2	gill	6	9
Decapoda	Litopenaeus vannamei_1	Ghaffari et al., 2014	50	
Decapoda	Litopenaeus vannamei_2	hepatopancreas	49	
Decapoda	Litopenaeus vannamei_3	hepatopancreas	49	
Decapoda	Litopenaeus vannamei_4	hemocytes	23	65
Decapoda	Macrobrachium nipponense	NA	13	13
Decapoda	Pacifastacus leniusculus	Brain, HPT, Hemocyte, Hepatopancreas	8	8
Decapoda	Palaemon argentinus	whole organism	31	31
Decapoda	Penaeus monodon_1	hepatopancreas	118	
Decapoda	Penaeus monodon_2	hepatopancreas	122	46
Decapoda	Procambarus clarkii_1	Eyestalk	11	
Decapoda	Procambarus clarkii_2	Eyestalk, brain, hemocytes, gills, testis, ovary, hepatopancreas, heart, green gland, ventralganglia, Y-organ, hypodermis, muscle	17	24
Decapoda	Scylla olivacea	Na	9	9
Decapoda	Scylla paramamosain	gill	1	1
Euphausiacea	Euphausia superba	NA	33	33
Euphausiacea	Meganyctiphanes norvegica	adult	38	38
Isopoda	Asellus aquaticus	NA	2	2
Isopoda	Bragasellus molinai	whole organism	9	9
Isopoda	Bragasellus peltatus	whole organism	13	13
Isopoda	Proasellus aragonensis	whole organism	14	14
Isopoda	Proasellus arthrodilus	whole organism	16	16
Isopoda	Proasellus assaforensis	whole organism	11	11
Isopoda	Proasellus beticus	whole organism	9	9
Isopoda	Proasellus cantabricus	whole organism	20	20
Isopoda	Proasellus cavaticus	whole organism	19	19

Isopoda	Proasellus coiffaiti	whole organism	18	18
Isopoda	Proasellus coxalis	whole organism	15	15
Isopoda	Proasellus ebrensis	whole organism	15	15
Isopoda	Proasellus escolai	whole organism	13	13
Isopoda	Proasellus grafi	whole organism	15	15
Isopoda	Proasellus granadensis	whole organism	9	9
Isopoda	Proasellus hercegovinensis	whole organism	15	15
Isopoda	Proasellus ibericus	whole organism	13	13
Isopoda	Proasellus jaloniacus	whole organism	16	16
Isopoda	Proasellus karamani	whole organism	12	12
Isopoda	Proasellus margalefi	whole organism	12	12
Isopoda	Proasellus meridianus	whole organism	14	14
Isopoda	Proasellus ortizi	whole organism	12	12
Isopoda	Proasellus parvulus	whole organism	7	7
Isopoda	Proasellus racovitzai	whole organism	10	10
Isopoda	Proasellus rectus	whole organism	17	17
Isopoda	Proasellus solanasi	whole organism	19	19
Isopoda	Proasellus spelaeus	whole organism	15	15
Mysida	Neomysis awatschensis	whole organism	17	17
			**Total malacostracan genes**	1005

Additional file 3G. Scavenger receptor class A.

Arthropoda

Class (subphylum)	Species	Tissue type	Total gene counts	References
Insecta	Drosophila melanogaster	whole organism	5	McTaggart et al., 2009
Insecta	Anopheles gambiae	whole organism	5	McTaggart et al., 2009
Insecta	Aedes aegypti	whole organism	5	McTaggart et al., 2009
Chilopoda (Myriapoda)	Strigamia maritima	whole organism	4	proteome
Arachnida (Chelicerata)	Mesobuthus martensii	whole organism	4	proteome
Arachnida (Chelicerata)	Ixodes scapularis	whole organism	3	proteome
Branchiopoda (Crustacea)	Daphnia pulex	whole organism	6	McTaggart et al., 2009

Malacostraca

Order	Species/Datasets	Tissue type	Total gene counts	Total number of non-redundant genes per species
Amphipoda	Echinogammarus veneris	NA	0	0
Amphipoda	Gammarus chevreuxi	NA	0	0
Amphipoda	Gammarus pulex	NA	0	0
Amphipoda	Hyalella azteca_1	NA	0	
Amphipoda	Hyalella azteca_2	NA	0	
Amphipoda	Hyalella azteca_3	whole organism	2	2
Amphipoda	Melita plumulosa	whole organism	2	2
Amphipoda	Parhyale hawaiensis	whole organism	4	4
Amphipoda	Talitrus saltator	brain	2	2
Decapoda	Astacus astacus	hepatopancreas, ovaries, green glands, abdominal musculature	0	0
Decapoda	Astacus leptodactylus_1	hypodermis; Y organ	3	
Decapoda	Astacus leptodactylus_2	hepatopancreas	2	
Decapoda	Astacus leptodactylus_3	hypodermis, Y organ, hepatopancreas, gills, hematocytes, muscle	4	4
Decapoda	Callinectes sapidus	gill 7	1	1
Decapoda	Cancer borealis	nervous system	2	2
Decapoda	Carcinus maenas	NA	3	3
Decapoda	Cherax quadricarinatus_1	hypodermis and gastrolith disc	0	
Decapoda	Cherax quadricarinatus_2	heart, kidney, liver, nerve, testis	4	
Decapoda	Cherax quadricarinatus_3	heart, kidney, liver, nerve, testis	4	4
Decapoda	Eriocheir sinensis_1	NA	5	
Decapoda	Eriocheir sinensis_2	eyestalk, Y-organ, and hepatopancreas	2	
Decapoda	Eriocheir sinensis_3	hepatopancreas	1	5
Decapoda	Farfantepenaeus aztecus	hepatopancreas	2	2
Decapoda	Homarus americanus	nervous system	3	3
Decapoda	Hyas araneus_1	adult	0	
Decapoda	Hyas araneus_2	gill	3	3
Decapoda	Litopenaeus vannamei_1	Ghaffari et al., 2014	3	
Decapoda	Litopenaeus vannamei_2	hepatopancreas	3	
Decapoda	Litopenaeus vannamei_3	hepatopancreas	2	
Decapoda	Litopenaeus vannamei_4	hemocytes	1	3
Decapoda	Macrobrachium nipponense	NA	3	3
Decapoda	Pacifastacus leniusculus	Brain, HPT, Hemocyte, Hepatopancreas	0	0
Decapoda	Palaemon argentinus	whole organism	2	2
Decapoda	Penaeus monodon_1	hepatopancreas	3	
Decapoda	Penaeus monodon_2	hepatopancreas	4	4
Decapoda	Procambarus clarkii_1	Eyestalk	3	
Decapoda	Procambarus clarkii_2	Eyestalk, brain, hemocytes, gills, testis, ovary, hepatopancreas, heart, green gland, ventralganglia, Y-organ, hypodermis, muscle	3	3
Decapoda	Scylla olivacea	Na	3	3
Decapoda	Scylla paramamosain	gill	1	1
Euphausiacea	Euphausia superba	NA	0	0
Euphausiacea	Meganyctiphanes norvegica	adult	2	2
Isopoda	Asellus aquaticus	NA	0	0
Isopoda	Bragasellus molinai	whole organism	2	2
Isopoda	Bragasellus peltatus	whole organism	4	4
Isopoda	Proasellus aragonensis	whole organism	3	3
Isopoda	Proasellus arthrodilus	whole organism	2	2
Isopoda	Proasellus assaforensis	whole organism	2	2
Isopoda	Proasellus beticus	whole organism	1	1
Isopoda	Proasellus cantabricus	whole organism	5	5
Isopoda	Proasellus cavaticus	whole organism	5	5
Isopoda	Proasellus coiffaiti	whole organism	3	3
Isopoda	Proasellus coxalis	whole organism	3	3

Isopoda	Proasellus ebrensis	whole organism	2	2
Isopoda	Proasellus escolai	whole organism	1	1
Isopoda	Proasellus grafi	whole organism	3	3
Isopoda	Proasellus granadensis	whole organism	2	2
Isopoda	Proasellus hercegovinensis	whole organism	4	4
Isopoda	Proasellus ibericus	whole organism	1	1
Isopoda	Proasellus jaloniacus	whole organism	2	2
Isopoda	Proasellus karamani	whole organism	4	4
Isopoda	Proasellus margalefi	whole organism	0	0
Isopoda	Proasellus meridianus	whole organism	4	4
Isopoda	Proasellus ortizi	whole organism	4	4
Isopoda	Proasellus parvulus	whole organism	3	3
Isopoda	Proasellus racovitzai	whole organism	3	3
Isopoda	Proasellus rectus	whole organism	4	4
Isopoda	Proasellus solanasi	whole organism	2	2
Isopoda	Proasellus spelaeus	whole organism	1	1
Mysida	Neomysis awatschensis	whole organism	1	1
			**Total malacostracan genes**	129

Additional file 3H. Scavenger receptor class B.

Arthropoda

Class (subphylum)	Species	Tissue type	Total gene counts	References
Insecta	Drosophila melanogaster	whole organism	13	ImmunoDB
Insecta	Anopheles gambiae	whole organism	14	ImmunoDB
Insecta	Aedes aegypti	whole organism	13	ImmunoDB
Chilopoda (Myriapoda)	Strigamia maritima	whole organism	7	proteome
Arachnida (Chelicerata)	Mesobuthus martensii	whole organism	4	proteome
Arachnida (Chelicerata)	Ixodes scapularis	whole organism	3	proteome
Branchiopoda (Crustacea)	Daphnia pulex	whole organism	8	proteome

Malacostraca

Order	Species/Datasets	Tissue type	Total gene counts	Total number of non-redundant genes per species
Amphipoda	Echinogammarus veneris	NA	1	1
Amphipoda	Gammarus chevreuxi	NA	3	3
Amphipoda	Gammarus pulex	NA	8	8
Amphipoda	Hyalella azteca_1	NA	1	
Amphipoda	Hyalella azteca_2	NA	2	
Amphipoda	Hyalella azteca_3	whole organism	1	2
Amphipoda	Melita plumulosa	whole organism	5	5
Amphipoda	Parhyale hawaiensis	whole organism	8	8
Amphipoda	Talitrus saltator	brain	5	5
Decapoda	Astacus astacus	hepatopancreas, ovaries, green glands, abdominal musculature	3	3
Decapoda	Astacus leptodactylus_1	hypodermis; Y organ	6	
Decapoda	Astacus leptodactylus_2	hepatopancreas	6	
Decapoda	Astacus leptodactylus_3	hypodermis, Y organ, hepatopancreas, gills, hematocytes, muscle	7	7
Decapoda	Callinectes sapidus	gill 7	2	2
Decapoda	Cancer borealis	nervous system	6	6
Decapoda	Carcinus maenas	NA	7	7
Decapoda	Cherax quadricarinatus_1	hypodermis and gastrolith disc	2	
Decapoda	Cherax quadricarinatus_2	heart, kidney, liver, nerve, testis	5	
Decapoda	Cherax quadricarinatus_3	heart, kidney, liver, nerve, testis	7	7
Decapoda	Eriocheir sinensis_1	NA	5	
Decapoda	Eriocheir sinensis_2	eyestalk, Y-organ, and hepatopancreas	3	
Decapoda	Eriocheir sinensis_3	hepatopancreas	4	5
Decapoda	Farfantepenaeus aztecus	hepatopancreas	8	8
Decapoda	Homarus americanus	nervous system	8	8
Decapoda	Hyas araneus_1	adult	3	
Decapoda	Hyas araneus_2	gill	7	7
Decapoda	Litopenaeus vannamei_1	Ghaffari et al., 2014	7	
Decapoda	Litopenaeus vannamei_2	hepatopancreas	7	
Decapoda	Litopenaeus vannamei_3	hepatopancreas	6	
Decapoda	Litopenaeus vannamei_4	hemocytes	3	7
Decapoda	Macrobrachium nipponense	NA	4	4
Decapoda	Pacifastacus leniusculus	Brain, HPT, Hemocyte, Hepatopancreas	5	5
Decapoda	Palaemon argentinus	whole organism	1	1
Decapoda	Penaeus monodon_1	hepatopancreas	7	
Decapoda	Penaeus monodon_2	hepatopancreas	8	8
Decapoda	Procambarus clarkii_1	Eyestalk	5	
Decapoda	Procambarus clarkii_2	Eyestalk, brain, hemocytes, gills, testis, ovary, hepatopancreas, heart, green gland, ventralganglia, Y-organ, hypodermis, muscle	6	6
Decapoda	Scylla olivacea	Na	6	6
Decapoda	Scylla paramamosain	gill	1	1
Euphausiacea	Euphausia superba	NA	6	6
Euphausiacea	Meganyctiphanes norvegica	adult	1	1
Isopoda	Asellus aquaticus	NA	2	2
Isopoda	Bragasellus molinai	whole organism	7	7
Isopoda	Bragasellus peltatus	whole organism	6	6
Isopoda	Proasellus aragonensis	whole organism	7	7
Isopoda	Proasellus arthrodilus	whole organism	7	7
Isopoda	Proasellus assaforensis	whole organism	6	6
Isopoda	Proasellus beticus	whole organism	4	4
Isopoda	Proasellus cantabricus	whole organism	7	7
Isopoda	Proasellus cavaticus	whole organism	8	8

Isopoda	Proasellus coiffaiti	whole organism	7	7
Isopoda	Proasellus coxalis	whole organism	6	6
Isopoda	Proasellus ebrensis	whole organism	8	8
Isopoda	Proasellus escolai	whole organism	6	6
Isopoda	Proasellus grafi	whole organism	7	7
Isopoda	Proasellus granadensis	whole organism	5	5
Isopoda	Proasellus hercegovinensis	whole organism	5	5
Isopoda	Proasellus ibericus	whole organism	7	7
Isopoda	Proasellus jaloniacus	whole organism	7	7
Isopoda	Proasellus karamani	whole organism	9	9
Isopoda	Proasellus margalefi	whole organism	5	5
Isopoda	Proasellus meridianus	whole organism	7	7
Isopoda	Proasellus ortizi	whole organism	10	10
Isopoda	Proasellus parvulus	whole organism	6	6
Isopoda	Proasellus racovitzai	whole organism	7	7
Isopoda	Proasellus rectus	whole organism	7	7
Isopoda	Proasellus solanasi	whole organism	8	8
Isopoda	Proasellus spelaeus	whole organism	5	5
Mysida	Neomysis awatschensis	whole organism	1	1
			**Total malacostracan genes**	314

Additional file 3I. CLIP-domain serine proteases.

Arthropoda

Class (subphylum)	Species	Tissue type	Total gene counts	References
Insecta	Drosophila melanogaster	whole organism	47	ImmunoDB
Insecta	Anopheles gambiae	whole organism	55	ImmunoDB
Insecta	Aedes aegypti	whole organism	69	ImmunoDB
Chilopoda (Myriapoda)	Strigamia maritima	whole organism	4	Palmer et al., 2015
Arachnida (Chelicerata)	Mesobuthus martensii	whole organism	7	Palmer et al., 2015
Arachnida (Chelicerata)	Ixodes scapularis	whole organism	4	Palmer et al., 2015
Branchiopoda (Crustacea)	Daphnia pulex	whole organism	3	Palmer et al., 2015

Malacostraca

Order	Species/Datasets	Tissue type	Total gene counts	Total number of non-redundant genes per species
Amphipoda	Echinogammarus veneris	NA	14	14
Amphipoda	Gammarus chevreuxi	NA	36	36
Amphipoda	Gammarus pulex	NA	10	10
Amphipoda	Hyalella azteca_1	NA	3	
Amphipoda	Hyalella azteca_2	NA	1	
Amphipoda	Hyalella azteca_3	whole organism	16	16
Amphipoda	Melita plumulosa	whole organism	21	21
Amphipoda	Parhyale hawaiensis	whole organism	54	54
Amphipoda	Talitrus saltator	brain	21	21
Decapoda	Astacus astacus	hepatopancreas, ovaries, green glands, abdominal musculature	10	10
Decapoda	Astacus leptodactylus_1	hypodermis; Y organ	40	
Decapoda	Astacus leptodactylus_2	hepatopancreas	23	
Decapoda	Astacus leptodactylus_3	hypodermis, Y organ, hepatopancreas, gills, hematocytes, muscle	60	60
Decapoda	Callinectes sapidus	gill 7	23	23
Decapoda	Cancer borealis	nervous system	27	27
Decapoda	Carcinus maenas	NA	40	40
Decapoda	Cherax quadricarinatus_1	hypodermis and gastrolith disc	7	
Decapoda	Cherax quadricarinatus_2	heart, kidney, liver, nerve, testis	14	
Decapoda	Cherax quadricarinatus_3	heart, kidney, liver, nerve, testis	16	16
Decapoda	Eriocheir sinensis_1	NA	54	
Decapoda	Eriocheir sinensis_2	eyestalk, Y-organ, and hepatopancreas	12	
Decapoda	Eriocheir sinensis_3	hepatopancreas	13	54
Decapoda	Farfantepenaeus aztecus	hepatopancreas	16	16
Decapoda	Homarus americanus	nervous system	29	29
Decapoda	Hyas araneus_1	adult	16	
Decapoda	Hyas araneus_2	gill	18	18
Decapoda	Litopenaeus vannamei_1	Ghaffari et al., 2014	72	
Decapoda	Litopenaeus vannamei_2	hepatopancreas	24	
Decapoda	Litopenaeus vannamei_3	hepatopancreas	25	
Decapoda	Litopenaeus vannamei_4	hemocytes	26	72
Decapoda	Macrobrachium nipponense	NA	53	53
Decapoda	Pacifastacus leniusculus	Brain, HPT, Hemocyte, Hepatopancreas	12	12
Decapoda	Palaemon argentinus	whole organism	12	12
Decapoda	Penaeus monodon_1	hepatopancreas	25	
Decapoda	Penaeus monodon_2	hepatopancreas	24	25
Decapoda	Procambarus clarkii_1	Eyestalk	37	
Decapoda	Procambarus clarkii_2	Eyestalk, brain, hemocytes, gills, testis, ovary, hepatopancreas, heart, green gland, ventralganglia, Y-organ, hypodermis, muscle	44	44
Decapoda	Scylla olivacea	Na	41	41
Decapoda	Scylla paramamosain	gill	37	37
Euphausiacea	Euphausia superba	NA	47	47
Euphausiacea	Meganyctiphanes norvegica	adult	57	57
Isopoda	Asellus aquaticus	NA	17	17
Isopoda	Bragasellus molinai	whole organism	21	21
Isopoda	Bragasellus peltatus	whole organism	48	48
Isopoda	Proasellus aragonensis	whole organism	49	49
Isopoda	Proasellus arthrodilus	whole organism	60	60
Isopoda	Proasellus assaforensis	whole organism	43	43
Isopoda	Proasellus beticus	whole organism	22	22
Isopoda	Proasellus cantabricus	whole organism	51	51
Isopoda	Proasellus cavaticus	whole organism	60	60
Isopoda	Proasellus coiffaiti	whole organism	62	62
Isopoda	Proasellus coxalis	whole organism	59	59

Isopoda	Proasellus ebrensis	whole organism	44	44
Isopoda	Proasellus escolai	whole organism	27	27
Isopoda	Proasellus grafi	whole organism	42	42
Isopoda	Proasellus granadensis	whole organism	19	19
Isopoda	Proasellus hercegovinensis	whole organism	40	40
Isopoda	Proasellus ibericus	whole organism	41	41
Isopoda	Proasellus jaloniacus	whole organism	62	62
Isopoda	Proasellus karamani	whole organism	61	61
Isopoda	Proasellus margalefi	whole organism	24	24
Isopoda	Proasellus meridianus	whole organism	68	68
Isopoda	Proasellus ortizi	whole organism	46	46
Isopoda	Proasellus parvulus	whole organism	42	42
Isopoda	Proasellus racovitzai	whole organism	58	58
Isopoda	Proasellus rectus	whole organism	54	54
Isopoda	Proasellus solanasi	whole organism	57	57
Isopoda	Proasellus spelaeus	whole organism	65	65
Mysida	Neomysis awatschensis	whole organism	56	56
			**Total malacostracan genes**	2163

Additional file 3J. Prophenoloxidase and hemocyanin.
Arthropoda

Class (subphylum)	Species	Tissue type	Prophenoloxidase	References
Insecta	Drosophila melanogaster	whole organism	3	McTaggart et al., 2009
Insecta	Anopheles gambiae	whole organism	9	McTaggart et al., 2009
Insecta	Aedes aegypti	whole organism	10	McTaggart et al., 2009
Chilopoda (Myriapoda)	Strigamia maritima	whole organism	0	Palmer et al., 2009
Arachnida (Chelicerata)	Mesobuthus martensii	whole organism	0	Palmer et al., 2009
Arachnida (Chelicerata)	Ixodes scapularis	whole organism	0	Palmer et al., 2009
Branchiopoda (Crustacea)	Daphnia pulex	whole organism	1	McTaggart et al., 2009

Malacostraca

Order	Species/Datasets	Tissue type	Prophenoloxidase	Hemocyanin
Amphipoda	Echinogammarus veneris	NA	0	2
Amphipoda	Gammarus chevreuxi	NA	1	3
Amphipoda	Gammarus pulex	NA	1	11
Amphipoda	Hyalella azteca_1	NA	0	5
Amphipoda	Hyalella azteca_2	NA	0	2
Amphipoda	Hyalella azteca_3	whole organism	1	1
Amphipoda	Melita plumulosa	whole organism	1	5
Amphipoda	Parhyale hawaiensis	whole organism	1	7
Amphipoda	Talitrus saltator	brain	1	2
Decapoda	Astacus astacus	hepatopancreas, ovaries, green glands, abdominal musculature	1	3
Decapoda	Astacus leptodactylus_1	hypodermis; Y organ	1	6
Decapoda	Astacus leptodactylus_2	hepatopancreas	1	4
Decapoda	Astacus leptodactylus_3	hypodermis, Y organ, hepatopancreas, gills, hematocytes, muscle	2	7
Decapoda	Callinectes sapidus	gill 7	0	0
Decapoda	Cancer borealis	nervous system	1	4
Decapoda	Carcinus maenas	NA	0	5
Decapoda	Cherax quadricarinatus_1	hypodermis and gastrolith disc	1	6
Decapoda	Cherax quadricarinatus_2	heart, kidney, liver, nerve, testis	1	4
Decapoda	Cherax quadricarinatus_3	heart, kidney, liver, nerve, testis	1	7
Decapoda	Eriocheir sinensis_1	NA	1	5
Decapoda	Eriocheir sinensis_2	eyestalk, Y-organ, and hepatopancreas	1	4
Decapoda	Eriocheir sinensis_3	hepatopancreas	1	3
Decapoda	Farfantepenaeus aztecus	hepatopancreas	1	7
Decapoda	Homarus americanus	nervous system	1	2
Decapoda	Hyas araneus_1	adult	1	0
Decapoda	Hyas araneus_2	gill	1	0
Decapoda	Litopenaeus vannamei_1	Ghaffari et al., 2014	2	6
Decapoda	Litopenaeus vannamei_2	hepatopancreas	3	2
Decapoda	Litopenaeus vannamei_3	hepatopancreas	3	7
Decapoda	Litopenaeus vannamei_4	hemocytes	2	4
Decapoda	Macrobrachium nipponense	NA	1	2
Decapoda	Pacifastacus leniusculus	Brain, HPT, Hemocyte, Hepatopancreas	1	2
Decapoda	Palaemon argentinus	whole organism	1	5
Decapoda	Penaeus monodon_1	hepatopancreas	2	9
Decapoda	Penaeus monodon_2	hepatopancreas	2	7
Decapoda	Procambarus clarkii_1	Eyestalk	1	1
Decapoda	Procambarus clarkii_2	Eyestalk, brain, hemocytes, gills, testis, ovary, hepatopancreas, heart, green gland, ventralganglia, Y-organ, hypodermis, muscle	1	2
Decapoda	Scylla olivacea	Na	0	3
Decapoda	Scylla paramamosain	gill	2	0
Euphausiacea	Euphausia superba	NA	1	9
Euphausiacea	Meganyctiphanes norvegica	adult	0	7
Isopoda	Asellus aquaticus	NA	0	2
Isopoda	Bragasellus molinai	whole organism	0	6
Isopoda	Bragasellus peltatus	whole organism	1	4
Isopoda	Proasellus aragonensis	whole organism	1	7
Isopoda	Proasellus arthrodilus	whole organism	1	8
Isopoda	Proasellus assaforensis	whole organism	1	7
Isopoda	Proasellus beticus	whole organism	0	4
Isopoda	Proasellus cantabricus	whole organism	1	9
Isopoda	Proasellus cavaticus	whole organism	1	4

Isopoda	Proasellus coiffaiti	whole organism	1	7
Isopoda	Proasellus coxalis	whole organism	1	5
Isopoda	Proasellus ebrensis	whole organism	0	10
Isopoda	Proasellus escolai	whole organism	1	7
Isopoda	Proasellus grafi	whole organism	1	7
Isopoda	Proasellus granadensis	whole organism	0	5
Isopoda	Proasellus hercegovinensis	whole organism	1	4
Isopoda	Proasellus ibericus	whole organism	1	10
Isopoda	Proasellus jaloniacus	whole organism	1	5
Isopoda	Proasellus karamani	whole organism	1	6
Isopoda	Proasellus margalefi	whole organism	0	8
Isopoda	Proasellus meridianus	whole organism	1	7
Isopoda	Proasellus ortizi	whole organism	1	5
Isopoda	Proasellus parvulus	whole organism	1	7
Isopoda	Proasellus racovitzai	whole organism	1	7
Isopoda	Proasellus rectus	whole organism	1	8
Isopoda	Proasellus solanasi	whole organism	1	5
Isopoda	Proasellus spelaeus	whole organism	1	7
Mysida	Neomysis awatschensis	whole organism	1	5

	hemocyanin	Prophenoloxidase
Echinogammarus veneris	2	0
Gammarus chevreuxi	3	1
Gammarus pulex	11	1
Hyalella azteca	5	1
Melita plumulosa	5	1
Parhyale hawaiensis	7	1
Talitrus saltator	2	1
Astacus astacus	3	1
Astacus leptodactylus	7	2
Callinectes sapidus	0	0
Cancer borealis	4	1
Carcinus maenas	5	0
Cherax quadricarinatus	7	1
Eriocheir sinensis	5	1
Farfantepenaeus aztecus	7	1
Homarus americanus	2	1
Hyas araneus	0	1
Litopenaeus vannamei	7	2
Macrobrachium nipponense	2	1
Pacifastacus leniusculus	2	1
Palaemon argentinus	5	1
Penaeus monodon	9	2
Procambarus clarkii	2	1
Scylla olivacea	3	0
Scylla paramamosain	0	2
Euphausia superba	9	1
Meganyctiphanes norvegica	7	0
Asellus aquaticus	2	0
Bragasellus molinai	6	0
Bragasellus peltatus	4	1
Proasellus aragonensis	7	1
Proasellus arthrodilus	8	1
Proasellus assaforensis	7	1
Proasellus beticus	4	0
Proasellus cantabricus	9	1
Proasellus cavaticus	4	1
Proasellus coiffaiti	7	1
Proasellus coxalis	5	1
Proasellus ebrensis	10	0
Proasellus escolai	7	1
Proasellus grafi	7	1
Proasellus granadensis	5	0
Proasellus hercegovinensis	4	1
Proasellus ibericus	10	1
Proasellus jaloniacus	5	1
Proasellus karamani	6	1
Proasellus margalefi	8	0
Proasellus meridianus	7	1
Proasellus ortizi	5	1
Proasellus parvulus	7	1
Proasellus racovitzai	7	1
Proasellus rectus	8	1
Proasellus solanasi	5	1
Proasellus spelaeus	7	1
Neomysis awatschensis	5	1
	297	48
