# Supplementary material for: Comparative genomic analysis of innate immunity reveals novel and conserved components in crustacean food crop species
Source: BMC Genomics. 2017 May 18;18:389. doi: 10.1186/s12864-017-3769-4 (PMC5437397; doi:10.1186/s12864-017-3769-4)
Supplement: Supplementary file 5 — Multiple sequence alignment of the β-glucanase domains of Gram negative binding proteins of malacostracans together with the β-glucanase protein from Bombyx mori (NP_001159614.1). Two conserved Glu active site residues are labeled as E188 and E193 based on positions in the B. mori protein. (PDF 924 kb) [file 12864_2017_3769_MOESM5_ESM.pdf]

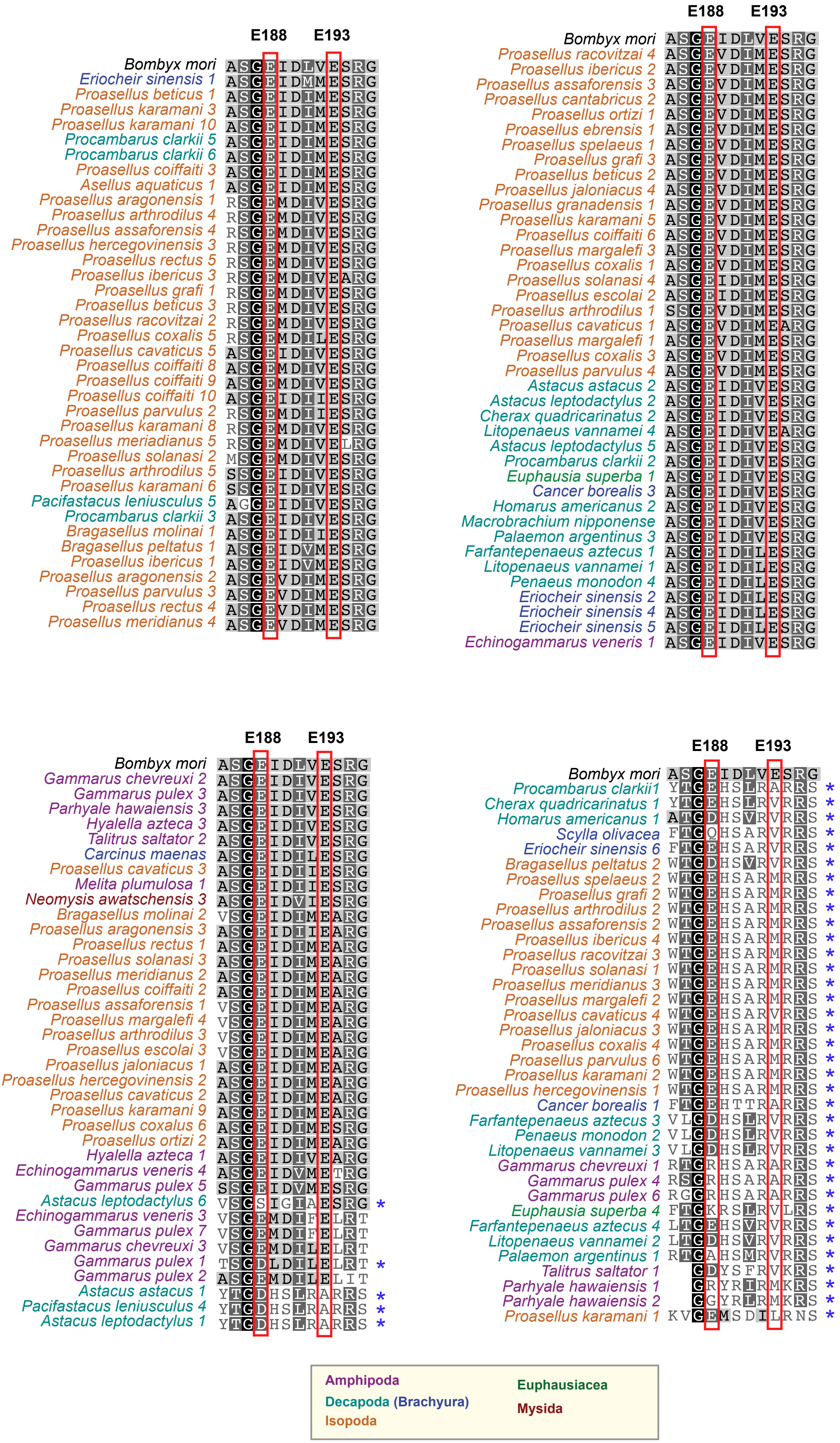

E188 E193
Bombyx mori ASGEIDLVESRG
Eriocheir sinensis 1 ASGEIDMMESRG
Proasellus beticus 1 ASGEIDIMESRG
Proasellus karamani 3 ASGEIDIMESRG
Proasellus karamani 10 ASGEIDIMESRG
Procambarus clarkii 5 ASGEIDIMESRG
Procambarus clarkii 6 ASGEIDIMESRG
Proasellus coiffaiti 3 ASGEIDIMESRG
Asellus aquaticus 1 ASGEIDIMESRG
Proasellus aragonensis 1 RSGEMDIVESRG
Proasellus arthrodilus 4 RSGEMDIVESRG
Proasellus assaforensis 4 RSGEMDIVESRG
Proasellus hercegovinensis 3 RSGEMDIVESRG
Proasellus rectus 5 RSGEMDIVESRG
Proasellus ibericus 3 RSGEMDIVEARG
Proasellus grafi 1 RSGEMDIVESRG
Proasellus beticus 3 RSGEMDIVESRG
Proasellus racovitzai 2 RSGEMDIVESRG
Proasellus coxalis 5 RSGEMDILESRG
Proasellus cavaticus 5 ASGEIDIVESRG
Proasellus coiffaiti 8 ASGEMDIVESRG
Proasellus coiffaiti 9 ASGEMDIVESRG
Proasellus coiffaiti 10 ASGEIDIIESRG
Proasellus parvulus 2 RSGEMDIIESRG
Proasellus karamani 8 RSGEMDIVESRG
Proasellus meriadianus 5 RSGEMDIVELRG
Proasellus solanasi 2 MSGEMDIVESRG
Proasellus arthrodilus 5 SSGEIDIVESRG
Proasellus karamani 6 SSGEIDIVESRG
Pacifastacus leniusculus 5 AGGEIDIVESRG
Procambarus clarkii 3 ASGEIDIVESRG
Bragasellus molinai 1 ASGEIDIIESRG
Bragasellus peltatus 1 ASGEIDVMESRG
Proasellus ibericus 1 ASGEIDVMESRG
Proasellus aragonensis 2 ASGEVDIMESRG
Proasellus parvulus 3 ASGEVDIMESRG
Proasellus rectus 4 ASGEVDIMESRG
Proasellus meridianus 4 ASGEVDIMESRG
E188 E193
Bombyx mori ASGEIDLVESRG
Proasellus racovitzai 4 ASGEVDIMESRG
Proasellus ibericus 2 ASGEVDIMESRG
Proasellus assaforensis 3 ASGEVDIMESRG
Proasellus cantabricus 2 ASGEVDIMESRG
Proasellus ortizi 1 ASGEVDIMESRG
Proasellus ebrensis 1 ASGEVDIMESRG
Proasellus spelaeus 1 ASGEVDIMESRG
Proasellus grafi 3 ASGEVDIMESRG
Proasellus beticus 2 ASGEVDIMESRG
Proasellus jaloniacus 4 ASGEVDIMESRG
Proasellus granadensis 1 ASGEVDIMESRG
Proasellus karamani 5 ASGEVDIMESRG
Proasellus coiffaiti 6 ASGEVDIMESRG
Proasellus margalefi 3 ASGEVDIMESRG
Proasellus coxalis 1 ASGEVDIMESRG
Proasellus solanasi 4 ASGEIDIMESRG
Proasellus escolai 2 ASGEIDIMESRG
Proasellus arthrodilus 1 SSGEVDIMESRG
Proasellus cavaticus 1 ASGEVDIMEARG
Proasellus margalefi 1 ASGEVDIMESRG
Proasellus coxalis 3 ASGEVDIMESRG
Proasellus parvulus 4 ASGEVDIMESRG
Astacus astacus 2 ASGEIDIVESRG
Astacus leptodactylus 2 ASGEIDIVESRG
Cherax quadricarinatus 2 ASGEIDIVESRG
Litopenaeus vannamei 4 ASGEIDIVEARG
Astacus leptodactylus 5 ASGEIDIVESRG
Procambarus clarkii 2 ASGEIDIVESRG
Euphausia superba 1 ASGEIDIVESRG
Cancer borealis 3 ASGEIDIVESRG
Homarus americanus 2 ASGEIDIVESRG
Macrobrachium nipponense ASGEIDIVESRG
Palaemon argentinus 3 ASGEIDIVESRG
Farfantepenaeus aztecus 1 ASGEIDILESRG
Litopenaeus vannamei 1 ASGEIDILESRG
Penaeus monodon 4 ASGEIDILESRG
Eriocheir sinensis 2 ASGEIDILESRG
Eriocheir sinensis 4 ASGEIDILESRG
Eriocheir sinensis 5 ASGEIDILESRG
Echinogammarus veneris 1 ASGEIDIVESRG
E188 E193
Bombyx mori ASGEIDLVESRG
Gammarus chevreuxi 2 ASGEIDIVESRG
Gammarus pulex 3 ASGEIDIVESRG
Parhyale hawaiensis 3 ASGEIDIVESRG
Hyalella azteca 3 ASGEIDIVESRG
Talitrus saltator 2 ASGEIDIVESRG
Carcinus maenas ASGEIDILESRG
Proasellus cavaticus 3 ASGEIDIIESRG
Melita plumulosa 1 ASGEIDIIESRG
Neomysis awatschensis 3 ASGEIDVIESRG
Bragasellus molinai 2 VSGEIDIMEARG
Proasellus aragonensis 3 ASGEIDIIEARG
Proasellus rectus 1 ASGEIDIMEARG
Proasellus solanasi 3 ASGEIDIMEARG
Proasellus meridianus 2 ASGEIDIMEARG
Proasellus coiffaiti 2 ASGEIDIMEARG
Proasellus assaforensis 1 VSGEIDIMEARG
Proasellus margalefi 4 VSGEIDIMEARG
Proasellus arthrodilus 3 VSGEIDIMEARG
Proasellus escolai 3 VSGEIDIMEARG
Proasellus jaloniacus 1 ASGEIDIMEARG
Proasellus hercegovinensis 2 ASGEIDIMEARG
Proasellus cavaticus 2 ASGEIDIMEARG
Proasellus karamani 9 ASGEIDIMEARG
Proasellus coxalus 6 ASGEIDIMESRG
Proasellus ortizi 2 ASGEIDIMEARG
Hyalella azteca 1 ASGEIDIVEARG
Echinogammarus veneris 4 ASGEIDVMETRG
Gammarus pulex 5 SSGEIDVMESRG
Astacus leptodactylus 6 VSGSIGIAESRG *
Echinogammarus veneris 3 VSGEMDIFELRT
Gammarus pulex 7 VSGEMDIFELRT
Gammarus chevreuxi 3 VSGEMDILELRT
Gammarus pulex 1 TSGDLDILELRT *
Gammarus pulex 2 ASGEMDILELIT
Astacus astacus 1 YTGDHSLRARRS *
Pacifastacus leniusculus 4 YTGDHSLRARRS *
Astacus leptodactylus 1 YTGDHSLRARRS *
E188 E193
Bombyx mori ASGEIDLVESRG
Procambarus clarkii1 YTGEHSLRARRS *
Cherax quadricarinatus 1 YTGEHSLRVRRS *
Homarus americanus 1 ATGDHSVRVRRS *
Scylla olivacea FTGQHSARVRRS *
Eriocheir sinensis 6 FTGEHSARVRRS *
Bragasellus peltatus 2 WTGDHSVRVRRS *
Proasellus spelaeus 2 WTGEHSARMRRS *
Proasellus grafi 2 WTGEHSARMRRS *
Proasellus arthrodilus 2 WTGEHSARMRRS *
Proasellus assaforensis 2 WTGEHSARMRRS *
Proasellus ibericus 4 WTGEHSARMRRS *
Proasellus racovitzai 3 WTGEHSARMRRS *
Proasellus solanasi 1 WTGEHSARMRRS *
Proasellus meridianus 3 WTGEHSARMRRS *
Proasellus margalefi 2 WTGEHSARMRRS *
Proasellus cavaticus 4 WTGEHSARVRRS *
Proasellus jaloniacus 3 WTGEHSARMRRS *
Proasellus coxalis 4 WTGEHSARMRRS *
Proasellus parvulus 6 WTGEHSARMRRS *
Proasellus karamani 2 WTGEHSARMRRS *
Proasellus hercegovinensis 1 WTGEHSARMRRS *
Cancer borealis 1 FTGEHTTRARRS *
Farfantepenaeus aztecus 3 VLGDHSLRVRRS *
Penaeus monodon 2 VLGDHSLRVRRS *
Litopenaeus vannamei 3 VLGDHSLRVRRS *
Gammarus chevreuxi 1 RTGRHSARARRS *
Gammarus pulex 4 RSGRHSARARRS *
Gammarus pulex 6 RGGRHSARARRS *
Euphausia superba 4 FTGKRSLRVLRS *
Farfantepenaeus aztecus 4 LTGEHSVRVRRS *
Litopenaeus vannamei 2 LTGDHSVRVRRS *
Palaemon argentinus 1 RTGAHSMRVRRS *
Talitrus saltator 1 GDYSFRVKRS *
Parhyale hawaiensis 1 GRYRIRMKRS *
Parhyale hawaiensis 2 GGYRLRMKRS *
Proasellus karamani 1 KVGEMSDILRNS *
Amphipoda
Decapoda (Brachyura)
Isopoda
Euphausiacea
Mysida
