# Supplementary figures and images for: Comparative genomic analysis of innate immunity reveals novel and conserved components in crustacean food crop species
Source: BMC Genomics. 2017 May 18;18:389. doi: 10.1186/s12864-017-3769-4 (PMC5437397; doi:10.1186/s12864-017-3769-4)

A

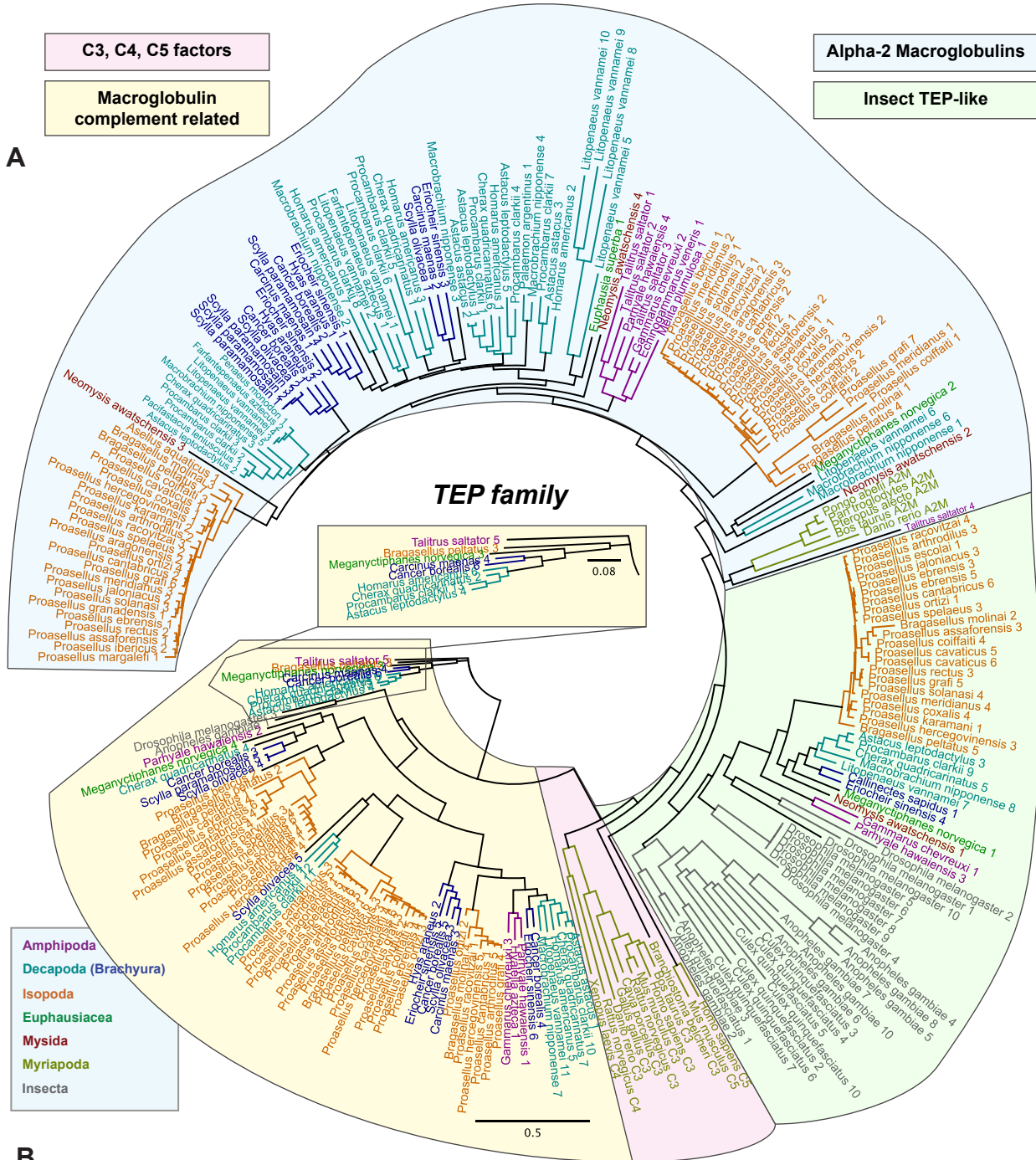

B

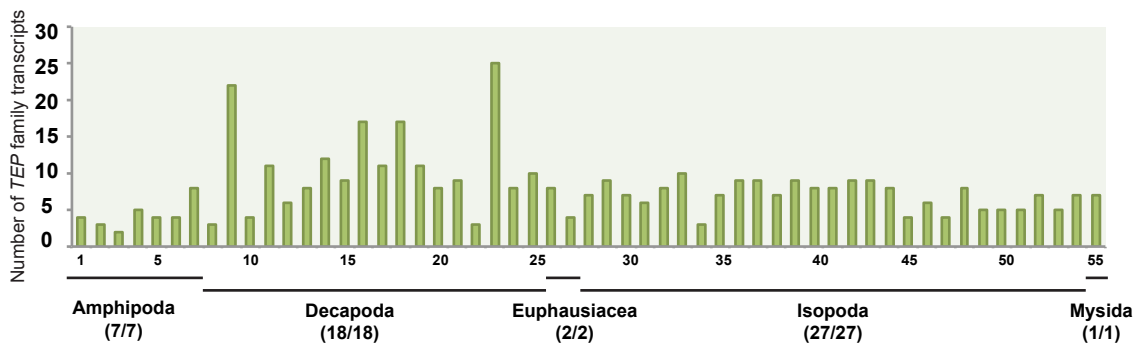

Supplement: Supplementary file 6 — Thioester-containing protein (TEPs) family. (A) Phylogenetic tree of the TEP family is constructed using the maximum-likelihood method from an amino acid multiple sequence alignment. Amino acid sequences include the macroglobulin complement related proteins, the vertebrate C3, C4 and C5 complement factors, arthropod TEPs and the alpha-2 macroglobulin family. Taxa labels are depicted as their respective colour codes. Bootstrap support values (n=1000) for all trees can be found in Supplementary figure 8. Scale bar represents substitution per site. (B) Graph of putative TEP family transcripts. The y-axes represent total number of genes identified in all 55 malacostracan species for each family. Each species is represented by a number on the X-axes and a complete list of species is available in Additional file 3: Table S2. Black horizontal bars below each graph delimit the five orders of malacostracans and the numbers in parentheses (x/y) represent the following: x = number of species in which a particular gene family is found and y = total number of species in each order. (PDF 1763 kb) [file 12864_2017_3769_MOESM6_ESM.pdf]

A

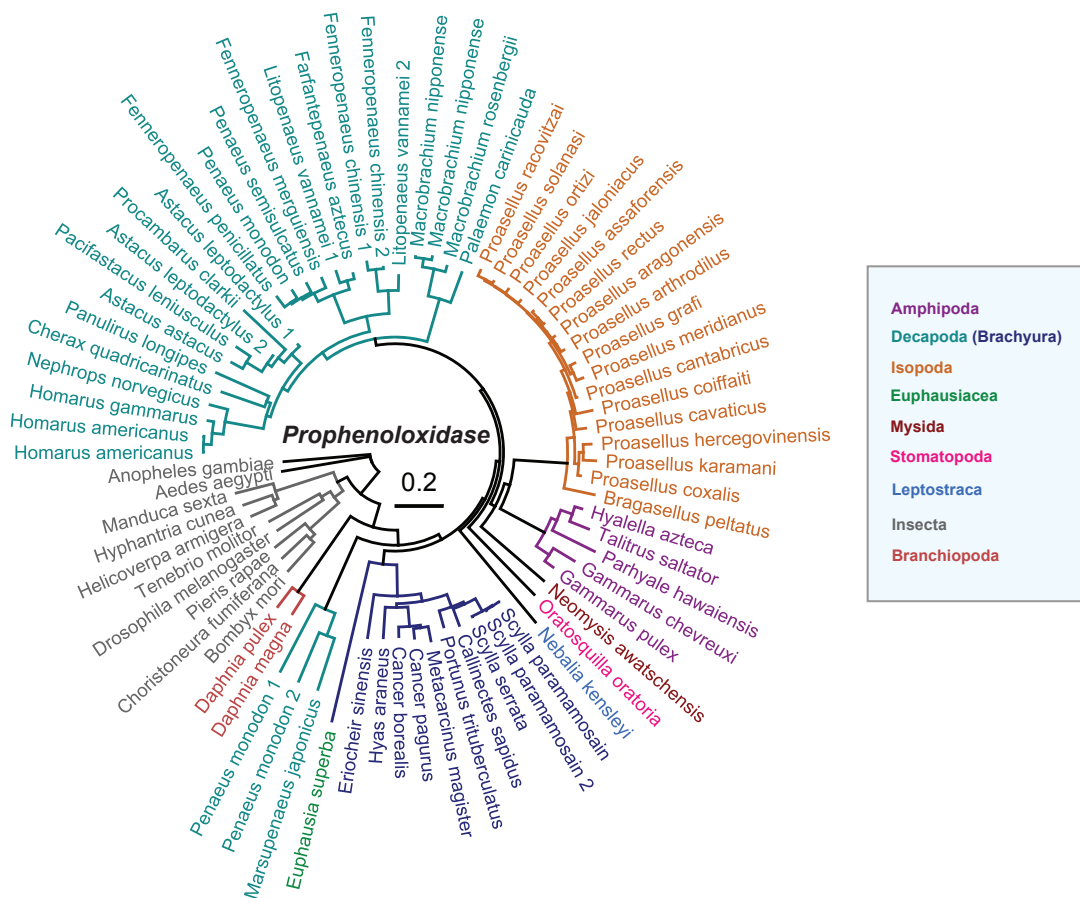

B

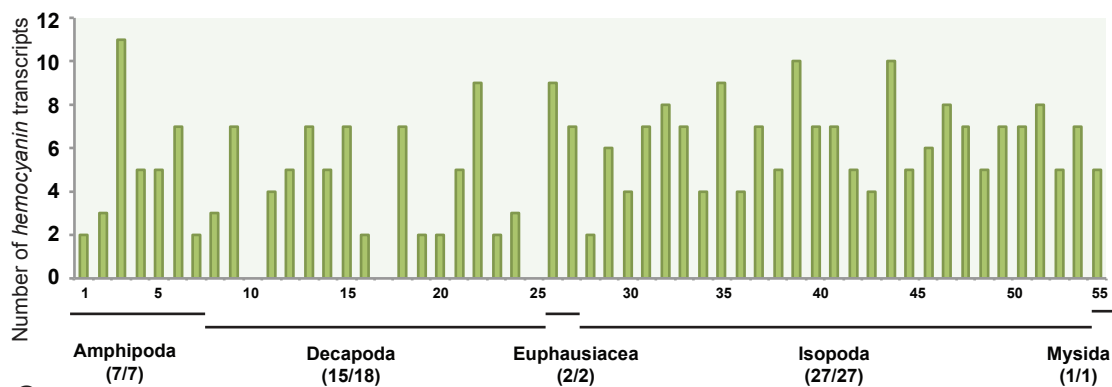

C

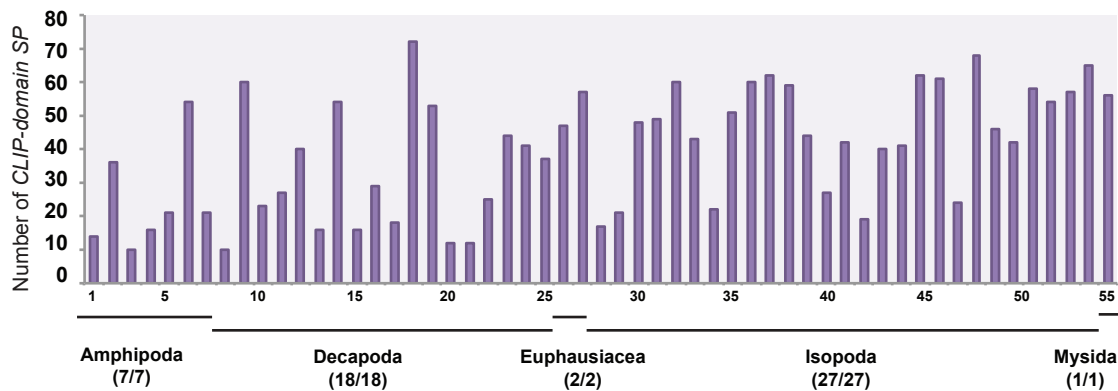

Supplement: Supplementary file 7 — Prophenoloxidase activation system. (A) Phylogenetic tree of prophenoloxidase (proPO) is constructed using the maximum-likelihood method from an amino acid multiple sequence alignment. Taxa labels are depicted as their respective colour codes. Bootstrap support values (n=1000) for all trees can be found in Additional file 14: Figure S8. Scale bar represents substitution per site. The graphs represent the total number of (B) hemocyanin and (C) CLIP-domain serine protease transcripts in malacostracans. The y-axes represent total number of genes identified in all 55 malacostracan species for each family. Each species is represented by a number on the X-axes and a complete list of species is available in Additional file 3: Table S2. Black horizontal bars below each graph delimit the five orders of malacostracans and the numbers in parentheses (x/y) represent the following: x = number of species in which a particular gene family is found and y = total number of species in each order. (PDF 579 kb) [file 12864_2017_3769_MOESM7_ESM.pdf]

A

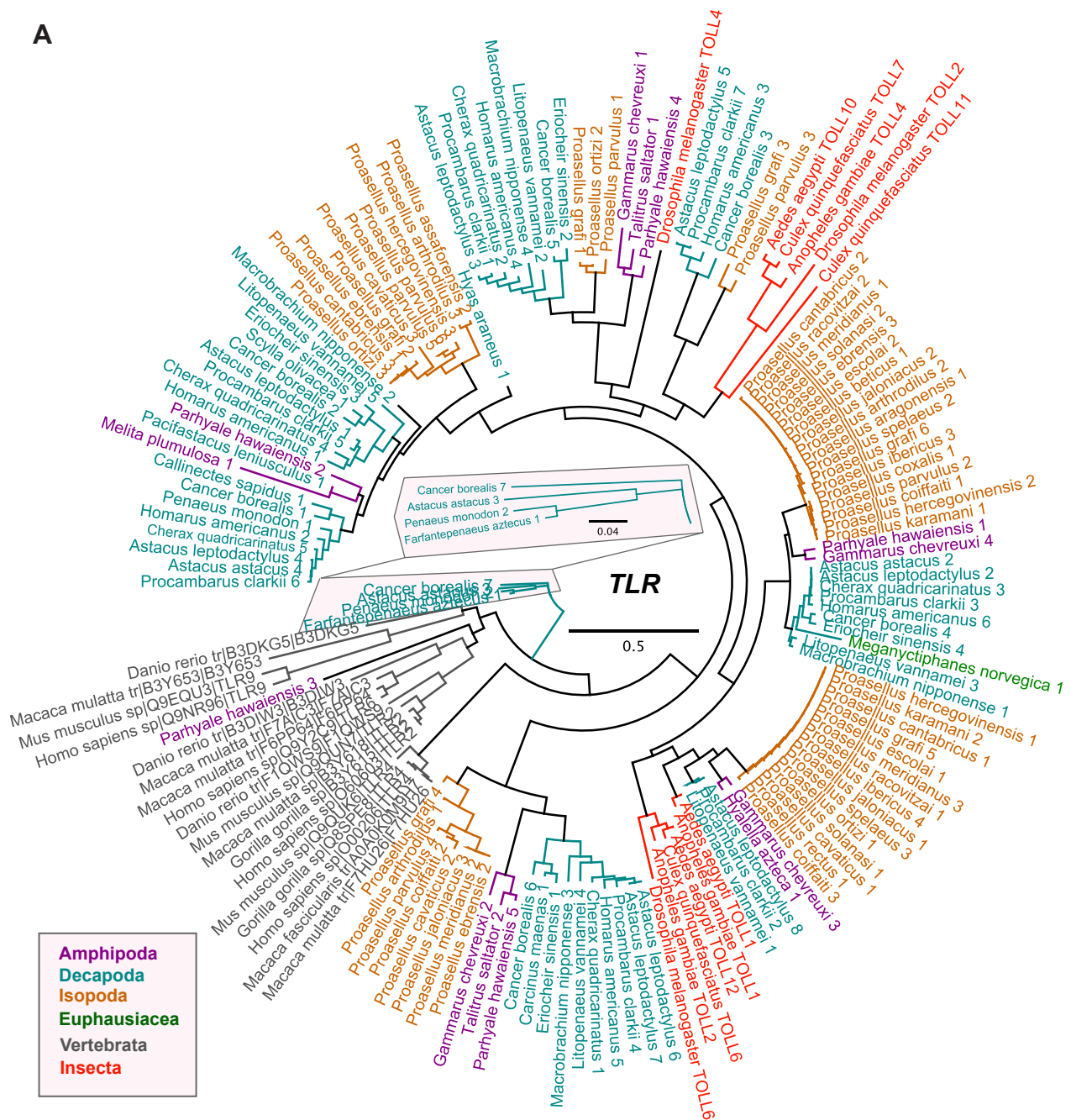

B

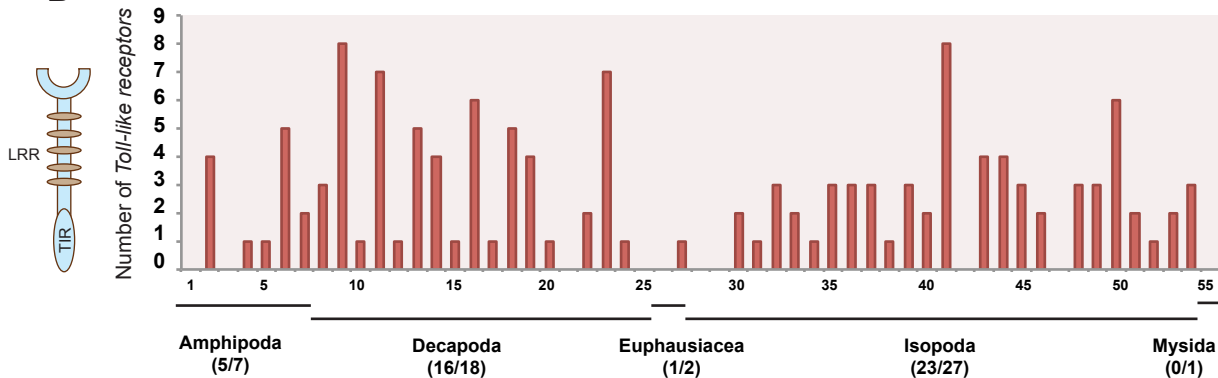

Supplement: Supplementary file 8 — Malacostracans Toll-like receptors (TLRs). (A) Phylogenetic tree of TLRs is constructed using the maximum-likelihood method from an amino acid multiple sequence alignment of toll-IL-1 receptor (TIR) domains. Taxa labels are depicted as their respective colour codes. Bootstrap support values (n=1000) for all trees can be found in Additional file 14: Figure S8. Scale bar represents substitution per site. (B) Graph of putative TLR transcripts. The y-axes represent total number of genes identified in all 55 malacostracan species for each family. Each species is represented by a number on the X-axes and a complete list of species is available in Additional file 3: Table S2. Black horizontal bars below each graph delimit the five orders of malacostracans and the numbers in parentheses (x/y) represent the following: x = number of species in which a particular gene family is found and y = total number of species in each order. (PDF 1124 kb) [file 12864_2017_3769_MOESM8_ESM.pdf]

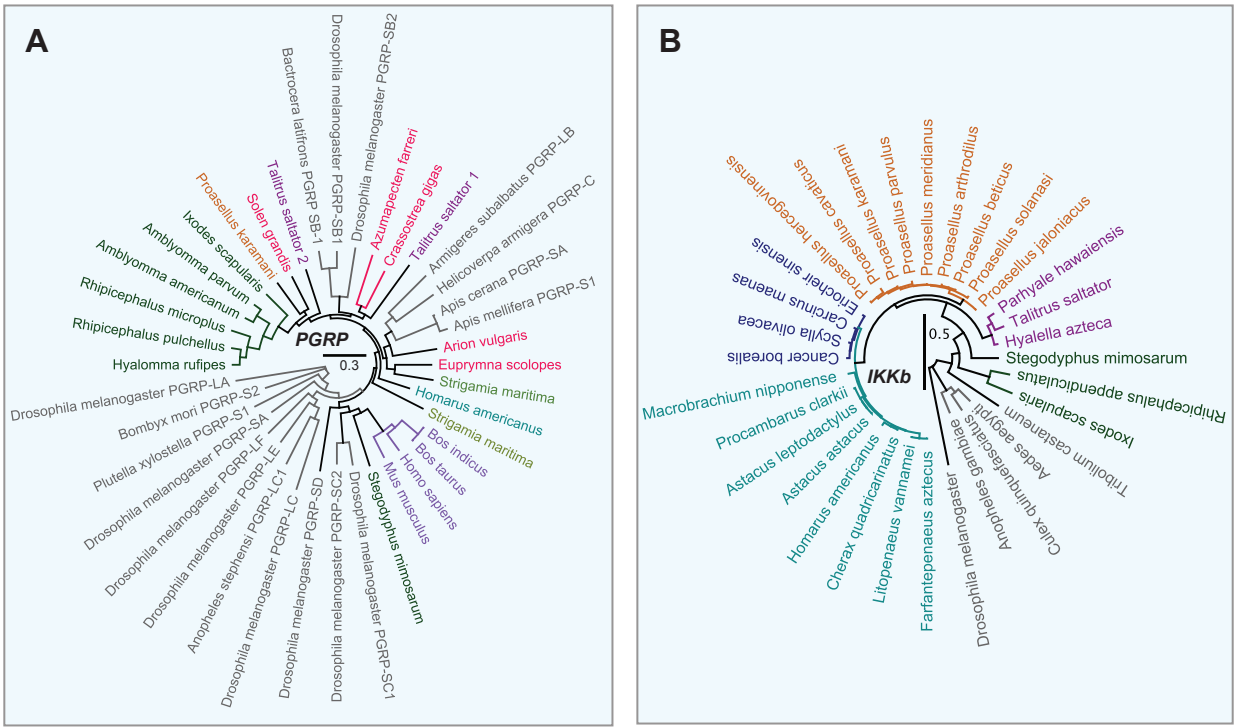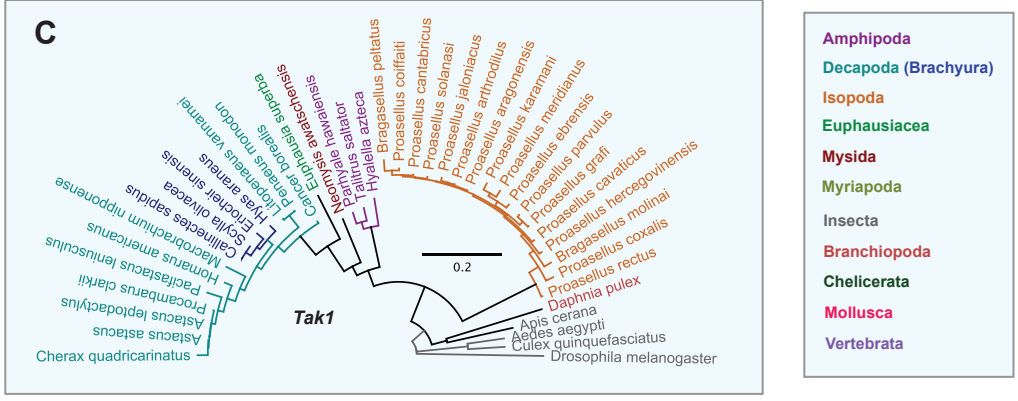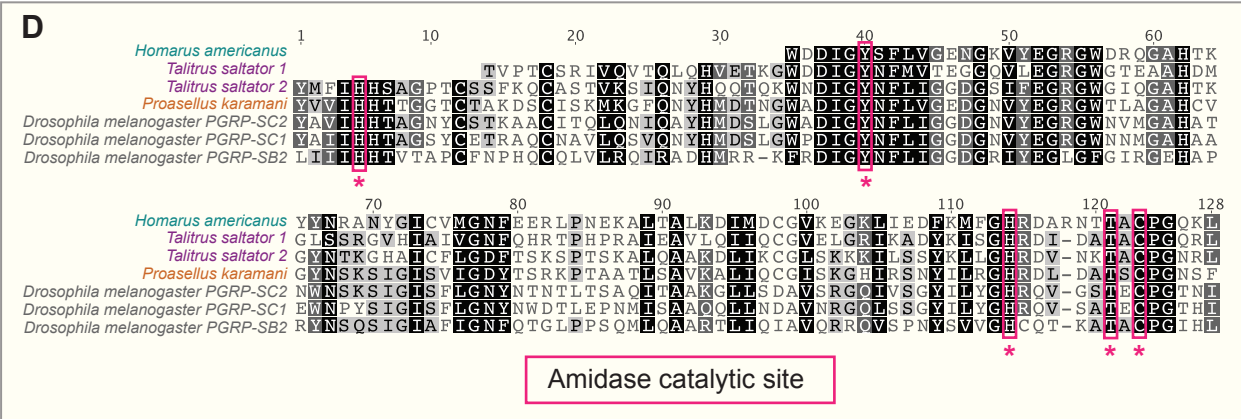

Supplement: Supplementary file 10 — Additional members of the IMD pathway. Phylogenetic trees of (A) PGRP, (B) IκB kinase β (IKKb); also known as immune response deficient-5 (IRD-5) and (C) TAK1 are constructed using the maximum-likelihood method from an amino acid multiple sequence alignment. Taxa labels are depicted as their respective colour codes. Bootstrap support values (n=1000) for all trees can be found in Additional file 14: Figure S8. Scale bar represents substitution per site. (D) Multiple sequence alignment of four PGRPs identified in malacostracans. Conserved amidase catalytic residues are highlighted in red boxes. (PDF 1151 kb) [file 12864_2017_3769_MOESM10_ESM.pdf]

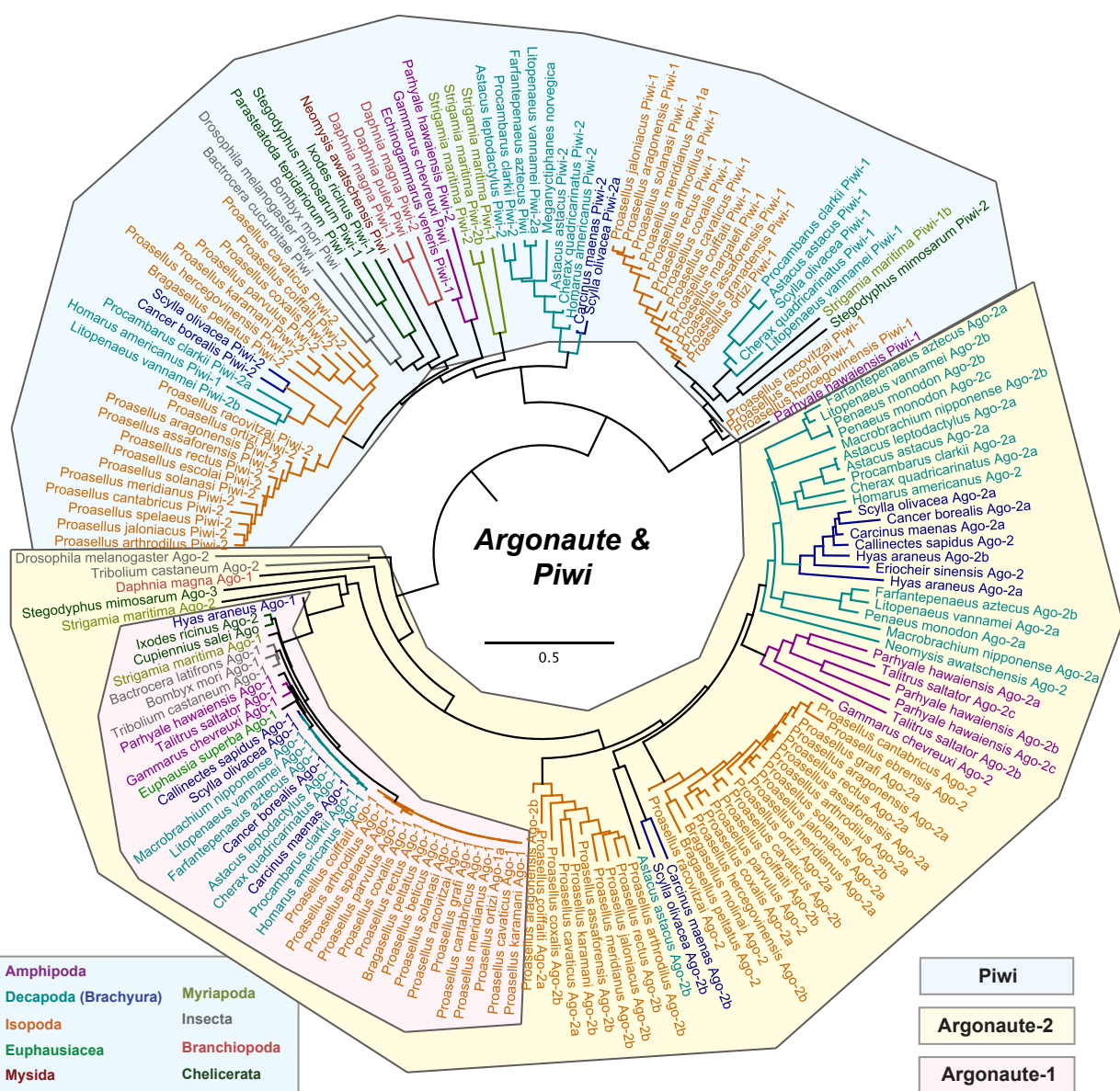

Supplement: Supplementary file 17 — Argonaute and Piwi gene families in malacostracans. Phylogenetic tree of the Argonaute and Piwi family is constructed using the maximum-likelihood method from an amino acid multiple sequence alignment. Seven main groups of SOCS proteins are identified in malacostracans. Taxa labels are depicted as their respective colour codes. Bootstrap support values (n=1000) for all trees can be found in Additional file 14: Figure S8. Scale bar represents substitution per site. (PDF 1315 kb) [file 12864_2017_3769_MOESM17_ESM.pdf]

# A

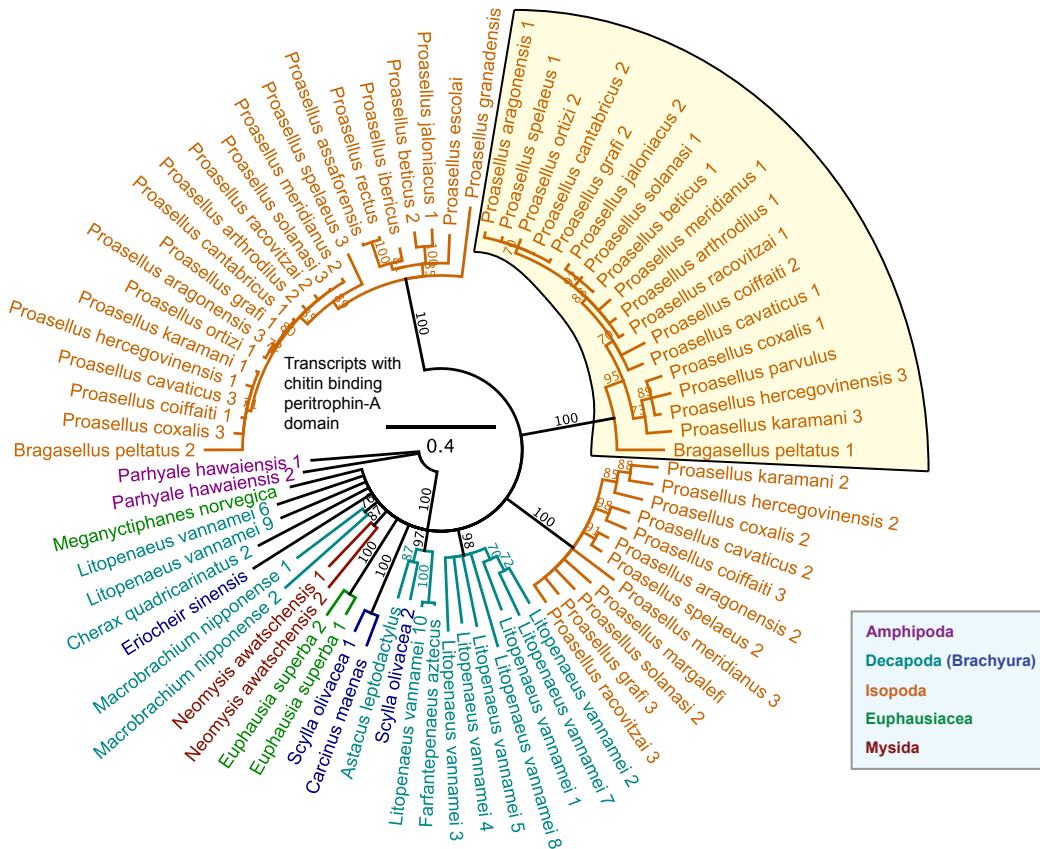

# B

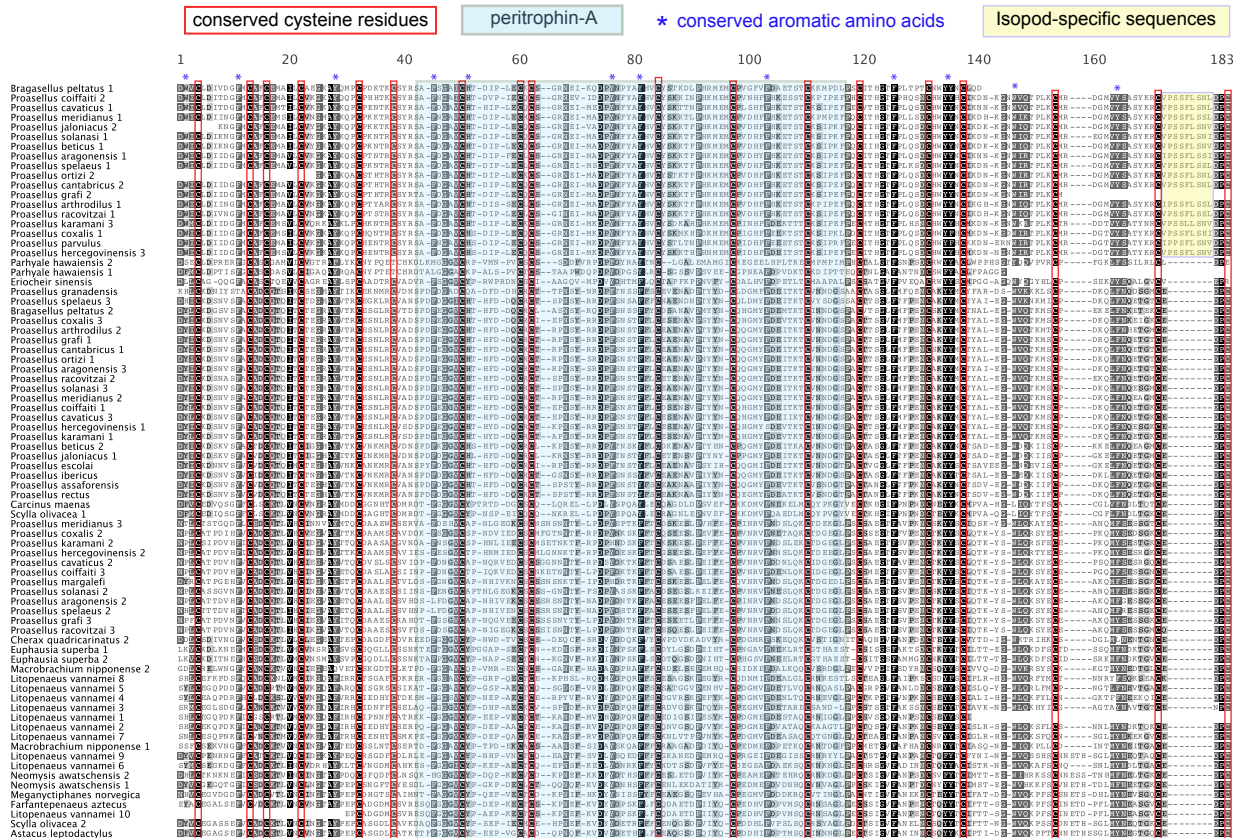

Supplement: Supplementary file 20 — Novel gene family with potential innate immunity function characterised by the chitin binding peritrophin-A domain (pfam01607) found only in malacostracans. (A) Phylogenetic tree of proteins containing the chitin binding peritrophin-A domain is constructed using the maximum-likelihood method from an amino acid multiple sequence alignment. Taxa labels are depicted as their respective colour codes. Node labels represent bootstrap support values from 1000 replicates. Scale bar represents substitution per site. (B) Multiple sequence alignment of peritrophin-A proteins showing the conserved peritrophin-A domain marked with a blue box, conserved cysteine residues marked in red boxes and conserved aromatic amino acids indicated by blue asterisks. The yellow shaded box represents a group of proteins with additional isopod-specific sequences. (PDF 5816 kb) [file 12864_2017_3769_MOESM20_ESM.pdf]

A

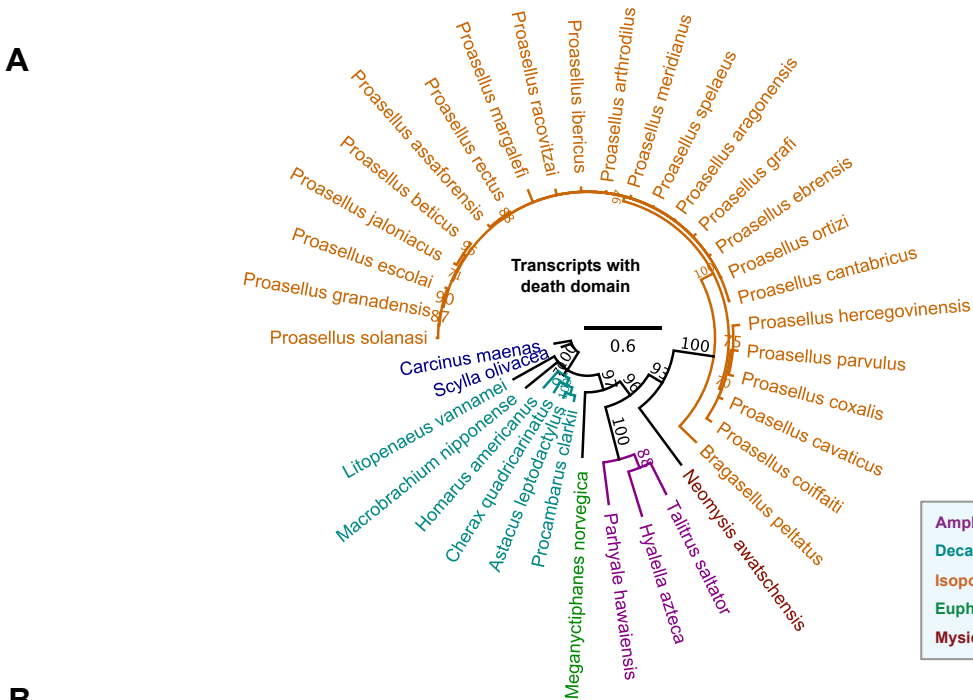

B

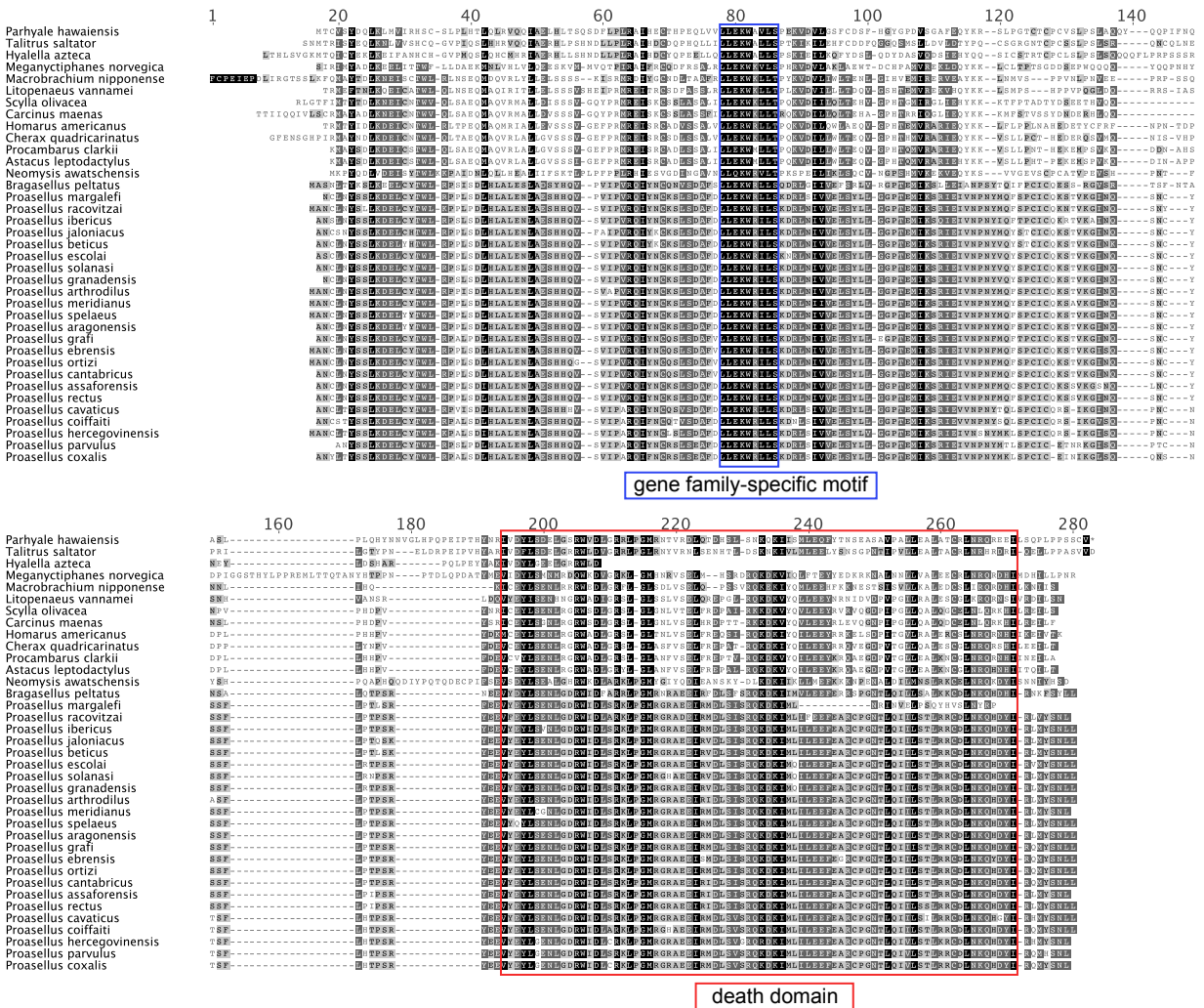

Supplement: Supplementary file 21 — Novel gene family with potential innate immunity function characterised by the death domain (pfam00531) found only in malacostracans. (A) Phylogenetic tree of proteins containing the death domain is constructed using the maximum-likelihood method from an amino acid multiple sequence alignment. Taxa labels are depicted as their respective colour codes. Node labels represent bootstrap support values from 1000 replicates. Scale bar represents substitution per site. (B) Multiple sequence alignment of the death domain family showing the death domain marked with a red box and a novel gene-family-specific motif marked with a blue box. (PDF 3603 kb) [file 12864_2017_3769_MOESM21_ESM.pdf]

A

Amphipoda  
Decapoda (Brachyura)  
Isopoda  
Euphausiacea  
Mysida

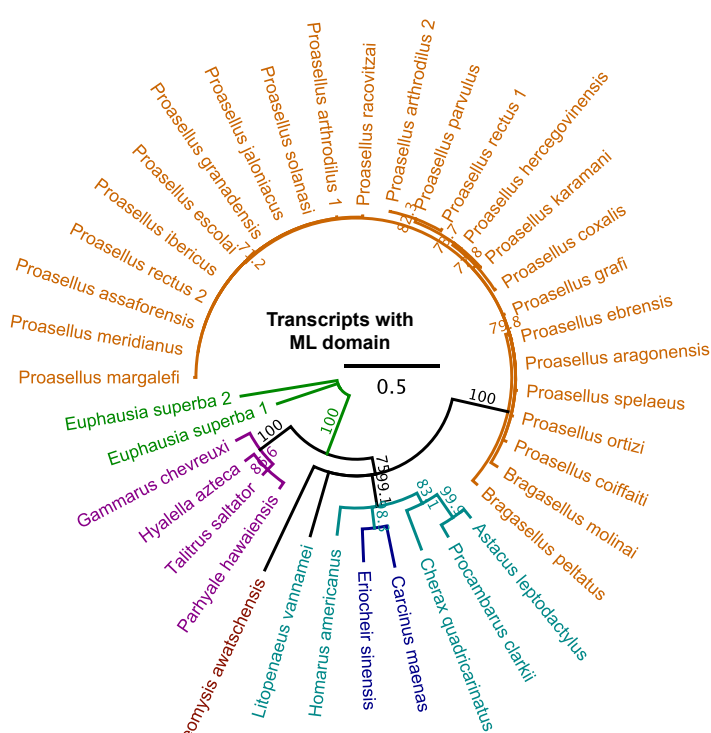

B

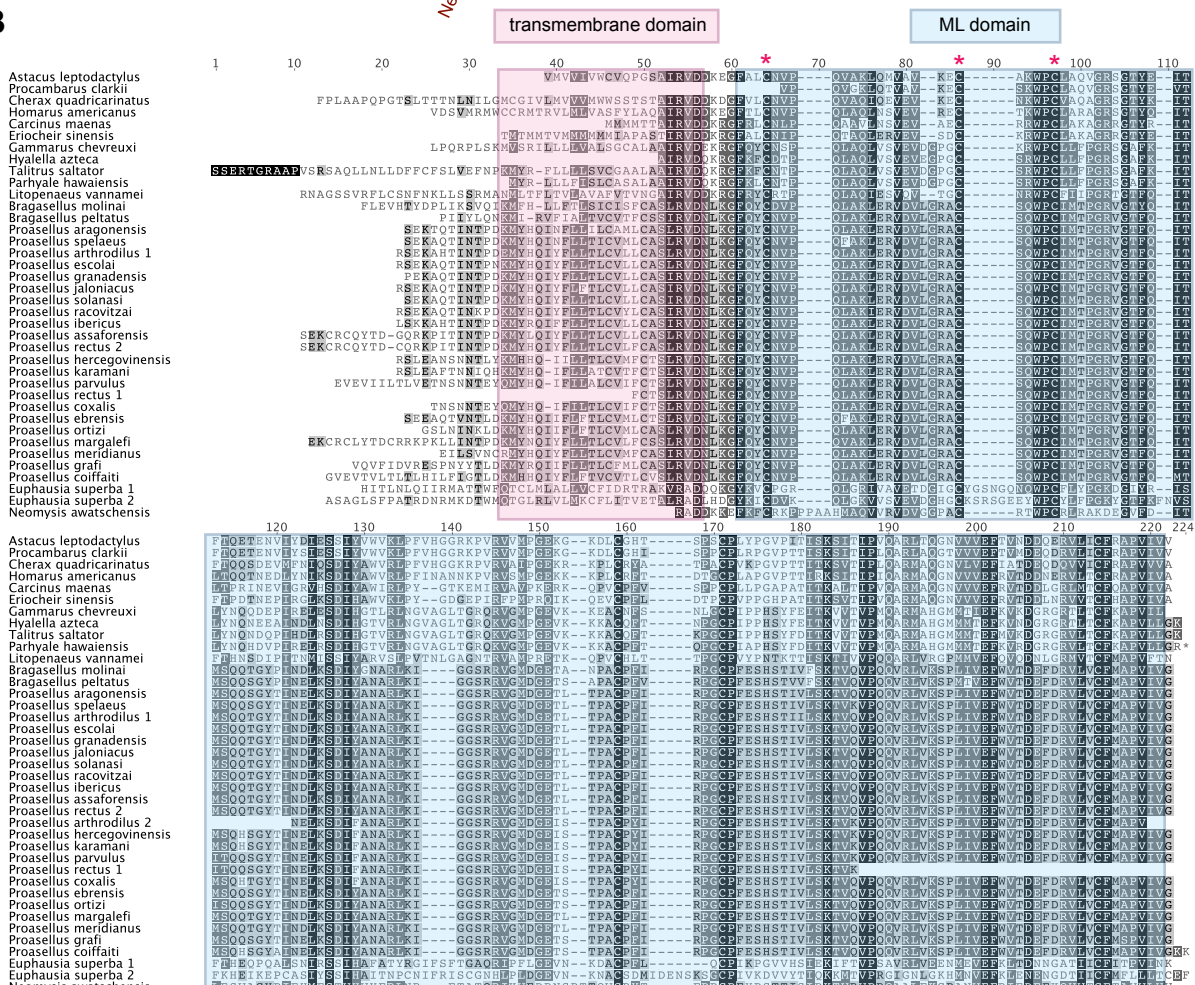

Supplement: Supplementary file 22 — Novel gene family with potential innate immunity function characterised by the ML domain (pfam02221) found only in malacostracans. (A) Phylogenetic tree of proteins containing the ML domain is constructed using the maximum-likelihood method from an amino acid multiple sequence alignment. Taxa labels are depicted as their respective colour codes. Node labels represent bootstrap support values from 1000 replicates. Scale bar represents substitution per site. (B) Multiple sequence alignment of the ML family showing the ML domain characterised by six conserved cysteine residues marked with red asterisks. Transmembrane domains were predicted using the Phobius program (Käll et al., [254]; Käll et al., [255]) and are annotated with a pink box. (PDF 3188 kb) [file 12864_2017_3769_MOESM22_ESM.pdf]

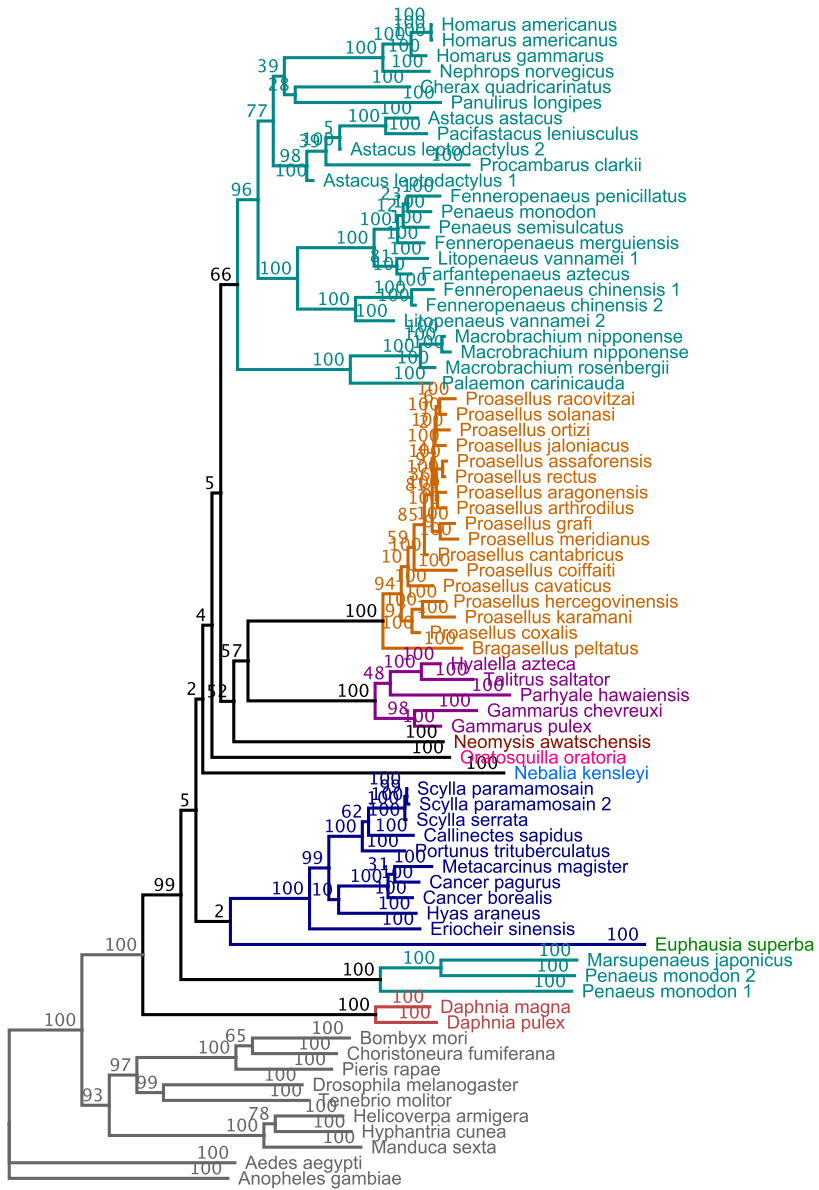

0.2

## Prophenoloxidasases

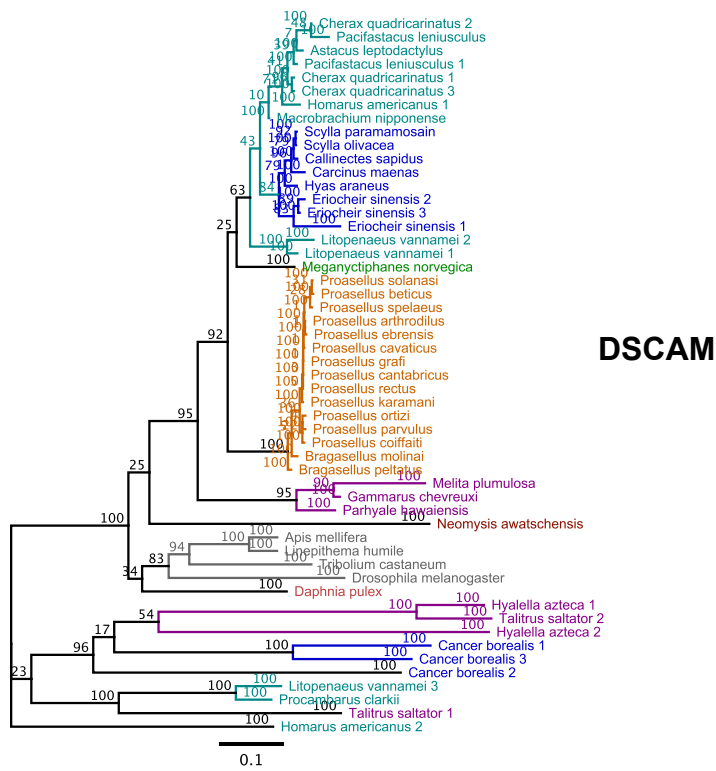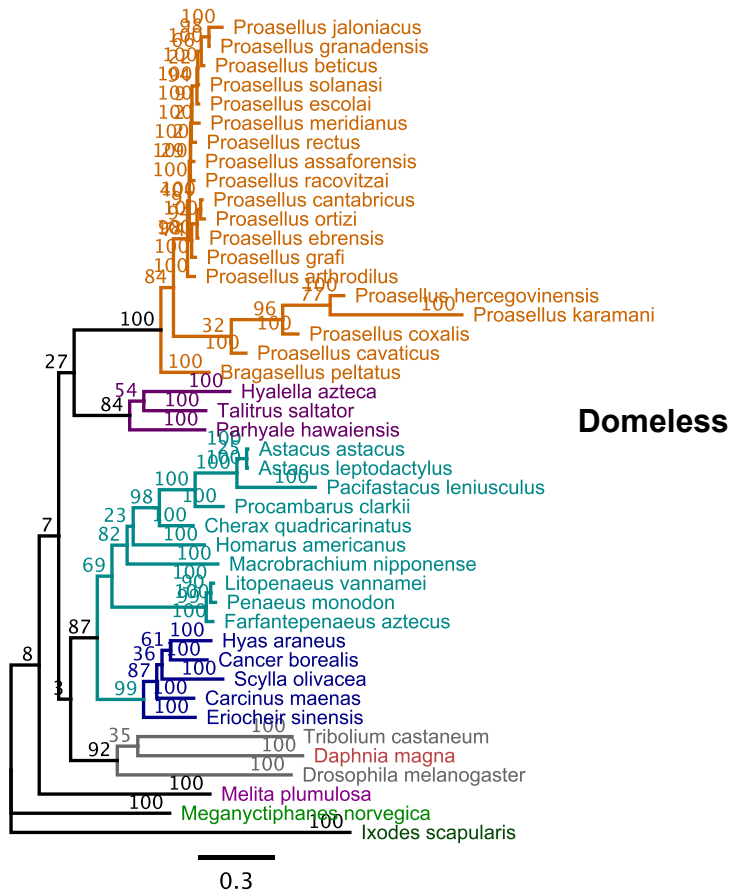

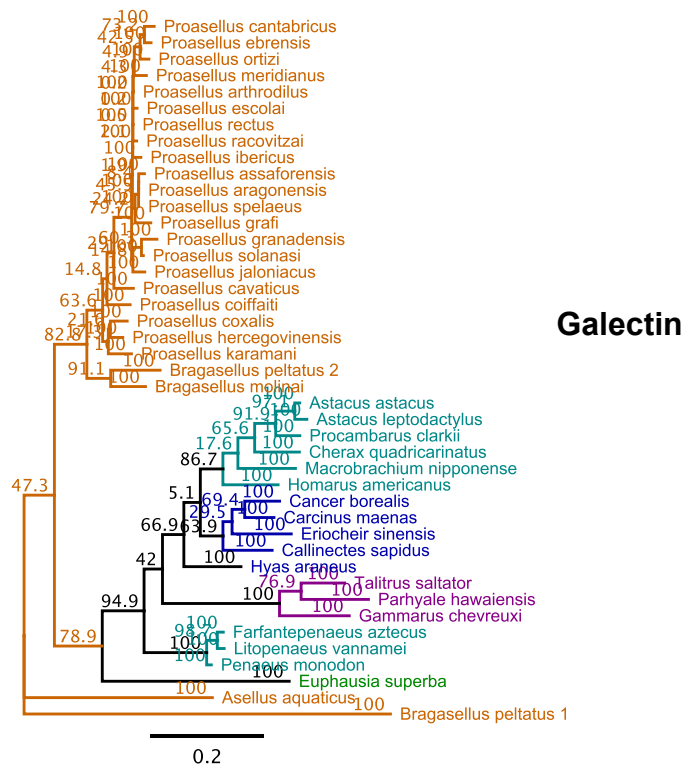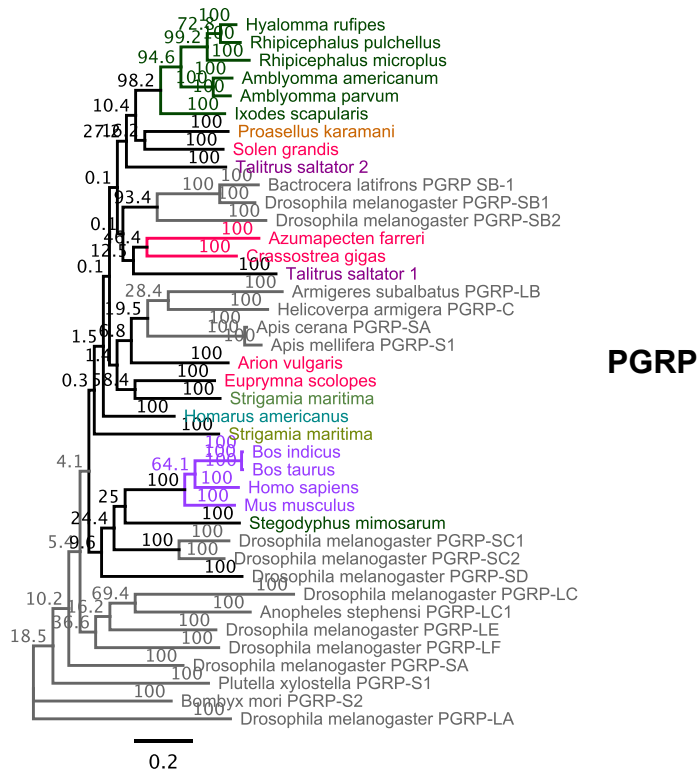

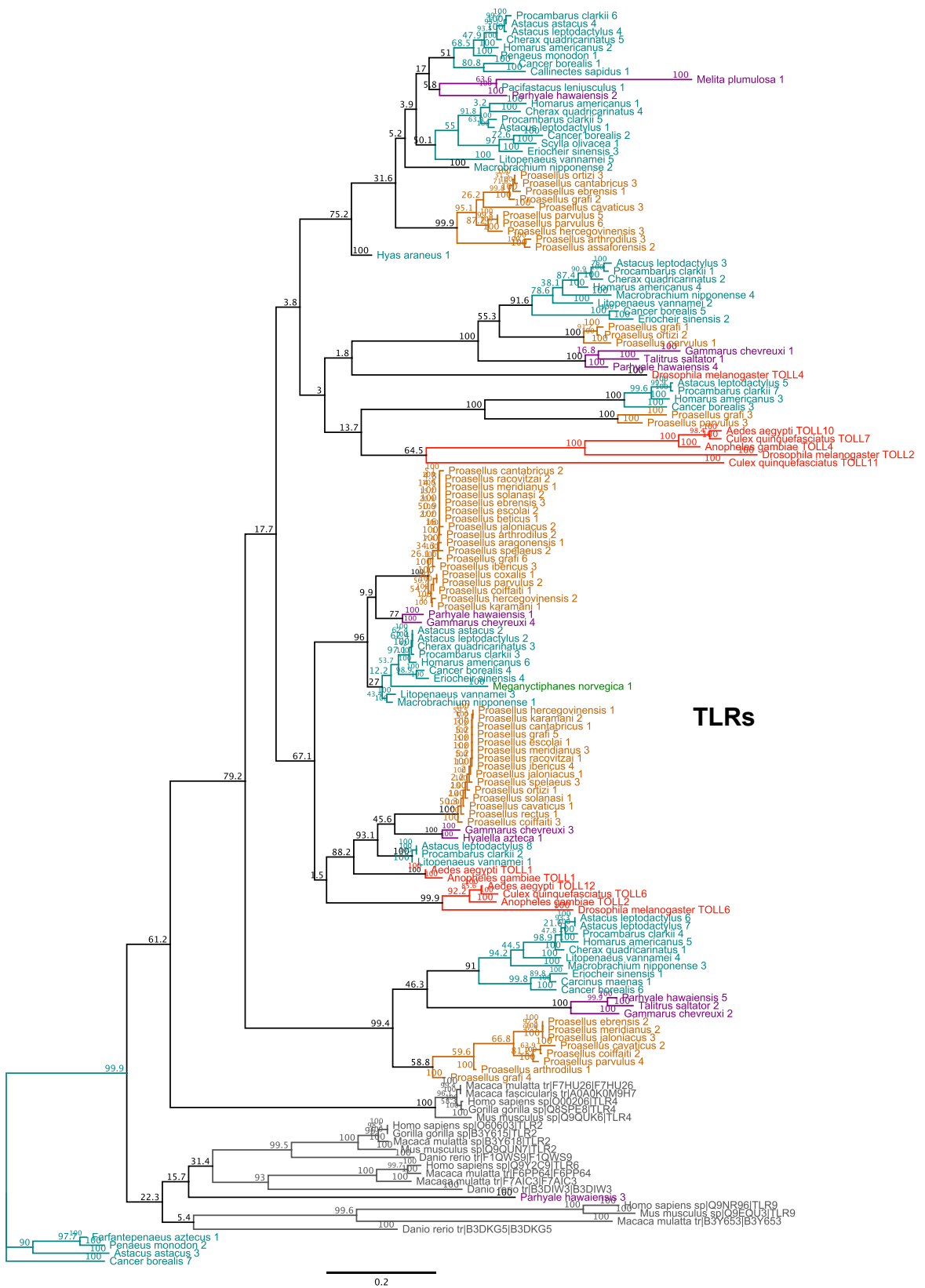

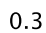

## TEPs

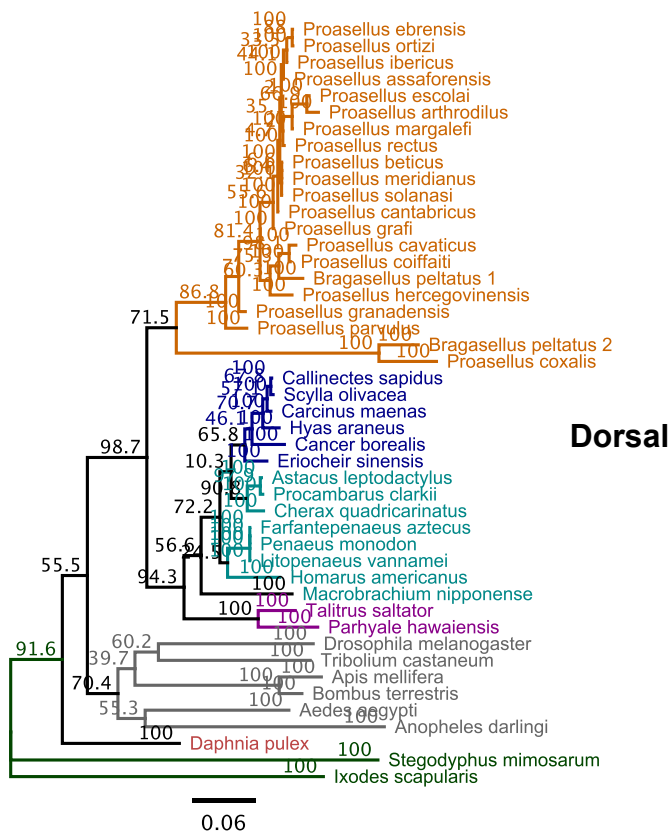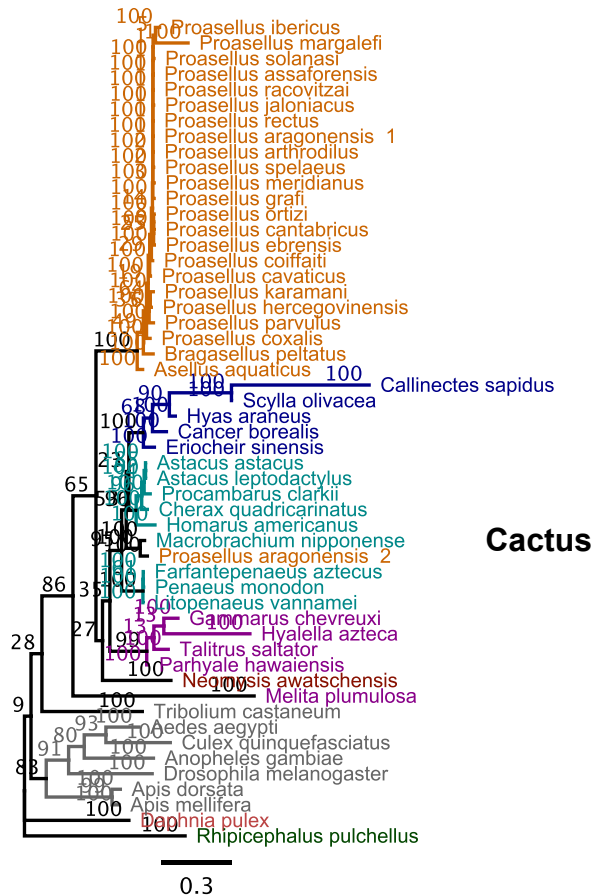

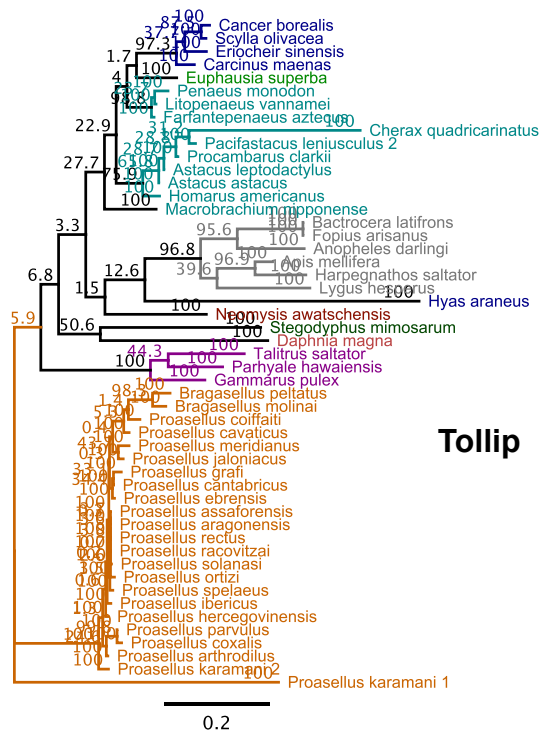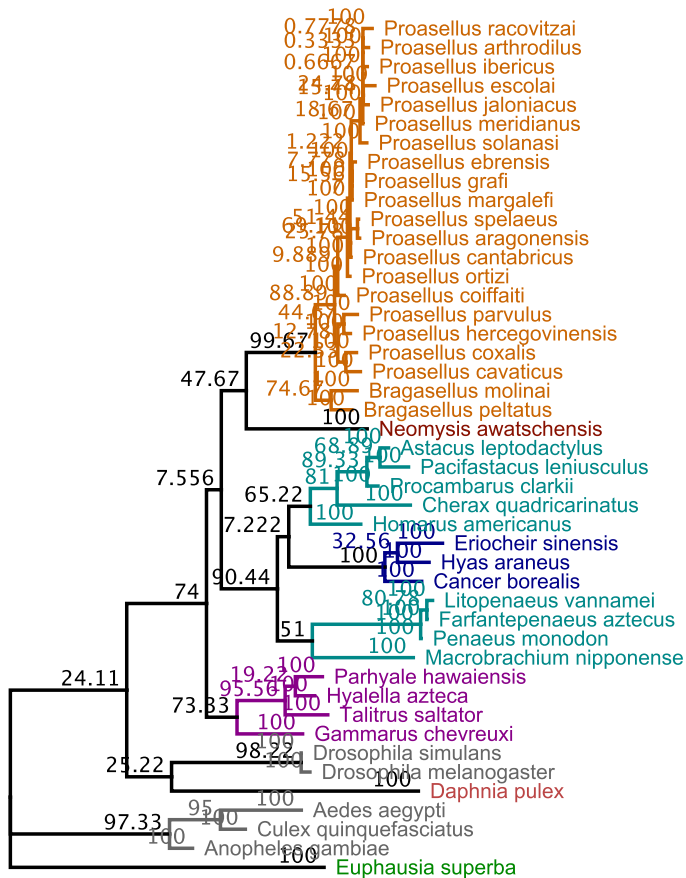



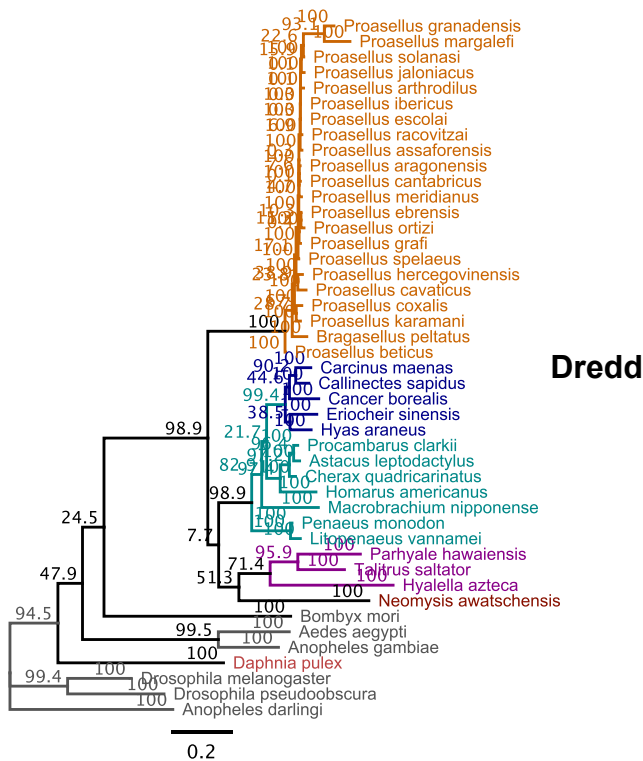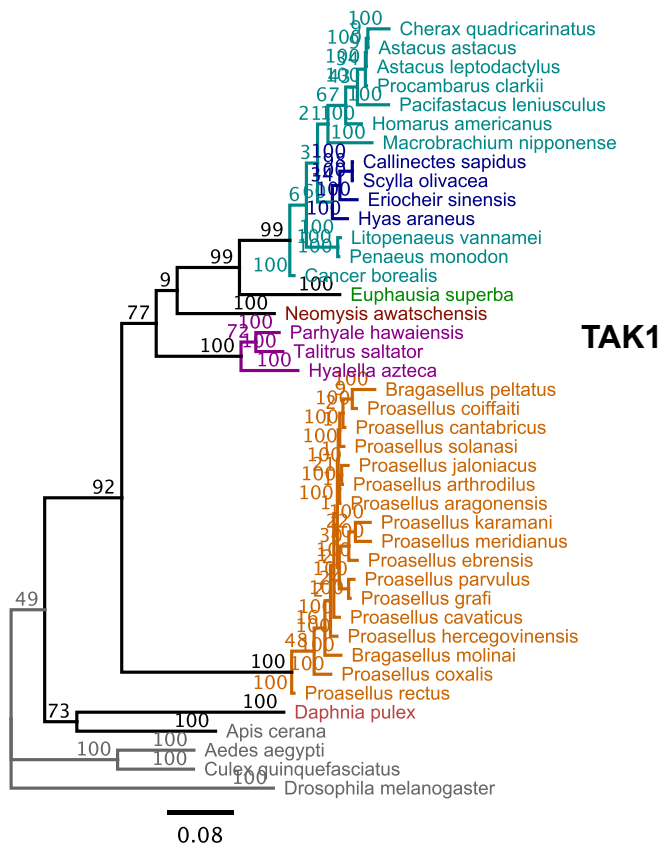

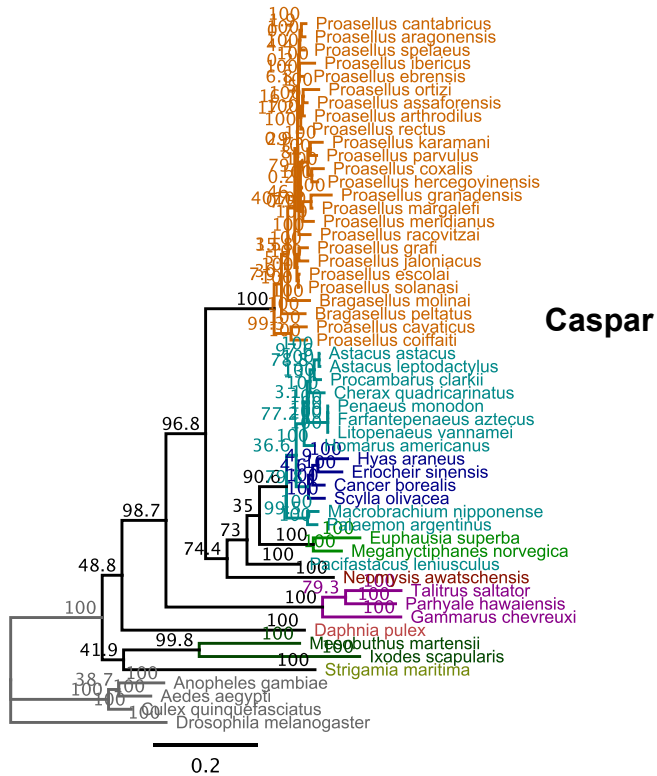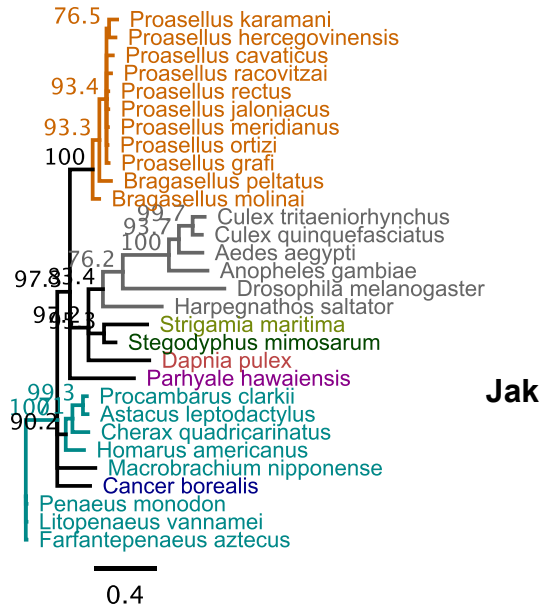

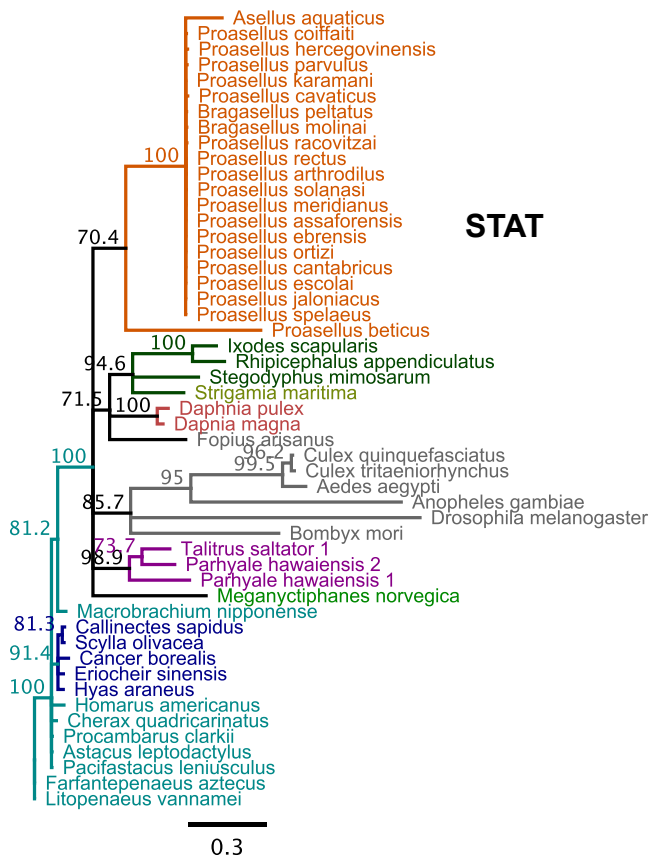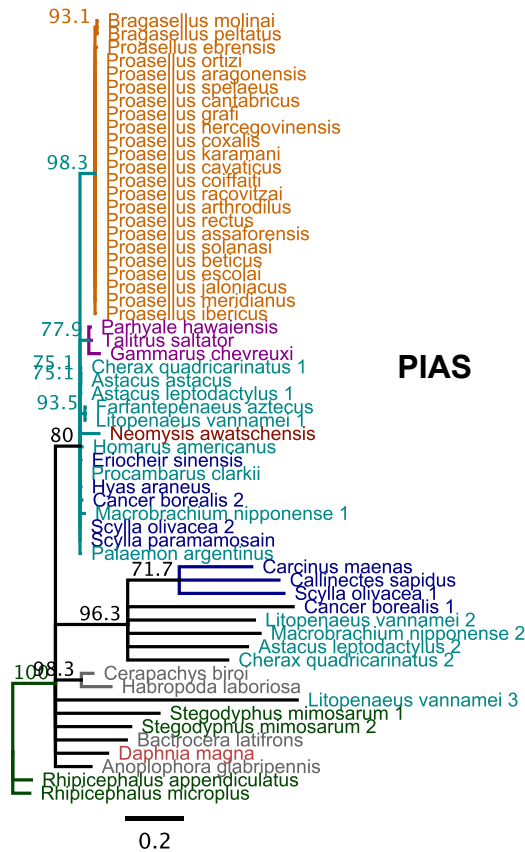

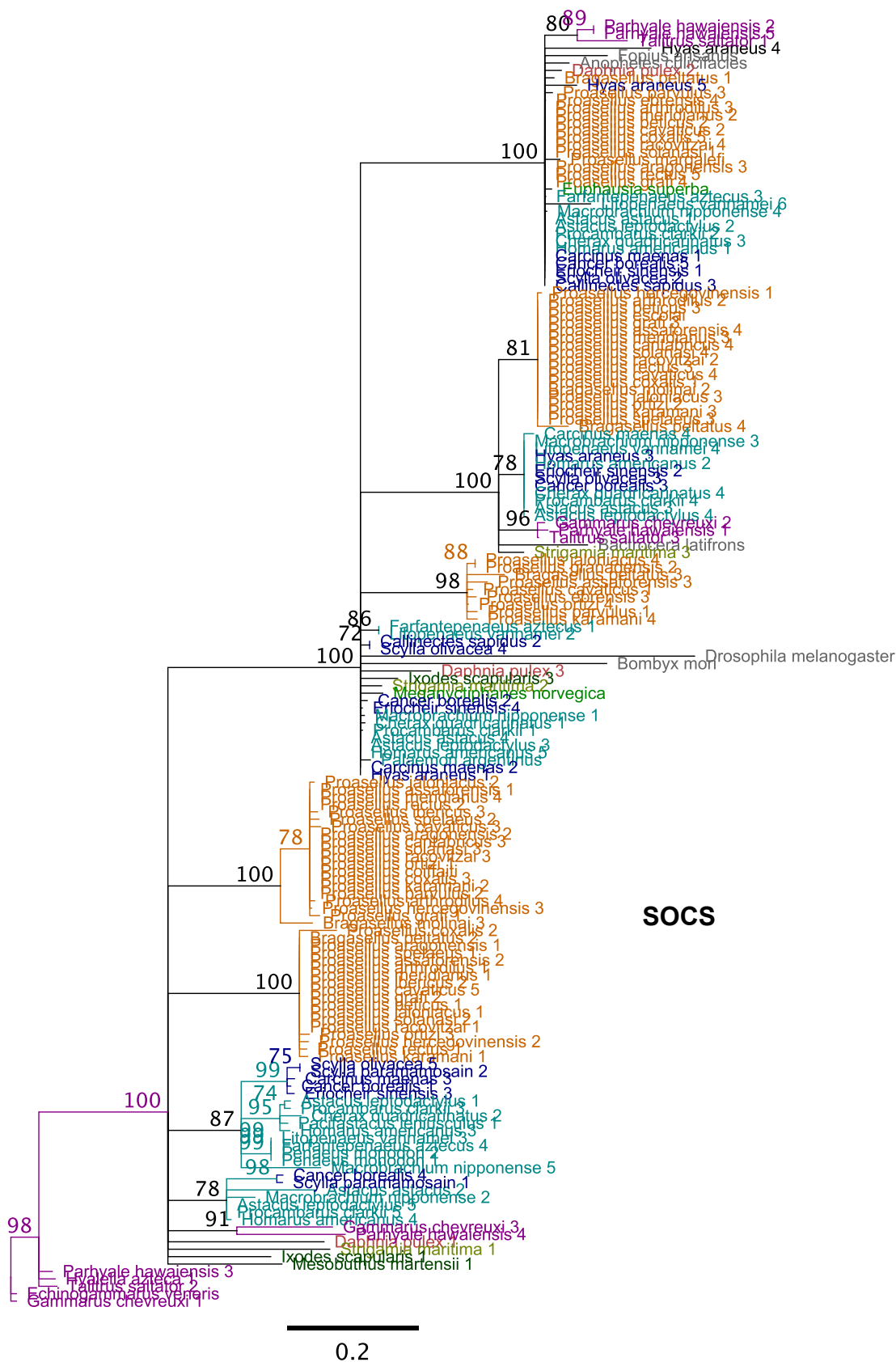

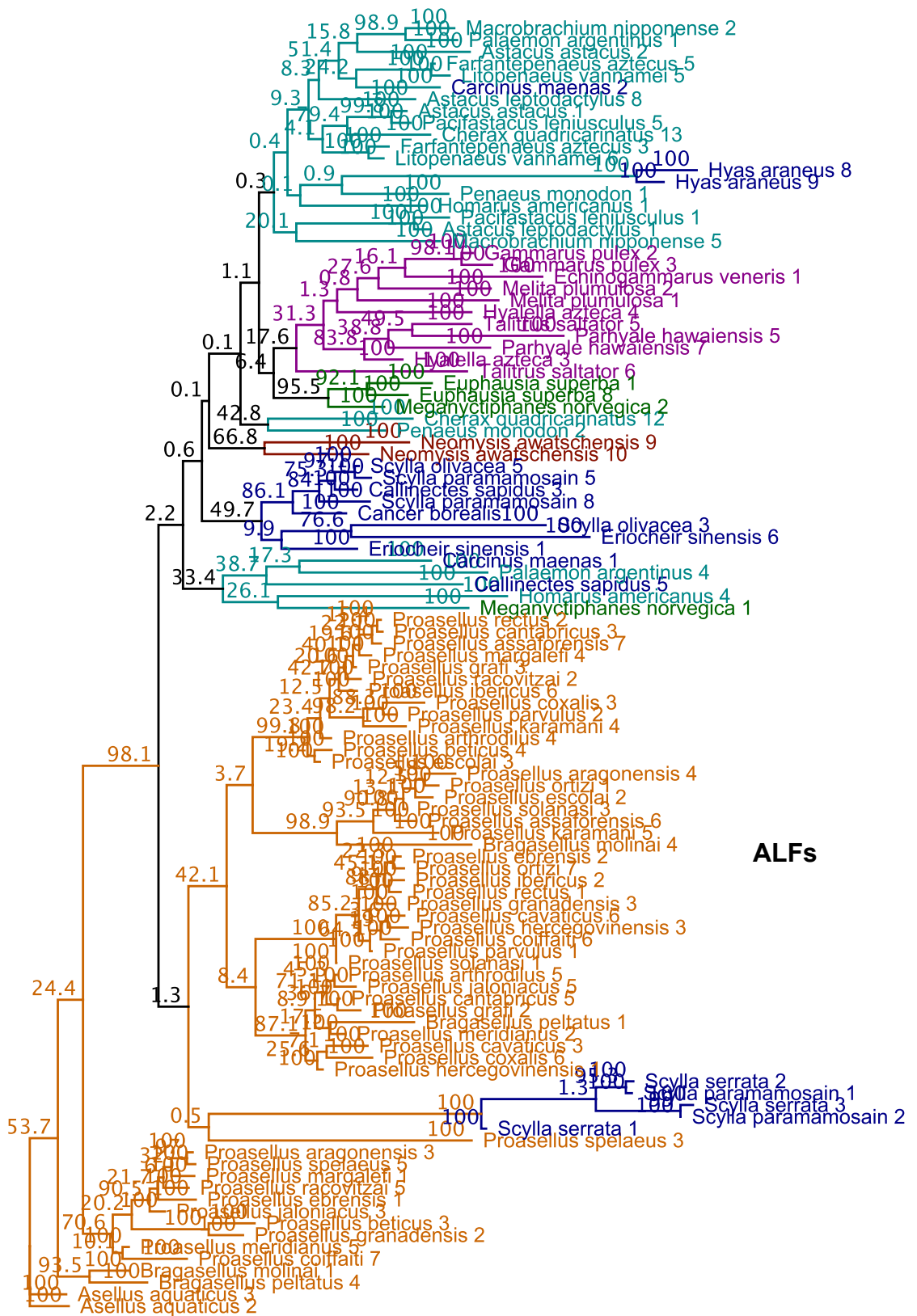

0.3

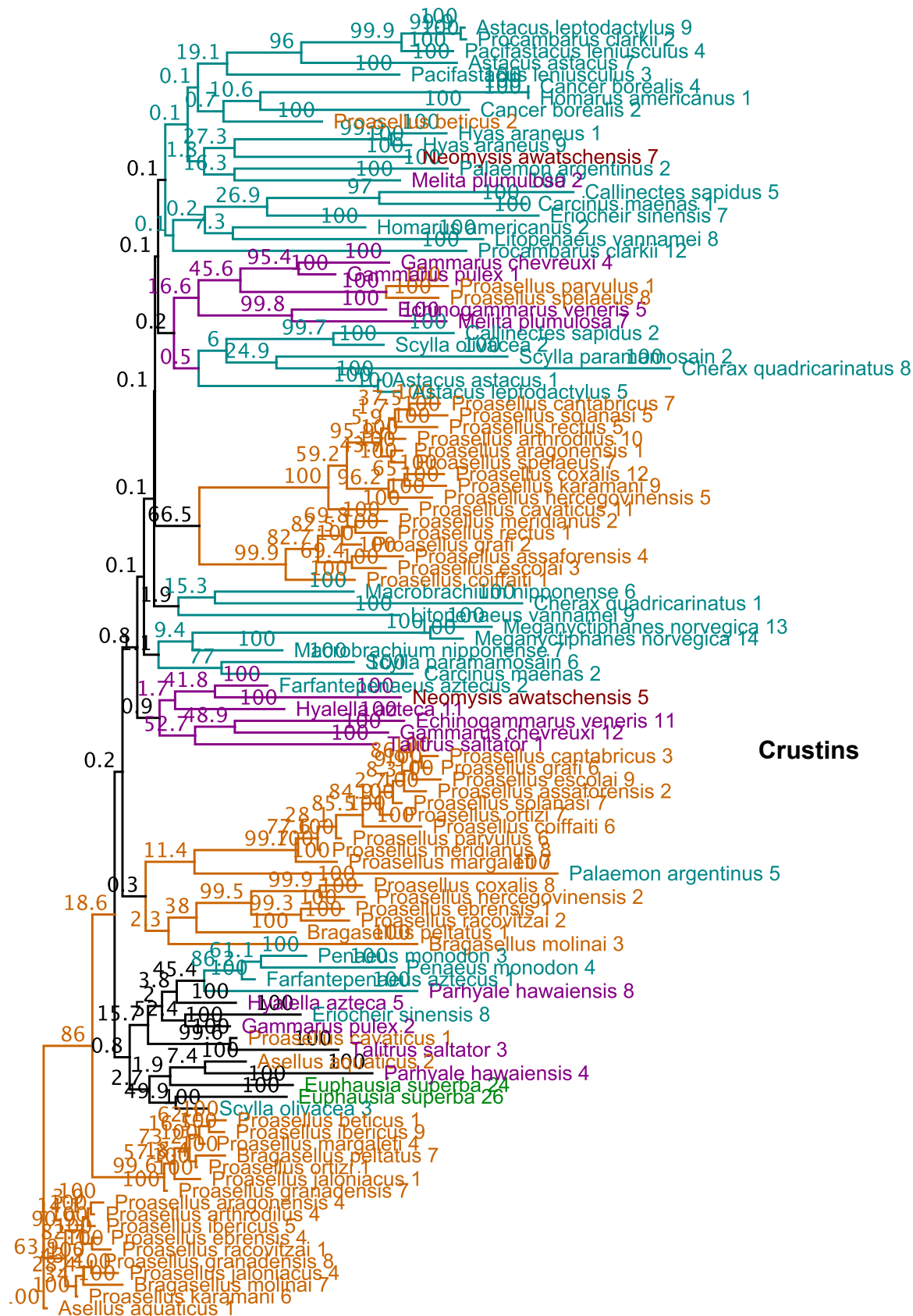

Crustins

0.2

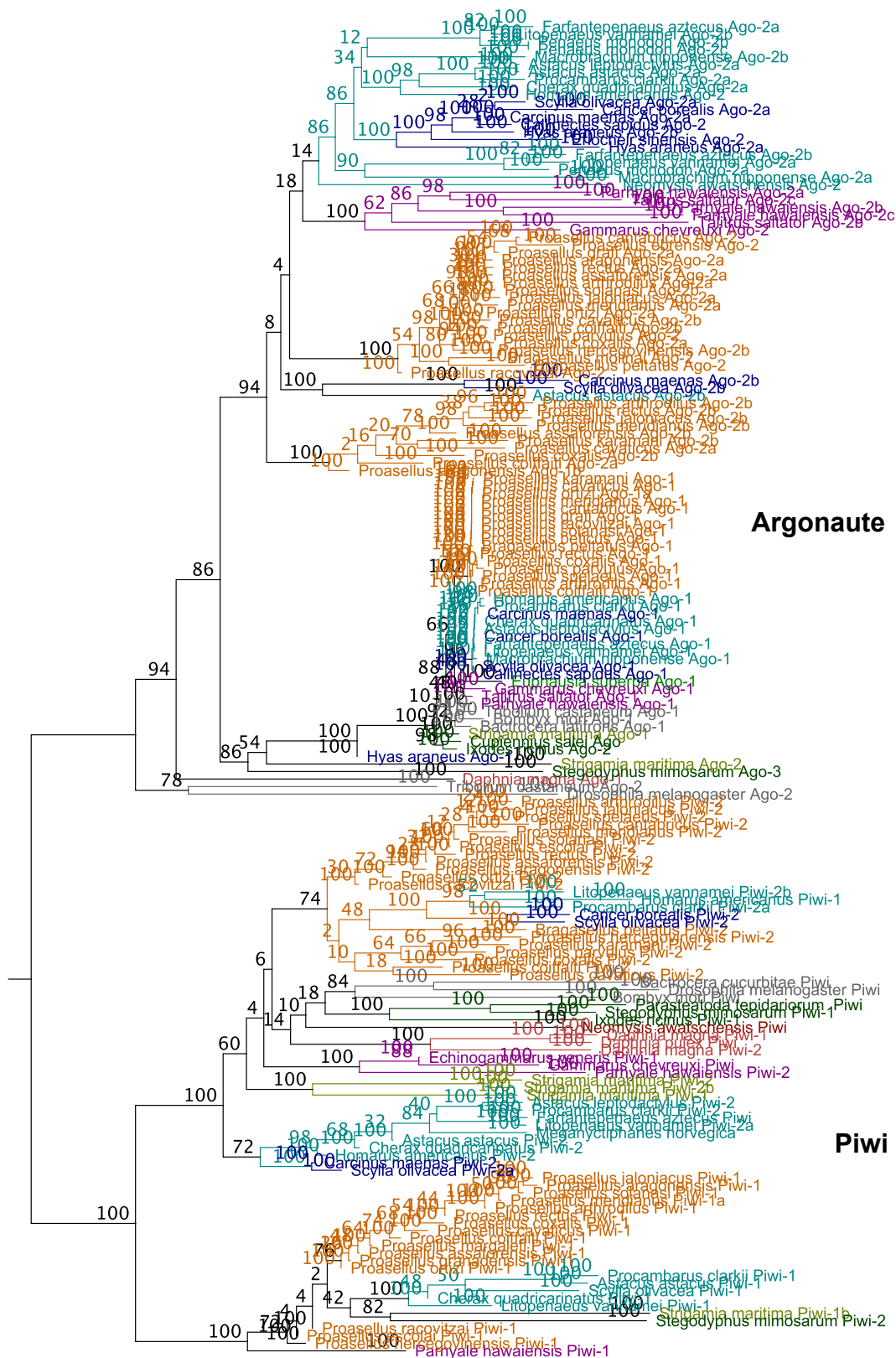

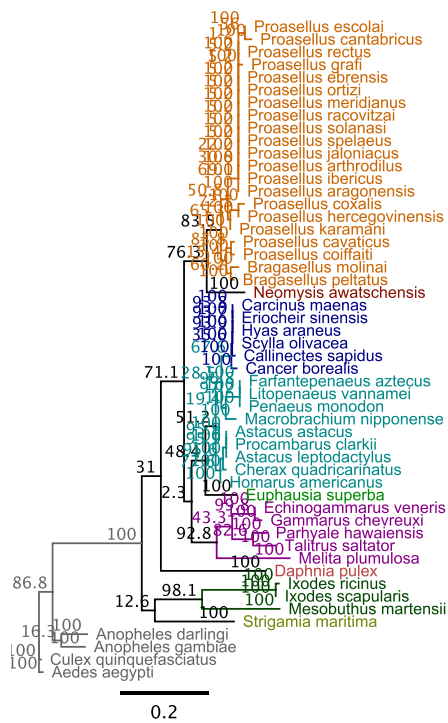

TRBP

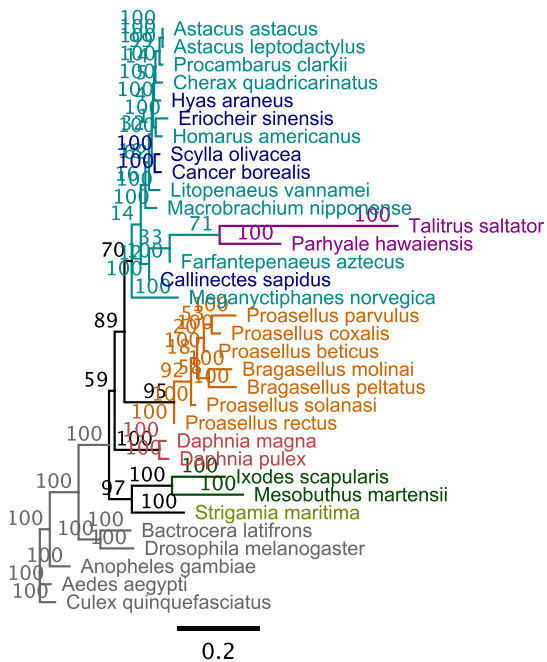

Drosha

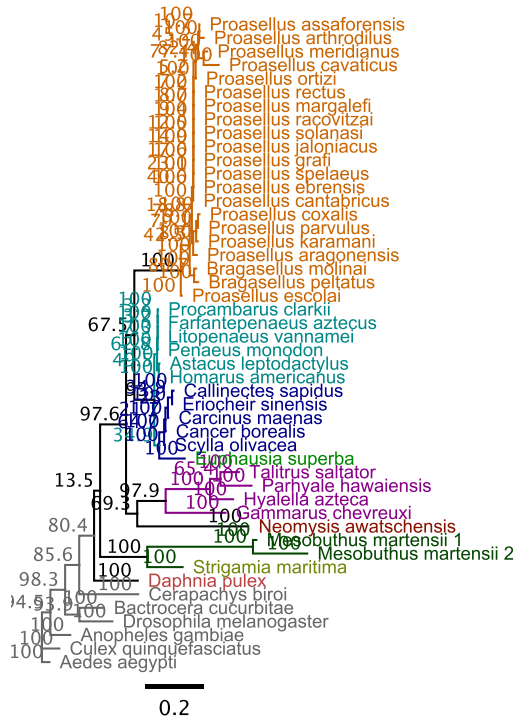

Pasha

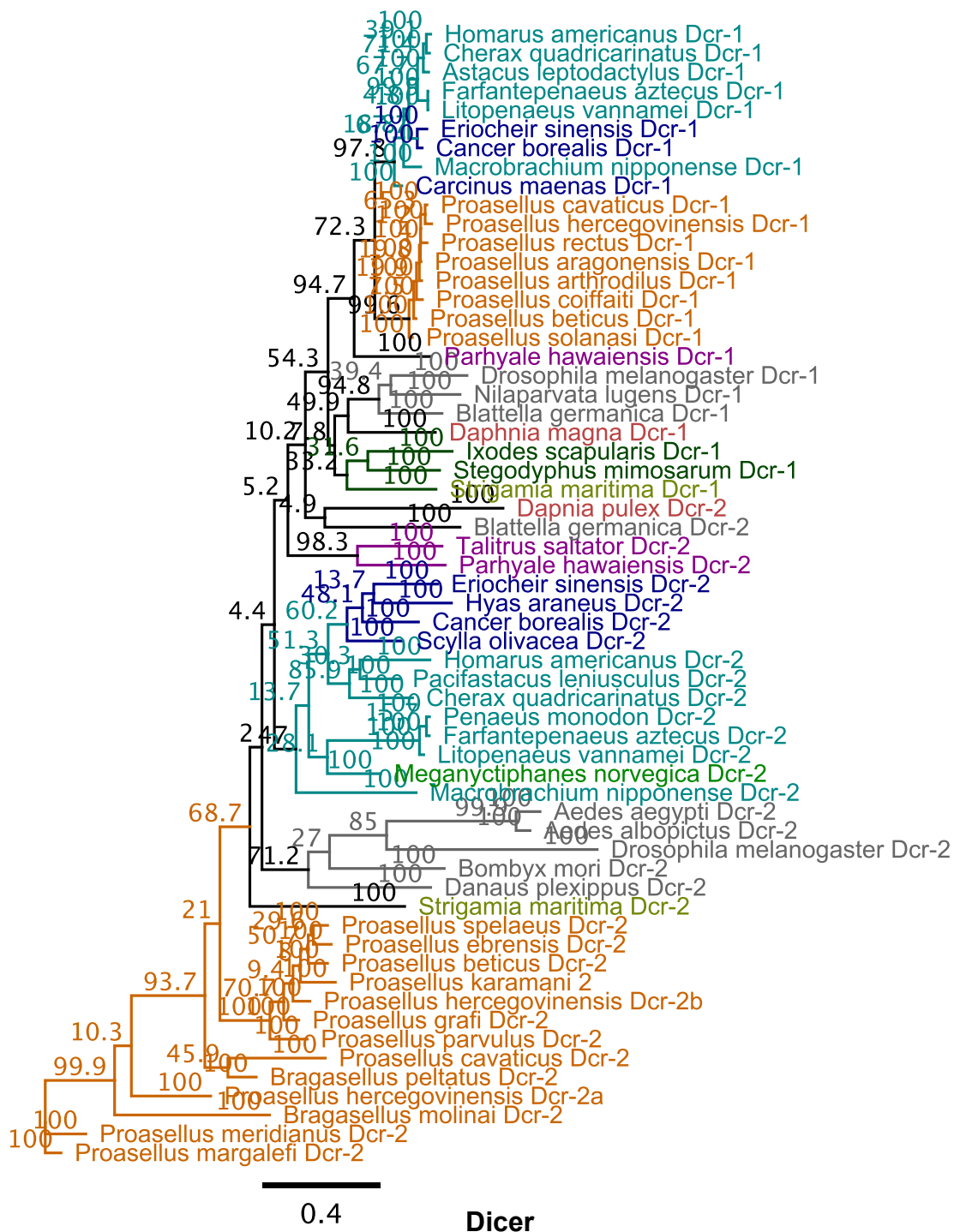

Supplement: Supplementary file 25 — Phylogenetic trees presented in this study with node labels representing bootstrap support values from 1000 replicates. Scale bars represent substitution per site. (PDF 11640 kb) [file 12864_2017_3769_MOESM26_ESM.pdf]
