# Supplementary material for: Comparative genomic analysis of innate immunity reveals novel and conserved components in crustacean food crop species
Source: BMC Genomics. 2017 May 18;18:389. doi: 10.1186/s12864-017-3769-4 (PMC5437397; doi:10.1186/s12864-017-3769-4)
Supplement: Supplementary file 9 — Malacostracans Toll pathway components. (PDF 474 kb) [file 12864_2017_3769_MOESM9_ESM.pdf]

Additional file 4. Malacostracans Toll pathway components.

Additional file 4A. Toll-like receptors

Arthropoda

Class (subphylum)	Species	Tissue type	Total gene counts	References
Insecta	Drosophila melanogaster	whole organism	9	McTaggart et al., 2009
Insecta	Anopheles gambiae	whole organism	10	McTaggart et al., 2009
Insecta	Aedes aegypti	whole organism	12	McTaggart et al., 2009
Chilopoda (Myriapoda)	Strigamia maritima	whole organism	27	Palmer et al., 2015
Arachnida (Chelicerata)	Mesobuthus martensii	whole organism	14	Palmer et al., 2015
Arachnida (Chelicerata)	Ixodes scapularis	whole organism	4	Palmer et al., 2015
Branchiopoda (Crustacea)	Daphnia pulex	whole organism	5	Palmer et al., 2015

Malacostraca

Order	Species/Datasets	Tissue type	Total gene counts with both LRR and TIR motifs
Amphipoda	Echinogammarus veneris	NA	0
Amphipoda	Gammarus chevreuxi	NA	4
Amphipoda	Gammarus pulex	NA	0
Amphipoda	Hyalella azteca	NA	1
Amphipoda	Melita plumulosa	whole organism	1
Amphipoda	Parhyale hawaiensis	whole organism	5
Amphipoda	Talitrus saltator	brain	2
Decapoda	Astacus astacus	hepatopancreas, ovaries, green glands, abdominal musculature	3
Decapoda	Astacus leptodactylus	hypodermis, Y organ, hepatopancreas, gills, hematocytes, muscle	8
Decapoda	Callinectes sapidus	gill 7	1
Decapoda	Cancer borealis	nervous system	7
Decapoda	Carcinus maenas	NA	1
Decapoda	Cherax quadricarinatus	hypodermis and gastrolith disc, heart, kidney, liver, nerve, testis	5
Decapoda	Eriocheir sinensis	eyestalk, Y-organ, and hepatopancreas	4
Decapoda	Farfantepenaeus aztecus	hepatopancreas	1
Decapoda	Homarus americanus	nervous system	6
Decapoda	Hyas araneus	adult	1
Decapoda	Litopenaeus vannamei	Ghaffari et al., 2014	5
Decapoda	Macrobrachium nipponense	NA	4
Decapoda	Pacifastacus leniusculus	Brain, HPT, Hemocyte, Hepatopancreas	1
Decapoda	Palaemon argentinus	whole organism	0
Decapoda	Penaeus monodon	hepatopancreas	2
Decapoda	Procambarus clarkii	Eyestalk, brain, hemocytes, gills, testis, ovary, hepatopancreas, heart, green gland, ventralganglia, Y-organ, hypodermis, muscle	7
Decapoda	Scylla olivacea	Na	1
Decapoda	Scylla paramamosain	gill	0
Euphausiacea	Euphausia superba	NA	0
Euphausiacea	Meganyctiphanes norvegica	adult	1
Isopoda	Asellus aquaticus	NA	0
Isopoda	Bragasellus molinai	whole organism	0
Isopoda	Bragasellus peltatus	whole organism	2
Isopoda	Proasellus aragonensis	whole organism	1
Isopoda	Proasellus arthrodilus	whole organism	3
Isopoda	Proasellus assaforensis	whole organism	2
Isopoda	Proasellus beticus	whole organism	1
Isopoda	Proasellus cantabricus	whole organism	3
Isopoda	Proasellus cavaticus	whole organism	3
Isopoda	Proasellus coiffaiti	whole organism	3
Isopoda	Proasellus coxalis	whole organism	1
Isopoda	Proasellus ebrensis	whole organism	3
Isopoda	Proasellus escolai	whole organism	2
Isopoda	Proasellus grafi	whole organism	8
Isopoda	Proasellus granadensis	whole organism	0
Isopoda	Proasellus hercegovinensis	whole organism	4
Isopoda	Proasellus ibericus	whole organism	4
Isopoda	Proasellus jaloniacus	whole organism	3
Isopoda	Proasellus karamani	whole organism	2
Isopoda	Proasellus margalefi	whole organism	0
Isopoda	Proasellus meridianus	whole organism	3
Isopoda	Proasellus ortizi	whole organism	3

Isopoda	Proasellus parvulus	whole organism	6
Isopoda	Proasellus racovitzai	whole organism	2
Isopoda	Proasellus rectus	whole organism	1
Isopoda	Proasellus solanasi	whole organism	2
Isopoda	Proasellus spelaeus	whole organism	3
Mysida	Neomysis awatschensis	whole organism	0
		**Total malacostracan genes**	136

Additional file 4B. Tube.

Arthropoda

Class (subphylum)	Species	Tissue type	Total gene counts	References
Insecta	Drosophila melanogaster	whole organism	1	Palmer et al., 2015
Insecta	Anopheles gambiae	whole organism	1	McTaggart et al., 2009
Insecta	Aedes aegypti	whole organism	1	McTaggart et al., 2009
Chilopoda (Myriapoda)	Strigamia maritima	whole organism	0	Palmer et al., 2015
Arachnida (Chelicerata)	Mesobuthus martensii	whole organism	1	Palmer et al., 2015
Arachnida (Chelicerata)	Ixodes scapularis	whole organism	0	Palmer et al., 2015
Branchiopoda (Crustacea)	Daphnia pulex	whole organism	0	McTaggart et al., 2009

Malacostraca

Order	Species/Datasets	Tissue type	Tube	Total number of non-redundant genes per species
Amphipoda	Echinogammarus veneris	NA	0	0
Amphipoda	Gammarus chevreuxi	NA	0	0
Amphipoda	Gammarus pulex	NA	0	0
Amphipoda	Hyalella azteca_1	NA	0	
Amphipoda	Hyalella azteca_2	NA	0	
Amphipoda	Hyalella azteca_3	whole organism	0	0
Amphipoda	Melita plumulosa	whole organism	0	0
Amphipoda	Parhyale hawaiensis	whole organism	0	0
Amphipoda	Talitrus saltator	brain	0	0
Decapoda	Astacus astacus	hepatopancreas, ovaries, green gla	1	1
Decapoda	Astacus leptodactylus_1	hypodermis; Y organ	1	
Decapoda	Astacus leptodactylus_2	hepatopancreas	1	
Decapoda	Astacus leptodactylus_3	hypodermis, Y organ, hepatopancre	1	1
Decapoda	Callinectes sapidus	gill 7	0	0
Decapoda	Cancer borealis	nervous system	1	1
Decapoda	Carcinus maenas	NA	0	0
Decapoda	Cherax quadricarinatus_1	hypodermis and gastrolith disc	0	
Decapoda	Cherax quadricarinatus_2	heart, kidney, liver, nerve, testis	0	
Decapoda	Cherax quadricarinatus_3	heart, kidney, liver, nerve, testis	0	0
Decapoda	Eriocheir sinensis_1	NA	1	
Decapoda	Eriocheir sinensis_2	eyestalk, Y-organ, and hepatopancr	1	
Decapoda	Eriocheir sinensis_3	hepatopancreas	0	1
Decapoda	Farfantepenaeus aztecus	hepatopancreas	1	1
Decapoda	Homarus americanus	nervous system	1	1
Decapoda	Hyas araneus_1	adult	0	
Decapoda	Hyas araneus_2	gill	1	1
Decapoda	Litopenaeus vannamei_1	Ghaffari et al., 2014	1	
Decapoda	Litopenaeus vannamei_2	hepatopancreas	1	
Decapoda	Litopenaeus vannamei_3	hepatopancreas	1	
Decapoda	Litopenaeus vannamei_4	hemocytes	1	1
Decapoda	Macrobrachium nipponense	NA	1	1
Decapoda	Pacifastacus leniusculus	Brain, HPT, Hemocyte, Hepatopanc	1	1
Decapoda	Palaemon argentinus	whole organism	0	0
Decapoda	Penaeus monodon_1	hepatopancreas	1	
Decapoda	Penaeus monodon_2	hepatopancreas	1	1
Decapoda	Procambarus clarkii_1	Eyestalk	1	
Decapoda	Procambarus clarkii_2	Eyestalk, brain, hemocytes, gills, te:	1	1
Decapoda	Scylla olivacea	Na	1	1
Decapoda	Scylla paramamosain	gill	1	1
Euphausiacea	Euphausia superba	NA	0	0
Euphausiacea	Meganyctiphanes norvegica	adult	0	0
Isopoda	Asellus aquaticus	NA	0	0
Isopoda	Bragasellus molinai	whole organism	1	1
Isopoda	Bragasellus peltatus	whole organism	1	1
Isopoda	Proasellus aragonensis	whole organism	0	0
Isopoda	Proasellus arthrodilus	whole organism	0	0
Isopoda	Proasellus assaforensis	whole organism	1	1
Isopoda	Proasellus beticus	whole organism	0	0
Isopoda	Proasellus cantabricus	whole organism	0	0
Isopoda	Proasellus cavaticus	whole organism	0	0
Isopoda	Proasellus coiffaiti	whole organism	0	0
Isopoda	Proasellus coxalis	whole organism	0	0
Isopoda	Proasellus ebrensis	whole organism	0	0
Isopoda	Proasellus escolai	whole organism	0	0
Isopoda	Proasellus grafi	whole organism	0	0
Isopoda	Proasellus granadensis	whole organism	0	0
Isopoda	Proasellus hercegovinensis	whole organism	0	0
Isopoda	Proasellus ibericus	whole organism	0	0
Isopoda	Proasellus jaloniacus	whole organism	0	0
Isopoda	Proasellus karamani	whole organism	0	0
Isopoda	Proasellus margalefi	whole organism	0	0

Isopoda	Proasellus meridianus	whole organism	0	0	
Isopoda	Proasellus ortizi	whole organism	0	0	
Isopoda	Proasellus parvulus	whole organism	0	0	
Isopoda	Proasellus racovitzai	whole organism	0	0	
Isopoda	Proasellus rectus	whole organism	0	0	
Isopoda	Proasellus solanasi	whole organism	0	0	
Isopoda	Proasellus spelaeus	whole organism	0	0	
Mysida	Neomysis awatschensis	whole organism	0	0	
			**Total malacostracan genes**		17

Additional file 4C. Pelle.

Arthropoda

Class (subphylum)	Species	Tissue type	Total gene counts	References
Insecta	Drosophila melanogaster	whole organism	1	Palmer et al., 2015
Insecta	Anopheles gambiae	whole organism	1	McTaggart et al., 2009
Insecta	Aedes aegypti	whole organism	1	McTaggart et al., 2009
Chilopoda (Myriapoda)	Strigamia maritima	whole organism	1	Palmer et al., 2015
Arachnida (Chelicerata)	Mesobuthus martensii	whole organism	0	Palmer et al., 2015
Arachnida (Chelicerata)	Ixodes scapularis	whole organism	2	Palmer et al., 2015
Branchiopoda (Crustacea)	Daphnia pulex	whole organism	2	McTaggart et al., 2009

Malacostraca

Order	Species/Datasets	Tissue type	Total gene counts	Total number of non-redundant genes per species
Amphipoda	Echinogammarus veneris	NA	2	2
Amphipoda	Gammarus chevreuxi	NA	1	1
Amphipoda	Gammarus pulex	NA	1	1
Amphipoda	Hyalella azteca_1	NA	1	
Amphipoda	Hyalella azteca_2	NA	1	
Amphipoda	Hyalella azteca_3	whole organism	1	1
Amphipoda	Melita plumulosa	whole organism	1	1
Amphipoda	Parhyale hawaiensis	whole organism	2	2
Amphipoda	Talitrus saltator	brain	2	2
Decapoda	Astacus astacus	hepatopancreas, ovaries, green glands, abdominal musculature	1	1
Decapoda	Astacus leptodactylus_1	hypodermis; Y organ	1	
Decapoda	Astacus leptodactylus_2	hepatopancreas	0	
Decapoda	Astacus leptodactylus_3	hypodermis, Y organ, hepatopancreas, gills, hematocytes, muscle	1	1
Decapoda	Callinectes sapidus	gill 7	1	1
Decapoda	Cancer borealis	nervous system	1	1
Decapoda	Carcinus maenas	NA	0	
Decapoda	Cherax quadricarinatus_1	hypodermis and gastrolith disc	0	
Decapoda	Cherax quadricarinatus_2	heart, kidney, liver, nerve, testis	1	
Decapoda	Cherax quadricarinatus_3	heart, kidney, liver, nerve, testis	1	1
Decapoda	Eriocheir sinensis_1	NA	1	
Decapoda	Eriocheir sinensis_2	eyestalk, Y-organ, and hepatopancreas	1	
Decapoda	Eriocheir sinensis_3	hepatopancreas	0	1
Decapoda	Farfantepenaeus aztecus	hepatopancreas	1	1
Decapoda	Homarus americanus	nervous system	2	2
Decapoda	Hyas araneus_1	adult	0	
Decapoda	Hyas araneus_2	gill	1	1
Decapoda	Litopenaeus vannamei_1	Ghaffari et al., 2014	2	
Decapoda	Litopenaeus vannamei_2	hepatopancreas	1	
Decapoda	Litopenaeus vannamei_3	hepatopancreas	1	
Decapoda	Litopenaeus vannamei_4	hemocytes	1	2
Decapoda	Macrobrachium nipponense	NA	1	1
Decapoda	Pacifastacus leniusculus	Brain, HPT, Hemocyte, Hepatopancreas	0	
Decapoda	Palaemon argentinus	whole organism	0	
Decapoda	Penaeus monodon_1	hepatopancreas	1	
Decapoda	Penaeus monodon_2	hepatopancreas	1	1
Decapoda	Procambarus clarkii_1	Eyestalk	1	
Decapoda	Procambarus clarkii_2	Eyestalk, brain, hemocytes, gills, testis, ovary, hepatopancreas, heart, green gland, ventralganglia, Y-organ, hypodermis, muscle	1	1
Decapoda	Scylla olivacea	Na	1	1
Decapoda	Scylla paramamosain	gill	uniprot	1
Euphausiacea	Euphausia superba	NA	1	1
Euphausiacea	Meganyctiphanes norvegica	adult	1	1
Isopoda	Asellus aquaticus	NA	0	
Isopoda	Bragasellus molinai	whole organism	1	1
Isopoda	Bragasellus peltatus	whole organism	1	1
Isopoda	Proasellus aragonensis	whole organism	1	1
Isopoda	Proasellus arthrodilus	whole organism	1	1
Isopoda	Proasellus assaforensis	whole organism	1	1
Isopoda	Proasellus beticus	whole organism	2	2
Isopoda	Proasellus cantabricus	whole organism	1	1
Isopoda	Proasellus cavaticus	whole organism	1	1
Isopoda	Proasellus coiffaiti	whole organism	2	2
Isopoda	Proasellus coxalis	whole organism	2	2

Isopoda	Proasellus ebrensis	whole organism	1	1
Isopoda	Proasellus escolai	whole organism	1	1
Isopoda	Proasellus grafi	whole organism	2	2
Isopoda	Proasellus granadensis	whole organism	1	1
Isopoda	Proasellus hercegovinensis	whole organism	2	2
Isopoda	Proasellus ibericus	whole organism	1	1
Isopoda	Proasellus jaloniacus	whole organism	1	1
Isopoda	Proasellus karamani	whole organism	1	1
Isopoda	Proasellus margalefi	whole organism	1	1
Isopoda	Proasellus meridianus	whole organism	1	1
Isopoda	Proasellus ortizi	whole organism	1	1
Isopoda	Proasellus parvulus	whole organism	1	1
Isopoda	Proasellus racovitzai	whole organism	1	1
Isopoda	Proasellus rectus	whole organism	2	2
Isopoda	Proasellus solanasi	whole organism	1	1
Isopoda	Proasellus spelaeus	whole organism	1	1
Mysida	Neomysis awatschensis	whole organism	0	0
			**Total malacostracan genes**	61

Additional file 4D. MyD88.

Arthropoda

Class (subphylum)	Species	Tissue type	Total gene counts	References
Insecta	Drosophila melanogaster	whole organism	1	Palmer et al., 2015
Insecta	Anopheles gambiae	whole organism	1	McTaggart et al., 2009
Insecta	Aedes aegypti	whole organism	1	McTaggart et al., 2009
Chilopoda (Myriapoda)	Strigamia maritima	whole organism	1	Palmer et al., 2015
Arachnida (Chelicerata)	Mesobuthus martensii	whole organism	1	Palmer et al., 2015
Arachnida (Chelicerata)	Ixodes scapularis	whole organism	1	Palmer et al., 2015
Branchiopoda (Crustacea)	Daphnia pulex	whole organism	1	McTaggart et al., 2009

Malacostraca

Order	Species/Datasets	Tissue type	Total gene counts	Total number of non-redundant genes per species
Amphipoda	Echinogammarus veneris	NA	0	0
Amphipoda	Gammarus chevreuxi	NA	0	0
Amphipoda	Gammarus pulex	NA	0	0
Amphipoda	Hyalella azteca_1	NA	0	0
Amphipoda	Hyalella azteca_2	NA	0	0
Amphipoda	Hyalella azteca_3	whole organism	0	0
Amphipoda	Melita plumulosa	whole organism	0	0
Amphipoda	Parhyale hawaiensis	whole organism	1	1
Amphipoda	Talitrus saltator	brain	1	1
Decapoda	Astacus astacus	hepatopancreas, ovaries, green glands, abdominal musculature	1	1
Decapoda	Astacus leptodactylus_1	hypodermis; Y organ	1	
Decapoda	Astacus leptodactylus_2	hepatopancreas	0	
Decapoda	Astacus leptodactylus_3	hypodermis, Y organ, hepatopancreas, gills, hematocytes, muscle	1	1
Decapoda	Callinectes sapidus	gill 7	1	1
Decapoda	Cancer borealis	nervous system	1	1
Decapoda	Carcinus maenas	NA	1	1
Decapoda	Cherax quadricarinatus_1	hypodermis and gastrolith disc	0	
Decapoda	Cherax quadricarinatus_2	heart, kidney, liver, nerve, testis	1	
Decapoda	Cherax quadricarinatus_3	heart, kidney, liver, nerve, testis	1	1
Decapoda	Eriocheir sinensis_1	NA	1	
Decapoda	Eriocheir sinensis_2	eyestalk, Y-organ, and hepatopancreas	1	
Decapoda	Eriocheir sinensis_3	hepatopancreas	0	1
Decapoda	Farfantepenaeus aztecus	hepatopancreas	1	1
Decapoda	Homarus americanus	nervous system	1	1
Decapoda	Hyas araneus_1	adult	0	
Decapoda	Hyas araneus_2	gill	1	1
Decapoda	Litopenaeus vannamei_1	Ghaffari et al., 2014	1	
Decapoda	Litopenaeus vannamei_2	hepatopancreas	1	
Decapoda	Litopenaeus vannamei_3	hepatopancreas	1	
Decapoda	Litopenaeus vannamei_4	hemocytes	1	1
Decapoda	Macrobrachium nipponense	NA	1	1
Decapoda	Pacifastacus leniusculus	Brain, HPT, Hemocyte, Hepatopancreas	0	0
Decapoda	Palaemon argentinus	whole organism	0	0
Decapoda	Penaeus monodon_1	hepatopancreas	1	
Decapoda	Penaeus monodon_2	hepatopancreas	1	1
Decapoda	Procambarus clarkii_1	Eyestalk	1	1
Decapoda	Procambarus clarkii_2	Eyestalk, brain, hemocytes, gills, testis, ovary, hepatopancreas, heart, green gland, ventralganglia, Y-organ, hypodermis, muscle	1	1
Decapoda	Scylla olivacea	Na	0	0
Decapoda	Scylla paramamosain	gill	0	0
Euphausiacea	Euphausia superba	NA	0	0
Euphausiacea	Meganyctiphanes norvegica	adult	0	0
Isopoda	Asellus aquaticus	NA	1	1
Isopoda	Bragasellus molinai	whole organism	1	1
Isopoda	Bragasellus peltatus	whole organism	1	1
Isopoda	Proasellus aragonensis	whole organism	1	1
Isopoda	Proasellus arthrodilus	whole organism	1	1
Isopoda	Proasellus assaforensis	whole organism	1	1
Isopoda	Proasellus beticus	whole organism	1	1
Isopoda	Proasellus cantabricus	whole organism	1	1
Isopoda	Proasellus cavaticus	whole organism	1	1
Isopoda	Proasellus coiffaiti	whole organism	1	1
Isopoda	Proasellus coxalis	whole organism	1	1
Isopoda	Proasellus ebrensis	whole organism	1	1

Isopoda	Proasellus escolai	whole organism	1	1
Isopoda	Proasellus grafi	whole organism	1	1
Isopoda	Proasellus granadensis	whole organism	1	1
Isopoda	Proasellus hercegovinensis	whole organism	1	1
Isopoda	Proasellus ibericus	whole organism	1	1
Isopoda	Proasellus jaloniacus	whole organism	1	1
Isopoda	Proasellus karamani	whole organism	1	1
Isopoda	Proasellus margalefi	whole organism	0	0
Isopoda	Proasellus meridianus	whole organism	1	1
Isopoda	Proasellus ortizi	whole organism	1	1
Isopoda	Proasellus parvulus	whole organism	1	1
Isopoda	Proasellus racovitzai	whole organism	1	1
Isopoda	Proasellus rectus	whole organism	1	1
Isopoda	Proasellus solanasi	whole organism	1	1
Isopoda	Proasellus spelaeus	whole organism	1	1
Mysida	Neomysis awatschensis	whole organism	1	1
			**Total malacostracan genes**	44

Additional file 4E. Cactus.

Arthropoda

Class (subphylum)	Species	Tissue type	Total gene counts	References
Insecta	Drosophila melanogaster	whole organism	1	Palmer et al., 2015
Insecta	Anopheles gambiae	whole organism	1	McTaggart et al., 2009
Insecta	Aedes aegypti	whole organism	1	McTaggart et al., 2009
Chilopoda (Myriapoda)	Strigamia maritima	whole organism	1	Palmer et al., 2015
Arachnida (Chelicerata)	Mesobuthus martensii	whole organism	2	Palmer et al., 2015
Arachnida (Chelicerata)	Ixodes scapularis	whole organism	1	Palmer et al., 2015
Branchiopoda (Crustacea)	Daphnia pulex	whole organism	2	McTaggart et al., 2009

Malacostraca

Order	Species/Datasets	Tissue type	Total gene counts	Total number of non-redundant genes per species
Amphipoda	Echinogammarus veneris	NA	0	0
Amphipoda	Gammarus chevreuxi	NA	1	1
Amphipoda	Gammarus pulex	NA	1	1
Amphipoda	Hyalella azteca_1	NA	0	
Amphipoda	Hyalella azteca_2	NA	0	
Amphipoda	Hyalella azteca_3	whole organism	1	1
Amphipoda	Melita plumulosa	whole organism	1	1
Amphipoda	Parhyale hawaiensis	whole organism	1	1
Amphipoda	Talitrus saltator	brain	1	1
Decapoda	Astacus astacus	hepatopancreas, ovaries, green glands, abdominal musculature	1	1
Decapoda	Astacus leptodactylus_1	hypodermis; Y organ	1	
Decapoda	Astacus leptodactylus_2	hepatopancreas	1	
Decapoda	Astacus leptodactylus_3	hypodermis, Y organ, hepatopancreas, gills, hematocytes, muscle	1	1
Decapoda	Callinectes sapidus	gill 7	1	1
Decapoda	Cancer borealis	nervous system	1	1
Decapoda	Carcinus maenas	NA	0	
Decapoda	Cherax quadricarinatus_1	hypodermis and gastrolith disc	1	
Decapoda	Cherax quadricarinatus_2	heart, kidney, liver, nerve, testis	1	
Decapoda	Cherax quadricarinatus_3	heart, kidney, liver, nerve, testis	1	1
Decapoda	Eriocheir sinensis_1	NA	1	
Decapoda	Eriocheir sinensis_2	eyestalk, Y-organ, and hepatopancreas	1	
Decapoda	Eriocheir sinensis_3	hepatopancreas	1	1
Decapoda	Farfantepenaeus aztecus	hepatopancreas	1	1
Decapoda	Homarus americanus	nervous system	1	1
Decapoda	Hyas araneus_1	adult	0	
Decapoda	Hyas araneus_2	gill	1	1
Decapoda	Litopenaeus vannamei_1	Ghaffari et al., 2014	1	
Decapoda	Litopenaeus vannamei_2	hepatopancreas	1	
Decapoda	Litopenaeus vannamei_3	hepatopancreas	1	
Decapoda	Litopenaeus vannamei_4	hemocytes	1	1
Decapoda	Macrobrachium nipponense	NA	1	1
Decapoda	Pacifastacus leniusculus	Brain, HPT, Hemocyte, Hepatopancreas	0	
Decapoda	Palaemon argentinus	whole organism	1	1
Decapoda	Penaeus monodon_1	hepatopancreas	1	
Decapoda	Penaeus monodon_2	hepatopancreas	1	1
Decapoda	Procambarus clarkii_1	Eyestalk	1	
Decapoda	Procambarus clarkii_2	Eyestalk, brain, hemocytes, gills, testis, ovary, hepatopancreas, heart, green gland, ventralganglia, Y-organ, hypodermis, muscle	1	1
Decapoda	Scylla olivacea	Na	1	1
Decapoda	Scylla paramamosain	gill	0	0
Euphausiacea	Euphausia superba	NA	0	0
Euphausiacea	Meganyctiphanes norvegica	adult	0	0
Isopoda	Asellus aquaticus	NA	1	1
Isopoda	Bragasellus molinai	whole organism	0	0
Isopoda	Bragasellus peltatus	whole organism	1	1
Isopoda	Proasellus aragonensis	whole organism	1	1
Isopoda	Proasellus arthrodilus	whole organism	1	1
Isopoda	Proasellus assaforensis	whole organism	1	1
Isopoda	Proasellus beticus	whole organism	0	0
Isopoda	Proasellus cantabricus	whole organism	1	1
Isopoda	Proasellus cavaticus	whole organism	1	1
Isopoda	Proasellus coiffaiti	whole organism	1	1
Isopoda	Proasellus coxalis	whole organism	1	1
Isopoda	Proasellus ebrensis	whole organism	1	1

Isopoda	Proasellus escolai	whole organism	0	0
Isopoda	Proasellus grafi	whole organism	1	1
Isopoda	Proasellus granadensis	whole organism	1	1
Isopoda	Proasellus hercegovinensis	whole organism	1	1
Isopoda	Proasellus ibericus	whole organism	1	1
Isopoda	Proasellus jaloniacus	whole organism	1	1
Isopoda	Proasellus karamani	whole organism	1	1
Isopoda	Proasellus margalefi	whole organism	1	1
Isopoda	Proasellus meridianus	whole organism	1	1
Isopoda	Proasellus ortizi	whole organism	1	1
Isopoda	Proasellus parvulus	whole organism	1	1
Isopoda	Proasellus racovitzai	whole organism	1	1
Isopoda	Proasellus rectus	whole organism	1	1
Isopoda	Proasellus solanasi	whole organism	1	1
Isopoda	Proasellus spelaeus	whole organism	1	1
Mysida	Neomysis awatschensis	whole organism	1	1
			**Total malacostracan genes**	46

Additional file 4F. Dorsal.

Arthropoda

Class (subphylum)	Species	Tissue type	Total gene counts	References
Insecta	Drosophila melanogaster	whole organism	1	Palmer et al., 2015
Insecta	Anopheles gambiae	whole organism	1	ImmunoDB
Insecta	Aedes aegypti	whole organism	1	ImmunoDB
Chilopoda (Myriapoda)	Strigamia maritima	whole organism	1	Palmer et al., 2015
Arachnida (Chelicerata)	Mesobuthus martensii	whole organism	1	Palmer et al., 2015
Arachnida (Chelicerata)	Ixodes scapularis	whole organism	1	Palmer et al., 2015
Branchiopoda (Crustacea)	Daphnia pulex	whole organism	1	Palmer et al., 2015

Malacostraca

Order	Species/Datasets	Tissue type	Total gene counts	Total number of non-redundant genes per species
Amphipoda	Echinogammarus veneris	NA	0	0
Amphipoda	Gammarus chevreuxi	NA	0	0
Amphipoda	Gammarus pulex	NA	0	0
Amphipoda	Hyalella azteca_1	NA	0	0
Amphipoda	Hyalella azteca_2	NA	0	
Amphipoda	Hyalella azteca_3	whole organism	1	1
Amphipoda	Melita plumulosa	whole organism	0	0
Amphipoda	Parhyale hawaiensis	whole organism	1	1
Amphipoda	Talitrus saltator	brain	1	1
Decapoda	Astacus astacus	hepatopancreas, ovaries, green glands, abdominal musculature	0	0
Decapoda	Astacus leptodactylus_1	hypodermis; Y organ	1	
Decapoda	Astacus leptodactylus_2	hepatopancreas	1	
Decapoda	Astacus leptodactylus_3	hypodermis, Y organ, hepatopancreas, gills, hematocytes, muscle	1	1
Decapoda	Callinectes sapidus	gill 7	1	1
Decapoda	Cancer borealis	nervous system	1	1
Decapoda	Carcinus maenas	NA	1	1
Decapoda	Cherax quadricarinatus_1	hypodermis and gastrolith disc	1	
Decapoda	Cherax quadricarinatus_2	heart, kidney, liver, nerve, testis	1	
Decapoda	Cherax quadricarinatus_3	heart, kidney, liver, nerve, testis	1	1
Decapoda	Eriocheir sinensis_1	NA	1	
Decapoda	Eriocheir sinensis_2	eyestalk, Y-organ, and hepatopancreas	1	
Decapoda	Eriocheir sinensis_3	hepatopancreas	1	1
Decapoda	Farfantepenaeus aztecus	hepatopancreas	1	1
Decapoda	Homarus americanus	nervous system	1	1
Decapoda	Hyas araneus_1	adult	0	
Decapoda	Hyas araneus_2	gill	1	1
Decapoda	Litopenaeus vannamei_1	Ghaffari et al., 2014	1	
Decapoda	Litopenaeus vannamei_2	hepatopancreas	1	
Decapoda	Litopenaeus vannamei_3	hepatopancreas	1	
Decapoda	Litopenaeus vannamei_4	hemocytes	1	1
Decapoda	Macrobrachium nipponense	NA	1	1
Decapoda	Pacifastacus leniusculus	Brain, HPT, Hemocyte, Hepatopancreas	1	1
Decapoda	Palaemon argentinus	whole organism	0	0
Decapoda	Penaeus monodon_1	hepatopancreas	1	
Decapoda	Penaeus monodon_2	hepatopancreas	1	1
Decapoda	Procambarus clarkii_1	Eyestalk	1	1
Decapoda	Procambarus clarkii_2	Eyestalk, brain, hemocytes, gills, testis, ovary, hepatopancreas, heart, green gland, ventralganglia, Y-organ, hypodermis, muscle	1	1
Decapoda	Scylla olivacea	Na	1	
Decapoda	Scylla paramamosain	gill	0	0
Euphausiacea	Euphausia superba	NA	1	1
Euphausiacea	Meganyctiphanes norvegica	adult	0	0
Isopoda	Asellus aquaticus	NA	0	0
Isopoda	Bragasellus molinai	whole organism	0	0
Isopoda	Bragasellus peltatus	whole organism	2	2
Isopoda	Proasellus aragonensis	whole organism	1	1
Isopoda	Proasellus arthrodilus	whole organism	1	1
Isopoda	Proasellus assaforensis	whole organism	1	1
Isopoda	Proasellus beticus	whole organism	1	1
Isopoda	Proasellus cantabricus	whole organism	1	1
Isopoda	Proasellus cavaticus	whole organism	1	1

Isopoda	Proasellus coiffaiti	whole organism	1	1
Isopoda	Proasellus coxalis	whole organism	1	1
Isopoda	Proasellus ebrensis	whole organism	1	1
Isopoda	Proasellus escolai	whole organism	1	1
Isopoda	Proasellus grafi	whole organism	1	1
Isopoda	Proasellus granadensis	whole organism	1	1
Isopoda	Proasellus hercegovinensis	whole organism	1	1
Isopoda	Proasellus ibericus	whole organism	1	1
Isopoda	Proasellus jaloniacus	whole organism	1	1
Isopoda	Proasellus karamani	whole organism	1	1
Isopoda	Proasellus margalefi	whole organism	1	1
Isopoda	Proasellus meridianus	whole organism	1	1
Isopoda	Proasellus ortizi	whole organism	1	1
Isopoda	Proasellus parvulus	whole organism	1	1
Isopoda	Proasellus racovitzai	whole organism	1	1
Isopoda	Proasellus rectus	whole organism	1	1
Isopoda	Proasellus solanasi	whole organism	1	1
Isopoda	Proasellus spelaeus	whole organism	1	1
Mysida	Neomysis awatschensis	whole organism	0	0
			**Total malacostracan genes**	45

Additional file 4G. Spätzle.

Arthropoda

Class (subphylum)	Species	Tissue type	Total gene counts	References
Insecta	Drosophila melanogaster	whole organism	6	Palmer et al., 2015
Insecta	Anopheles gambiae	whole organism	5	ImmunoDB
Insecta	Aedes aegypti	whole organism	6	ImmunoDB
Chilopoda (Myriapoda)	Strigamia maritima	whole organism	2	Palmer et al., 2015
Arachnida (Chelicerata)	Mesobuthus martensii	whole organism	5	Palmer et al., 2015
Arachnida (Chelicerata)	Ixodes scapularis	whole organism	2	Palmer et al., 2015
Branchiopoda (Crustacea)	Daphnia pulex	whole organism	4	Palmer et al., 2015

Malacostraca

Order	Species/Datasets	Tissue type	Total gene counts	Total number of non-redundant genes per species
Amphipoda	Echinogammarus veneris	NA	0	0
Amphipoda	Gammarus chevreuxi	NA	3	3
Amphipoda	Gammarus pulex	NA	0	0
Amphipoda	Hyalella azteca_1	NA	0	
Amphipoda	Hyalella azteca_2	NA	0	
Amphipoda	Hyalella azteca_3	whole organism	3	3
Amphipoda	Melita plumulosa	whole organism	3	3
Amphipoda	Parhyale hawaiensis	whole organism	7	7
Amphipoda	Talitrus saltator	brain	2	2
Decapoda	Astacus astacus	hepatopancreas, ovaries, green glands, abdominal musculature	0	0
Decapoda	Astacus leptodactylus_1	hypodermis; Y organ	3	
Decapoda	Astacus leptodactylus_2	hepatopancreas	2	
Decapoda	Astacus leptodactylus_3	hypodermis, Y organ, hepatopancreas, gills, hematocytes, muscle	4	6
Decapoda	Callinectes sapidus	gill 7	1	1
Decapoda	Cancer borealis	nervous system	4	4
Decapoda	Carcinus maenas	NA	4	4
Decapoda	Cherax quadricarinatus_1	hypodermis and gastrolith disc	3	
Decapoda	Cherax quadricarinatus_2	heart, kidney, liver, nerve, testis	3	
Decapoda	Cherax quadricarinatus_3	heart, kidney, liver, nerve, testis	3	3
Decapoda	Eriocheir sinensis_1	NA	6	
Decapoda	Eriocheir sinensis_2	eyestalk, Y-organ, and hepatopancreas	0	
Decapoda	Eriocheir sinensis_3	hepatopancreas	0	6
Decapoda	Farfantepenaeus aztecus	hepatopancreas	2	2
Decapoda	Homarus americanus	nervous system	2	2
Decapoda	Hyas araneus_1	adult	1	
Decapoda	Hyas araneus_2	gill	1	1
Decapoda	Litopenaeus vannamei_1	Ghaffari et al., 2014	3	
Decapoda	Litopenaeus vannamei_2	hepatopancreas	0	
Decapoda	Litopenaeus vannamei_3	hepatopancreas	0	
Decapoda	Litopenaeus vannamei_4	hemocytes	0	3
Decapoda	Macrobrachium nipponense	NA	0	0
Decapoda	Pacifastacus leniusculus	Brain, HPT, Hemocyte, Hepatopancreas	0	0
Decapoda	Palaemon argentinus	whole organism	0	0
Decapoda	Penaeus monodon_1	hepatopancreas	0	
Decapoda	Penaeus monodon_2	hepatopancreas	0	0
Decapoda	Procambarus clarkii_1	Eyestalk	3	
Decapoda	Procambarus clarkii_2	Eyestalk, brain, hemocytes, gills, testis, ovary, hepatopancreas, heart, green gland, ventralganglia, Y-organ, hypodermis, muscle	3	3
Decapoda	Scylla olivacea	Na	2	2
Decapoda	Scylla paramamosain	gill	4	4
Euphausiacea	Euphausia superba	NA	26	26
Euphausiacea	Meganyctiphanes norvegica	adult	5	5
Isopoda	Asellus aquaticus	NA	1	1
Isopoda	Bragasellus molinai	whole organism	1	1
Isopoda	Bragasellus peltatus	whole organism	2	2
Isopoda	Proasellus aragonensis	whole organism	5	5
Isopoda	Proasellus arthrodilus	whole organism	1	1
Isopoda	Proasellus assaforensis	whole organism	1	1
Isopoda	Proasellus beticus	whole organism	0	0
Isopoda	Proasellus cantabricus	whole organism	2	2
Isopoda	Proasellus cavaticus	whole organism	3	3

Isopoda	Proasellus coiffaiti	whole organism	3	3
Isopoda	Proasellus coxalis	whole organism	5	5
Isopoda	Proasellus ebrensis	whole organism	4	4
Isopoda	Proasellus escolai	whole organism	1	1
Isopoda	Proasellus grafi	whole organism	2	2
Isopoda	Proasellus granadensis	whole organism	3	3
Isopoda	Proasellus hercegovinensis	whole organism	3	3
Isopoda	Proasellus ibericus	whole organism	1	1
Isopoda	Proasellus jaloniacus	whole organism	5	5
Isopoda	Proasellus karamani	whole organism	5	5
Isopoda	Proasellus margalefi	whole organism	2	2
Isopoda	Proasellus meridianus	whole organism	5	5
Isopoda	Proasellus ortizi	whole organism	3	3
Isopoda	Proasellus parvulus	whole organism	3	3
Isopoda	Proasellus racovitzai	whole organism	5	5
Isopoda	Proasellus rectus	whole organism	4	4
Isopoda	Proasellus solanasi	whole organism	7	7
Isopoda	Proasellus spelaeus	whole organism	5	5
Mysida	Neomysis awatschensis	whole organism	7	7
			**Total malacostracan genes**	179

Additional file 4H. Tollip.

Arthropoda

Class (subphylum)	Species	Tissue type	Total gene counts	References
Insecta	Drosophila melanogaster	whole organism	0	proteome
Insecta	Anopheles gambiae	whole organism	1	proteome
Insecta	Aedes aegypti	whole organism	0	proteome
Chilopoda (Myriapoda)	Strigamia maritima	whole organism	1	proteome
Arachnida (Chelicerata)	Mesobuthus martensii	whole organism	1	proteome
Arachnida (Chelicerata)	Ixodes scapularis	whole organism	1	proteome
Branchiopoda (Crustacea)	Daphnia pulex	whole organism	1	proteome

Malacostraca

Order	Species/Datasets	Tissue type	Total gene counts	Total number of non-redundant genes per species
Amphipoda	Echinogammarus veneris	NA	0	0
Amphipoda	Gammarus chevreuxi	NA	1	1
Amphipoda	Gammarus pulex	NA	1	1
Amphipoda	Hyalella azteca_1	NA	0	0
Amphipoda	Hyalella azteca_2	NA	0	0
Amphipoda	Hyalella azteca_3	whole organism	0	0
Amphipoda	Melita plumulosa	whole organism	0	0
Amphipoda	Parhyale hawaiensis	whole organism	1	1
Amphipoda	Talitrus saltator	brain	1	1
Decapoda	Astacus astacus	hepatopancreas, ovaries, green glands, abdominal musculature	1	1
Decapoda	Astacus leptodactylus_1	hypodermis; Y organ	1	
Decapoda	Astacus leptodactylus_2	hepatopancreas	1	
Decapoda	Astacus leptodactylus_3	hypodermis, Y organ, hepatopancreas, gills, hematocytes, muscle	1	1
Decapoda	Callinectes sapidus	gill 7	0	0
Decapoda	Cancer borealis	nervous system	1	1
Decapoda	Carcinus maenas	NA	1	1
Decapoda	Cherax quadricarinatus_1	hypodermis and gastrolith disc	1	
Decapoda	Cherax quadricarinatus_2	heart, kidney, liver, nerve, testis	1	
Decapoda	Cherax quadricarinatus_3	heart, kidney, liver, nerve, testis	1	1
Decapoda	Eriocheir sinensis_1	NA	1	
Decapoda	Eriocheir sinensis_2	eyestalk, Y-organ, and hepatopancreas	1	
Decapoda	Eriocheir sinensis_3	hepatopancreas	1	1
Decapoda	Farfantepenaeus aztecus	hepatopancreas	1	1
Decapoda	Homarus americanus	nervous system	1	1
Decapoda	Hyas araneus_1	adult	1	
Decapoda	Hyas araneus_2	gill	2	2
Decapoda	Litopenaeus vannamei_1	Ghaffari et al., 2014	1	
Decapoda	Litopenaeus vannamei_2	hepatopancreas	1	
Decapoda	Litopenaeus vannamei_3	hepatopancreas	1	
Decapoda	Litopenaeus vannamei_4	hemocytes	1	1
Decapoda	Macrobrachium nipponense	NA	1	1
Decapoda	Pacifastacus leniusculus	Brain, HPT, Hemocyte, Hepatopancreas	1	1
Decapoda	Palaemon argentinus	whole organism	1	1
Decapoda	Penaeus monodon_1	hepatopancreas	1	
Decapoda	Penaeus monodon_2	hepatopancreas	1	1
Decapoda	Procambarus clarkii_1	Eyestalk	1	
Decapoda	Procambarus clarkii_2	Eyestalk, brain, hemocytes, gills, testis, ovary, hepatopancreas, heart, green gland, ventralganglia, Y-organ, hypodermis, muscle	1	1
Decapoda	Scylla olivacea	Na	1	1
Decapoda	Scylla paramamosain	gill	0	0
Euphausiacea	Euphausia superba	NA	1	1
Euphausiacea	Meganyctiphanes norvegica	adult	0	0
Isopoda	Asellus aquaticus	NA	0	0
Isopoda	Bragasellus molinai	whole organism	1	1
Isopoda	Bragasellus peltatus	whole organism	1	1
Isopoda	Proasellus aragonensis	whole organism	1	1
Isopoda	Proasellus arthrodilus	whole organism	1	1
Isopoda	Proasellus assaforensis	whole organism	1	1
Isopoda	Proasellus beticus	whole organism	0	0
Isopoda	Proasellus cantabricus	whole organism	1	1
Isopoda	Proasellus cavaticus	whole organism	1	1
Isopoda	Proasellus coiffaiti	whole organism	1	1
Isopoda	Proasellus coxalis	whole organism	1	1
Isopoda	Proasellus ebrensis	whole organism	1	1

Isopoda	Proasellus escolai	whole organism	1	1
Isopoda	Proasellus grafi	whole organism	1	1
Isopoda	Proasellus granadensis	whole organism	0	0
Isopoda	Proasellus hercegovinensis	whole organism	1	1
Isopoda	Proasellus ibericus	whole organism	1	1
Isopoda	Proasellus jaloniacus	whole organism	1	1
Isopoda	Proasellus karamani	whole organism	2	2
Isopoda	Proasellus margalefi	whole organism	0	0
Isopoda	Proasellus meridianus	whole organism	1	1
Isopoda	Proasellus ortizi	whole organism	1	1
Isopoda	Proasellus parvulus	whole organism	1	1
Isopoda	Proasellus racovitzai	whole organism	1	1
Isopoda	Proasellus rectus	whole organism	1	1
Isopoda	Proasellus solanasi	whole organism	1	1
Isopoda	Proasellus spelaeus	whole organism	1	1
Mysida	Neomysis awatschensis	whole organism	1	1
			**Total malacostracan genes**	47
