# Supplementary material for: Comparative genomic analysis of innate immunity reveals novel and conserved components in crustacean food crop species
Source: BMC Genomics. 2017 May 18;18:389. doi: 10.1186/s12864-017-3769-4 (PMC5437397; doi:10.1186/s12864-017-3769-4)
Supplement: Supplementary file 11 — Malacostracans Imd pathway components. (PDF 351 kb) [file 12864_2017_3769_MOESM11_ESM.pdf]

Additional file 5. Malacostracans Imd pathway components.

Additional file 5A. IMD.
Arthropoda

Class (subphylum)	Species	Tissue type	Total gene counts	References
Insecta	Drosophila melanogaster	whole organism	1	McTaggart et al., 2009
Insecta	Anopheles gambiae	whole organism	1	McTaggart et al., 2009
Insecta	Aedes aegypti	whole organism	1	McTaggart et al., 2009
Chilopoda (Myriapoda)	Strigamia maritima	whole organism	1	Palmer et al., 2015
Arachnida (Chelicerata)	Mesobuthus martensii	whole organism	0	Palmer et al., 2015
Arachnida (Chelicerata)	Ixodes scapularis	whole organism	0	Palmer et al., 2015
Branchiopoda (Crustacea)	Daphnia pulex	whole organism	1	McTaggart et al., 2009

Malacostraca

Order	Species/Datasets	Tissue type	Total gene counts	Total number of non-redundant genes per species
Amphipoda	Echinogammarus veneris	NA	0	0
Amphipoda	Gammarus chevreuxi	NA	1	1
Amphipoda	Gammarus pulex	NA	0	0
Amphipoda	Hyalella azteca_1	NA	1	
Amphipoda	Hyalella azteca_2	NA	1	
Amphipoda	Hyalella azteca_3	whole organism	0	1
Amphipoda	Melita plumulosa	whole organism	0	0
Amphipoda	Parhyale hawaiensis	whole organism	1	1
Amphipoda	Talitrus saltator	brain	1	1
Decapoda	Astacus astacus	hepatopancreas, ovaries, green glands, abdominal musculature	0	0
Decapoda	Astacus leptodactylus_1	hypodermis; Y organ	1	
Decapoda	Astacus leptodactylus_2	hepatopancreas	1	
Decapoda	Astacus leptodactylus_3	hypodermis, Y organ, hepatopancreas, gills, hematocytes, muscle	1	1
Decapoda	Callinectes sapidus	gill 7	0	0
Decapoda	Cancer borealis	nervous system	1	1
Decapoda	Carcinus maenas	NA	0	0
Decapoda	Cherax quadricarinatus_1	hypodermis and gastrolith disc	0	
Decapoda	Cherax quadricarinatus_2	heart, kidney, liver, nerve, testis	1	
Decapoda	Cherax quadricarinatus_3	heart, kidney, liver, nerve, testis	1	1
Decapoda	Eriocheir sinensis_1	NA	1	
Decapoda	Eriocheir sinensis_2	eyestalk, Y-organ, and hepatopancreas	0	
Decapoda	Eriocheir sinensis_3	hepatopancreas	0	1
Decapoda	Farfantepenaeus aztecus	hepatopancreas	1	1
Decapoda	Homarus americanus	nervous system	1	1
Decapoda	Hyas araneus_1	adult	0	
Decapoda	Hyas araneus_2	gill	1	1
Decapoda	Litopenaeus vannamei_1	Ghaffari et al., 2014	1	
Decapoda	Litopenaeus vannamei_2	hepatopancreas	0	
Decapoda	Litopenaeus vannamei_3	hepatopancreas	1	
Decapoda	Litopenaeus vannamei_4	hemocytes	1	1
Decapoda	Macrobrachium nipponense	NA	1	1
Decapoda	Pacifastacus leniusculus	Brain, HPT, Hemocyte, Hepatopancreas	1	1
Decapoda	Palaemon argentinus	whole organism	0	0
Decapoda	Penaeus monodon_1	hepatopancreas	1	
Decapoda	Penaeus monodon_2	hepatopancreas	1	1
Decapoda	Procambarus clarkii_1	Eyestalk	1	
Decapoda	Procambarus clarkii_2	Eyestalk, brain, hemocytes, gills, testis, ovary, hepatopancreas, heart, green gland, ventralganglia, Y-organ, hypodermis, muscle	1	1
Decapoda	Scylla olivacea	Na	0	0
Decapoda	Scylla paramamosain	gill	0	0
Euphausiacea	Euphausia superba	NA	1	1
Euphausiacea	Meganyctiphanes norvegica	adult	0	0
Isopoda	Asellus aquaticus	NA	0	0
Isopoda	Bragasellus molinai	whole organism	1	1
Isopoda	Bragasellus peltatus	whole organism	1	1
Isopoda	Proasellus aragonensis	whole organism	1	1
Isopoda	Proasellus arthrodilus	whole organism	1	1
Isopoda	Proasellus assaforensis	whole organism	0	0
Isopoda	Proasellus beticus	whole organism	0	0
Isopoda	Proasellus cantabricus	whole organism	1	1
Isopoda	Proasellus cavaticus	whole organism	1	1
Isopoda	Proasellus coiffaiti	whole organism	1	1

Isopoda	Proasellus coxalis	whole organism	1	1
Isopoda	Proasellus ebrensis	whole organism	1	1
Isopoda	Proasellus escolai	whole organism	1	1
Isopoda	Proasellus grafi	whole organism	1	1
Isopoda	Proasellus granadensis	whole organism	0	0
Isopoda	Proasellus hercegovinensis	whole organism	1	1
Isopoda	Proasellus ibericus	whole organism	1	1
Isopoda	Proasellus jaloniacus	whole organism	1	1
Isopoda	Proasellus karamani	whole organism	0	0
Isopoda	Proasellus margalefi	whole organism	1	1
Isopoda	Proasellus meridianus	whole organism	1	1
Isopoda	Proasellus ortizi	whole organism	1	1
Isopoda	Proasellus parvulus	whole organism	1	1
Isopoda	Proasellus racovitzai	whole organism	1	1
Isopoda	Proasellus rectus	whole organism	0	0
Isopoda	Proasellus solanasi	whole organism	1	1
Isopoda	Proasellus spelaeus	whole organism	1	1
Mysida	Neomysis awatschensis	whole organism	1	1
			**Total malacostracan genes**	39

Additional file 5B. Relish.
Arthropoda

Class (subphylum)	Species	Tissue type	Total gene counts	References
Insecta	Drosophila melanogaster	whole organism	1	Palmer et al., 2015
Insecta	Anopheles gambiae	whole organism	2	McTaggart et al., 2009
Insecta	Aedes aegypti	whole organism	3	McTaggart et al., 2009
Chilopoda (Myriapoda)	Strigamia maritima	whole organism	1	Palmer et al., 2015
Arachnida (Chelicerata)	Mesobuthus martensii	whole organism	1	Palmer et al., 2015
Arachnida (Chelicerata)	Ixodes scapularis	whole organism	1	Palmer et al., 2015
Branchiopoda (Crustacea)	Daphnia pulex	whole organism	4	Palmer et al., 2015

Malacostraca

Order	Species/Datasets	Tissue type	Total gene counts	Total number of non-redundant genes per species
Amphipoda	Echinogammarus veneris	NA	0	0
Amphipoda	Gammarus chevreuxi	NA	0	0
Amphipoda	Gammarus pulex	NA	0	0
Amphipoda	Hyalella azteca_1	NA	0	
Amphipoda	Hyalella azteca_2	NA	0	
Amphipoda	Hyalella azteca_3	whole organism	0	0
Amphipoda	Melita plumulosa	whole organism	1	1
Amphipoda	Parhyale hawaiensis	whole organism	1	1
Amphipoda	Talitrus saltator	brain	1	1
Decapoda	Astacus astacus	hepatopancreas, ovaries, green glands, abdominal musculature	1	1
Decapoda	Astacus leptodactylus_1	hypodermis; Y organ	1	
Decapoda	Astacus leptodactylus_2	hepatopancreas	1	
Decapoda	Astacus leptodactylus_3	hypodermis, Y organ, hepatopancreas, gills, hematocytes, muscle	1	1
Decapoda	Callinectes sapidus	gill 7	1	1
Decapoda	Cancer borealis	nervous system	1	1
Decapoda	Carcinus maenas	NA	1	1
Decapoda	Cherax quadricarinatus_1	hypodermis and gastrolith disc	0	
Decapoda	Cherax quadricarinatus_2	heart, kidney, liver, nerve, testis	1	
Decapoda	Cherax quadricarinatus_3	heart, kidney, liver, nerve, testis	1	1
Decapoda	Eriocheir sinensis_1	NA	0	
Decapoda	Eriocheir sinensis_2	eyestalk, Y-organ, and hepatopancreas	1	
Decapoda	Eriocheir sinensis_3	hepatopancreas	1	1
Decapoda	Farfantepenaeus aztecus	hepatopancreas	1	1
Decapoda	Homarus americanus	nervous system	1	1
Decapoda	Hyas araneus_1	adult	1	
Decapoda	Hyas araneus_2	gill	1	1
Decapoda	Litopenaeus vannamei_1	Ghaffari et al., 2014	1	
Decapoda	Litopenaeus vannamei_2	hepatopancreas	1	
Decapoda	Litopenaeus vannamei_3	hepatopancreas	1	
Decapoda	Litopenaeus vannamei_4	hemocytes	1	1
Decapoda	Macrobrachium nipponense	NA	1	1
Decapoda	Pacifastacus leniusculus	Brain, HPT, Hemocyte, Hepatopancreas	1	1
Decapoda	Palaemon argentinus	whole organism	0	0
Decapoda	Penaeus monodon_1	hepatopancreas	1	
Decapoda	Penaeus monodon_2	hepatopancreas	1	1
Decapoda	Procambarus clarkii_1	Eyestalk	1	
Decapoda	Procambarus clarkii_2	Eyestalk, brain, hemocytes, gills, testis, ovary, hepatopancreas, heart, green gland, ventralganglia, Y-organ, hypodermis, muscle	1	1
Decapoda	Scylla olivacea	Na	1	1
Decapoda	Scylla paramamosain	gill	0	0
Euphausiacea	Euphausia superba	NA	1	1
Euphausiacea	Meganyctiphanes norvegica	adult	1	1
Isopoda	Asellus aquaticus	NA	1	1
Isopoda	Bragasellus molinai	whole organism	1	1
Isopoda	Bragasellus peltatus	whole organism	1	1
Isopoda	Proasellus aragonensis	whole organism	1	1
Isopoda	Proasellus arthrodilus	whole organism	1	1
Isopoda	Proasellus assaforensis	whole organism	1	1
Isopoda	Proasellus beticus	whole organism	1	1
Isopoda	Proasellus cantabricus	whole organism	1	1
Isopoda	Proasellus cavaticus	whole organism	1	1
Isopoda	Proasellus coiffaiti	whole organism	1	1
Isopoda	Proasellus coxalis	whole organism	1	1

Isopoda	Proasellus ebrensis	whole organism	1	1
Isopoda	Proasellus escolai	whole organism	1	1
Isopoda	Proasellus grafi	whole organism	1	1
Isopoda	Proasellus granadensis	whole organism	1	1
Isopoda	Proasellus hercegovinensis	whole organism	1	1
Isopoda	Proasellus ibericus	whole organism	1	1
Isopoda	Proasellus jaloniacus	whole organism	1	1
Isopoda	Proasellus karamani	whole organism	1	1
Isopoda	Proasellus margalefi	whole organism	1	1
Isopoda	Proasellus meridianus	whole organism	1	1
Isopoda	Proasellus ortizi	whole organism	1	1
Isopoda	Proasellus parvulus	whole organism	1	1
Isopoda	Proasellus racovitzai	whole organism	1	1
Isopoda	Proasellus rectus	whole organism	1	1
Isopoda	Proasellus solanasi	whole organism	1	1
Isopoda	Proasellus spelaeus	whole organism	1	1
Mysida	Neomysis awatschensis	whole organism	1	1
			**Total malacostracan genes**	49

Additional file 5C. Caspar.

Arthropoda

Class (subphylum)	Species	Tissue type	Total gene counts	References
Insecta	Drosophila melanogaster	whole organism	1	proteome
Insecta	Anopheles gambiae	whole organism	1	proteome
Insecta	Aedes aegypti	whole organism	1	proteome
Chilopoda (Myriapoda)	Strigamia maritima	whole organism	1	proteome
Arachnida (Chelicerata)	Mesobuthus martensii	whole organism	1	proteome
Arachnida (Chelicerata)	Ixodes scapularis	whole organism	1	proteome
Branchiopoda (Crustacea)	Daphnia pulex	whole organism	1	proteome

Malacostraca

Order	Species/Datasets	Tissue type	Total gene counts	Total number of non-redundant genes per species
Amphipoda	Echinogammarus veneris	NA	0	0
Amphipoda	Gammarus chevreuxi	NA	1	1
Amphipoda	Gammarus pulex	NA	0	0
Amphipoda	Hyalella azteca_1	NA	0	
Amphipoda	Hyalella azteca_2	NA	0	
Amphipoda	Hyalella azteca_3	whole organism	0	0
Amphipoda	Melita plumulosa	whole organism	0	0
Amphipoda	Parhyale hawaiensis	whole organism	1	1
Amphipoda	Talitrus saltator	brain	1	1
Decapoda	Astacus astacus	hepatopancreas, ovaries, green glands, abdominal musculature	1	1
Decapoda	Astacus leptodactylus_1	hypodermis; Y organ	1	
Decapoda	Astacus leptodactylus_2	hepatopancreas	1	
Decapoda	Astacus leptodactylus_3	hypodermis, Y organ, hepatopancreas, gills, hematocytes, muscle	1	1
Decapoda	Callinectes sapidus	gill 7	1	1
Decapoda	Cancer borealis	nervous system	1	1
Decapoda	Carcinus maenas	NA	0	0
Decapoda	Cherax quadricarinatus_1	hypodermis and gastrolith disc	1	
Decapoda	Cherax quadricarinatus_2	heart, kidney, liver, nerve, testis	1	
Decapoda	Cherax quadricarinatus_3	heart, kidney, liver, nerve, testis	1	1
Decapoda	Eriocheir sinensis_1	NA	1	
Decapoda	Eriocheir sinensis_2	eyestalk, Y-organ, and hepatopancreas	1	
Decapoda	Eriocheir sinensis_3	hepatopancreas	1	1
Decapoda	Farfantepenaeus aztecus	hepatopancreas	1	1
Decapoda	Homarus americanus	nervous system	1	1
Decapoda	Hyas araneus_1	adult	0	
Decapoda	Hyas araneus_2	gill	1	1
Decapoda	Litopenaeus vannamei_1	Ghaffari et al., 2014	1	
Decapoda	Litopenaeus vannamei_2	hepatopancreas	1	
Decapoda	Litopenaeus vannamei_3	hepatopancreas	1	
Decapoda	Litopenaeus vannamei_4	hemocytes	1	1
Decapoda	Macrobrachium nipponense	NA	1	1
Decapoda	Pacifastacus leniusculus	Brain, HPT, Hemocyte, Hepatopancreas	1	1
Decapoda	Palaemon argentinus	whole organism	1	1
Decapoda	Penaeus monodon_1	hepatopancreas	1	
Decapoda	Penaeus monodon_2	hepatopancreas	1	1
Decapoda	Procambarus clarkii_1	Eyestalk	1	
Decapoda	Procambarus clarkii_2	Eyestalk, brain, hemocytes, gills, testis, ovary, hepatopancreas, heart, green gland, ventralganglia, Y-organ, hypodermis, muscle	1	1
Decapoda	Scylla olivacea	Na	1	1
Decapoda	Scylla paramamosain	gill	0	0
Euphausiacea	Euphausia superba	NA	1	1
Euphausiacea	Meganyctiphanes norvegica	adult	1	1
Isopoda	Asellus aquaticus	NA	1	1
Isopoda	Bragasellus molinai	whole organism	1	1
Isopoda	Bragasellus peltatus	whole organism	1	1
Isopoda	Proasellus aragonensis	whole organism	1	1
Isopoda	Proasellus arthrodilus	whole organism	1	1
Isopoda	Proasellus assaforensis	whole organism	1	1
Isopoda	Proasellus beticus	whole organism	0	0
Isopoda	Proasellus cantabricus	whole organism	1	1
Isopoda	Proasellus cavaticus	whole organism	1	1
Isopoda	Proasellus coiffaiti	whole organism	1	1
Isopoda	Proasellus coxalis	whole organism	1	1
Isopoda	Proasellus ebrensis	whole organism	1	1

Isopoda	Proasellus escolai	whole organism	1	1
Isopoda	Proasellus grafi	whole organism	1	1
Isopoda	Proasellus granadensis	whole organism	1	1
Isopoda	Proasellus hercegovinensis	whole organism	1	1
Isopoda	Proasellus ibericus	whole organism	1	1
Isopoda	Proasellus jaloniacus	whole organism	1	1
Isopoda	Proasellus karamani	whole organism	1	1
Isopoda	Proasellus margalefi	whole organism	1	1
Isopoda	Proasellus meridianus	whole organism	1	1
Isopoda	Proasellus ortizi	whole organism	1	1
Isopoda	Proasellus parvulus	whole organism	1	1
Isopoda	Proasellus racovitzai	whole organism	1	1
Isopoda	Proasellus rectus	whole organism	1	1
Isopoda	Proasellus solanasi	whole organism	1	1
Isopoda	Proasellus spelaeus	whole organism	1	1
Mysida	Neomysis awatschensis	whole organism	1	1
			**Total malacostracan genes**	48

Additional file 5D. DREDD.

Arthropoda

Class (subphylum)	Species	Tissue type	Total gene counts	References
Insecta	Drosophila melanogaster	whole organism	1	Palmer et al., 2015
Insecta	Anopheles gambiae	whole organism	1	Uniprot
Insecta	Aedes aegypti	whole organism	1	Uniprot
Chilopoda (Myriapoda)	Strigamia maritima	whole organism	0	Palmer et al., 2015
Arachnida (Chelicerata)	Mesobuthus martensii	whole organism	1	Palmer et al., 2015
Arachnida (Chelicerata)	Ixodes scapularis	whole organism	0	Palmer et al., 2015
Branchiopoda (Crustacea)	Daphnia pulex	whole organism	1	Uniprot

Malacostraca

Order	Species/Datasets	Tissue type	Total gene counts	Total number of non-redundant genes per species
Amphipoda	Echinogammarus veneris	NA	0	0
Amphipoda	Gammarus chevreuxi	NA	0	0
Amphipoda	Gammarus pulex	NA	0	0
Amphipoda	Hyalella azteca_1	NA	0	
Amphipoda	Hyalella azteca_2	NA	1	
Amphipoda	Hyalella azteca_3	whole organism	1	1
Amphipoda	Melita plumulosa	whole organism	0	0
Amphipoda	Parhyale hawaiensis	whole organism	1	1
Amphipoda	Talitrus saltator	brain	1	1
Decapoda	Astacus astacus	hepatopancreas, ovaries, green glands, abdominal musculature	1	1
Decapoda	Astacus leptodactylus_1	hypodermis; Y organ	1	
Decapoda	Astacus leptodactylus_2	hepatopancreas	0	
Decapoda	Astacus leptodactylus_3	hypodermis, Y organ, hepatopancreas, gills, hematocytes, muscle	1	1
Decapoda	Callinectes sapidus	gill 7	1	1
Decapoda	Cancer borealis	nervous system	1	1
Decapoda	Carcinus maenas	NA	1	1
Decapoda	Cherax quadricarinatus_1	hypodermis and gastrolith disc	0	
Decapoda	Cherax quadricarinatus_2	heart, kidney, liver, nerve, testis	1	
Decapoda	Cherax quadricarinatus_3	heart, kidney, liver, nerve, testis	0	1
Decapoda	Eriocheir sinensis_1	NA	1	
Decapoda	Eriocheir sinensis_2	eyestalk, Y-organ, and hepatopancreas	0	
Decapoda	Eriocheir sinensis_3	hepatopancreas	0	1
Decapoda	Farfantepenaeus aztecus	hepatopancreas	1	1
Decapoda	Homarus americanus	nervous system	1	1
Decapoda	Hyas araneus_1	adult	0	
Decapoda	Hyas araneus_2	gill	1	1
Decapoda	Litopenaeus vannamei_1	Ghaffari et al., 2014	1	
Decapoda	Litopenaeus vannamei_2	hepatopancreas	1	
Decapoda	Litopenaeus vannamei_3	hepatopancreas	1	
Decapoda	Litopenaeus vannamei_4	hemocytes	1	1
Decapoda	Macrobrachium nipponense	NA	1	1
Decapoda	Pacifastacus leniusculus	Brain, HPT, Hemocyte, Hepatopancreas	0	0
Decapoda	Palaemon argentinus	whole organism	0	0
Decapoda	Penaeus monodon_1	hepatopancreas	1	
Decapoda	Penaeus monodon_2	hepatopancreas	1	1
Decapoda	Procambarus clarkii_1	Eyestalk	1	
Decapoda	Procambarus clarkii_2	Eyestalk, brain, hemocytes, gills, testis, ovary, hepatopancreas, heart, green gland, ventralganglia, Y-organ, hypodermis, muscle	1	1
Decapoda	Scylla olivacea	Na	0	0
Decapoda	Scylla paramamosain	gill	0	0
Euphausiacea	Euphausia superba	NA	0	0
Euphausiacea	Meganyctiphanes norvegica	adult	0	0
Isopoda	Asellus aquaticus	NA	0	0
Isopoda	Bragasellus molinai	whole organism	0	0
Isopoda	Bragasellus peltatus	whole organism	1	1
Isopoda	Proasellus aragonensis	whole organism	1	1
Isopoda	Proasellus arthrodilus	whole organism	1	1
Isopoda	Proasellus assaforensis	whole organism	1	1
Isopoda	Proasellus beticus	whole organism	1	1
Isopoda	Proasellus cantabricus	whole organism	1	1
Isopoda	Proasellus cavaticus	whole organism	1	1
Isopoda	Proasellus coiffaiti	whole organism	1	1
Isopoda	Proasellus coxalis	whole organism	1	1

Isopoda	Proasellus ebrensis	whole organism	1	1
Isopoda	Proasellus escolai	whole organism	1	1
Isopoda	Proasellus grafi	whole organism	1	1
Isopoda	Proasellus granadensis	whole organism	1	1
Isopoda	Proasellus hercegovinensis	whole organism	1	1
Isopoda	Proasellus ibericus	whole organism	1	1
Isopoda	Proasellus jaloniacus	whole organism	1	1
Isopoda	Proasellus karamani	whole organism	1	1
Isopoda	Proasellus margalefi	whole organism	1	1
Isopoda	Proasellus meridianus	whole organism	1	1
Isopoda	Proasellus ortizi	whole organism	1	1
Isopoda	Proasellus parvulus	whole organism	0	0
Isopoda	Proasellus racovitzai	whole organism	1	1
Isopoda	Proasellus rectus	whole organism	1	1
Isopoda	Proasellus solanasi	whole organism	1	1
Isopoda	Proasellus spelaeus	whole organism	1	1
Mysida	Neomysis awatschensis	whole organism	1	1
			**Total malacostracan genes**	42

Additional file 5E. IKKb (Ird5)

Arthropoda

Class (subphylum)	Species	Tissue type	Total gene counts	References
Insecta	Drosophila melanogaster	whole organism	1	Palmer et al., 2015
Insecta	Anopheles gambiae	whole organism	1	ImmunoDB
Insecta	Aedes aegypti	whole organism	1	ImmunoDB
Chilopoda (Myriapoda)	Strigamia maritima	whole organism	1	Palmer et al., 2015
Arachnida (Chelicerata)	Mesobuthus martensii	whole organism	1	Palmer et al., 2015
Arachnida (Chelicerata)	Ixodes scapularis	whole organism	1	Palmer et al., 2015
Branchiopoda (Crustacea)	Daphnia pulex	whole organism	1	Palmer et al., 2015

Malacostraca

Order	Species/Datasets	Tissue type	Total gene counts	Total number of non-redundant genes per species
Amphipoda	Echinogammarus veneris	NA	0	1
Amphipoda	Gammarus chevreuxi	NA	0	0
Amphipoda	Gammarus pulex	NA	0	0
Amphipoda	Hyalella azteca_1	NA	0	
Amphipoda	Hyalella azteca_2	NA	0	
Amphipoda	Hyalella azteca_3	whole organism	1	1
Amphipoda	Melita plumulosa	whole organism	0	0
Amphipoda	Parhyale hawaiensis	whole organism	1	1
Amphipoda	Talitrus saltator	brain	1	1
Decapoda	Astacus astacus	hepatopancreas, ovaries, green glands, abdominal musculature	1	1
Decapoda	Astacus leptodactylus_1	hypodermis; Y organ	1	
Decapoda	Astacus leptodactylus_2	hepatopancreas	0	
Decapoda	Astacus leptodactylus_3	hypodermis, Y organ, hepatopancreas, gills, hematocytes, muscle	1	1
Decapoda	Callinectes sapidus	gill 7	0	0
Decapoda	Cancer borealis	nervous system	1	1
Decapoda	Carcinus maenas	NA	1	1
Decapoda	Cherax quadricarinatus_1	hypodermis and gastrolith disc	0	
Decapoda	Cherax quadricarinatus_2	heart, kidney, liver, nerve, testis	1	
Decapoda	Cherax quadricarinatus_3	heart, kidney, liver, nerve, testis	1	1
Decapoda	Eriocheir sinensis_1	NA	1	
Decapoda	Eriocheir sinensis_2	eyestalk, Y-organ, and hepatopancreas	1	
Decapoda	Eriocheir sinensis_3	hepatopancreas	1	1
Decapoda	Farfantepenaeus aztecus	hepatopancreas	1	1
Decapoda	Homarus americanus	nervous system	1	1
Decapoda	Hyas araneus_1	adult	0	
Decapoda	Hyas araneus_2	gill	1	1
Decapoda	Litopenaeus vannamei_1	Ghaffari et al., 2014	1	
Decapoda	Litopenaeus vannamei_2	hepatopancreas	1	
Decapoda	Litopenaeus vannamei_3	hepatopancreas	1	
Decapoda	Litopenaeus vannamei_4	hemocytes	1	1
Decapoda	Macrobrachium nipponense	NA	1	1
Decapoda	Pacifastacus leniusculus	Brain, HPT, Hemocyte, Hepatopancreas	1	1
Decapoda	Palaemon argentinus	whole organism	0	0
Decapoda	Penaeus monodon_1	hepatopancreas	0	
Decapoda	Penaeus monodon_2	hepatopancreas	0	0
Decapoda	Procambarus clarkii_1	Eyestalk	1	
Decapoda	Procambarus clarkii_2	Eyestalk, brain, hemocytes, gills, testis, ovary, hepatopancreas, heart, green gland, ventralganglia, Y-organ, hypodermis, muscle	1	1
Decapoda	Scylla olivacea	Na	1	1
Decapoda	Scylla paramamosain	gill	0	0
Euphausiacea	Euphausia superba	NA	0	0
Euphausiacea	Meganyctiphanes norvegica	adult	1	1
Isopoda	Asellus aquaticus	NA	0	0
Isopoda	Bragasellus molinai	whole organism	0	0
Isopoda	Bragasellus peltatus	whole organism	0	0
Isopoda	Proasellus aragonensis	whole organism	1	1
Isopoda	Proasellus arthrodilus	whole organism	1	1
Isopoda	Proasellus assaforensis	whole organism	0	0
Isopoda	Proasellus beticus	whole organism	1	1
Isopoda	Proasellus cantabricus	whole organism	0	0
Isopoda	Proasellus cavaticus	whole organism	1	1
Isopoda	Proasellus coiffaiti	whole organism	1	1
Isopoda	Proasellus coxalis	whole organism	1	1
Isopoda	Proasellus ebrensis	whole organism	0	0
Isopoda	Proasellus escolai	whole organism	1	1
Isopoda	Proasellus grafi	whole organism	1	1

Isopoda	Proasellus granadensis	whole organism	1	1
Isopoda	Proasellus hercegovinensis	whole organism	1	1
Isopoda	Proasellus ibericus	whole organism	1	1
Isopoda	Proasellus jaloniacus	whole organism	1	1
Isopoda	Proasellus karamani	whole organism	1	1
Isopoda	Proasellus margalefi	whole organism	0	0
Isopoda	Proasellus meridianus	whole organism	1	1
Isopoda	Proasellus ortizi	whole organism	1	1
Isopoda	Proasellus parvulus	whole organism	1	1
Isopoda	Proasellus racovitzai	whole organism	1	1
Isopoda	Proasellus rectus	whole organism	1	1
Isopoda	Proasellus solanasi	whole organism	1	1
Isopoda	Proasellus spelaeus	whole organism	1	1
Mysida	Neomysis awatschensis	whole organism	1	1
			**Total malacostracan genes**	40

Additional file 5F. Tak1.

Arthropoda

Class (subphylum)	Species	Tissue type	Total gene counts	References
Insecta	Drosophila melanogaster	whole organism	1	Palmer et al., 2015
Insecta	Anopheles gambiae	whole organism	1	ImmunoDB
Insecta	Aedes aegypti	whole organism	1	ImmunoDB
Chilopoda (Myriapoda)	Strigamia maritima	whole organism	3	Palmer et al., 2015
Arachnida (Chelicerata)	Mesobuthus martensii	whole organism	1	Palmer et al., 2015
Arachnida (Chelicerata)	Ixodes scapularis	whole organism	1	Palmer et al., 2015
Branchiopoda (Crustacea)	Daphnia pulex	whole organism	1	Palmer et al., 2015

Malacostraca

Order	Species/Datasets	Tissue type	Total gene counts	Total number of non-redundant genes per species
Amphipoda	Echinogammarus veneris	NA	0	0
Amphipoda	Gammarus chevreuxi	NA	0	0
Amphipoda	Gammarus pulex	NA	0	0
Amphipoda	Hyalella azteca_1	NA	0	
Amphipoda	Hyalella azteca_2	NA	0	
Amphipoda	Hyalella azteca_3	whole organism	1	1
Amphipoda	Melita plumulosa	whole organism	0	0
Amphipoda	Parhyale hawaiensis	whole organism	1	1
Amphipoda	Talitrus saltator	brain	1	1
Decapoda	Astacus astacus	hepatopancreas, ovaries, green glands, abdominal musculature	1	1
Decapoda	Astacus leptodactylus_1	hypodermis; Y organ	1	
Decapoda	Astacus leptodactylus_2	hepatopancreas	1	
Decapoda	Astacus leptodactylus_3	hypodermis, Y organ, hepatopancreas, gills, hematocytes, muscle	1	1
Decapoda	Callinectes sapidus	gill 7	1	1
Decapoda	Cancer borealis	nervous system	1	1
Decapoda	Carcinus maenas	NA	1	1
Decapoda	Cherax quadricarinatus_1	hypodermis and gastrolith disc	0	
Decapoda	Cherax quadricarinatus_2	heart, kidney, liver, nerve, testis	1	
Decapoda	Cherax quadricarinatus_3	heart, kidney, liver, nerve, testis	1	1
Decapoda	Eriocheir sinensis_1	NA	1	
Decapoda	Eriocheir sinensis_2	eyestalk, Y-organ, and hepatopancreas	0	
Decapoda	Eriocheir sinensis_3	hepatopancreas	1	1
Decapoda	Farfantepenaeus aztecus	hepatopancreas	1	1
Decapoda	Homarus americanus	nervous system	1	1
Decapoda	Hyas araneus_1	adult	0	
Decapoda	Hyas araneus_2	gill	1	1
Decapoda	Litopenaeus vannamei_1	Ghaffari et al., 2014	1	
Decapoda	Litopenaeus vannamei_2	hepatopancreas	1	
Decapoda	Litopenaeus vannamei_3	hepatopancreas	1	
Decapoda	Litopenaeus vannamei_4	hemocytes	1	1
Decapoda	Macrobrachium nipponense	NA	1	1
Decapoda	Pacifastacus leniusculus	Brain, HPT, Hemocyte, Hepatopancreas	1	1
Decapoda	Palaemon argentinus	whole organism	0	0
Decapoda	Penaeus monodon_1	hepatopancreas	1	
Decapoda	Penaeus monodon_2	hepatopancreas	1	1
Decapoda	Procambarus clarkii_1	Eyestalk	1	
Decapoda	Procambarus clarkii_2	Eyestalk, brain, hemocytes, gills, testis, ovary, hepatopancreas, heart, green gland, ventralganglia, Y-organ, hypodermis, muscle	1	1
Decapoda	Scylla olivacea	Na	1	1
Decapoda	Scylla paramamosain	gill	0	0
Euphausiacea	Euphausia superba	NA	1	1
Euphausiacea	Meganyctiphanes norvegica	adult	0	0
Isopoda	Asellus aquaticus	NA	0	0
Isopoda	Bragasellus molinai	whole organism	1	1
Isopoda	Bragasellus peltatus	whole organism	1	1
Isopoda	Proasellus aragonensis	whole organism	1	1
Isopoda	Proasellus arthrodilus	whole organism	1	1
Isopoda	Proasellus assaforensis	whole organism	0	0
Isopoda	Proasellus beticus	whole organism	0	0
Isopoda	Proasellus cantabricus	whole organism	1	1
Isopoda	Proasellus cavaticus	whole organism	1	1
Isopoda	Proasellus coiffaiti	whole organism	1	1
Isopoda	Proasellus coxalis	whole organism	1	1
Isopoda	Proasellus ebrensis	whole organism	1	1

Isopoda	Proasellus escolai	whole organism	0	0
Isopoda	Proasellus grafi	whole organism	1	1
Isopoda	Proasellus granadensis	whole organism	0	0
Isopoda	Proasellus hercegovinensis	whole organism	1	1
Isopoda	Proasellus ibericus	whole organism	1	1
Isopoda	Proasellus jaloniacus	whole organism	1	1
Isopoda	Proasellus karamani	whole organism	1	1
Isopoda	Proasellus margalefi	whole organism	0	0
Isopoda	Proasellus meridianus	whole organism	1	1
Isopoda	Proasellus ortizi	whole organism	0	0
Isopoda	Proasellus parvulus	whole organism	1	1
Isopoda	Proasellus racovitzai	whole organism	0	0
Isopoda	Proasellus rectus	whole organism	1	1
Isopoda	Proasellus solanasi	whole organism	1	1
Isopoda	Proasellus spelaeus	whole organism	0	0
Mysida	Neomysis awatschensis	whole organism	1	1
			**Total malacostracan genes**	39
