# Supplementary material for: Comparative genomic analysis of innate immunity reveals novel and conserved components in crustacean food crop species
Source: BMC Genomics. 2017 May 18;18:389. doi: 10.1186/s12864-017-3769-4 (PMC5437397; doi:10.1186/s12864-017-3769-4)
Supplement: Supplementary file 12 — JAK-STAT pathway members in malacostracans. (A) Activation of the JAK-STAT signalling occurs through the binding of ligands such as cytokines to the cytokine receptor Domeless. Conserved protein domains of Domeless are shown in the figure inset. This binding activates the phosphorylation of the Janus kinase (JAK) proteins, which creates docking sites for Signal Transducer and Activator of Transcription (STAT) proteins through their Src Homology 2 (SH2) domains. STATs are phosphorylated by JAKs and activated STATs dimerise and are translocated to the nucleus to induce transcription. JAK-STAT transduction is controlled by suppressors of cytokine signalling (SOCS) and protein inhibitors of activated STAT (PIAS). SOCS proteins inhibit STATs phosphorylation via two mechanisms; 1) by competing with STATs for phosphotyrosine binding sites on cytokine receptors and (2) by binding to JAKs and preventing the recruitment of STATs onto the Domeless receptor. PIAS, also known as the E3 SUMO-protein ligase PIAS, is a transcriptional co-regulator that has the ability to inhibit STAT function. Phylogenetic trees of (B) JAK, (C) STAT and (D) PIAS are constructed using the maximum-likelihood method from an amino acid multiple sequence alignment. Two groups of PIAS proteins have been identified in malacostracans. Taxa labels are depicted as their respective colour codes. Bootstrap support values (n=1000) for all trees can be found in Additional file 14: Figure S8. Scale bar represents substitution per site. (PDF 972 kb) [file 12864_2017_3769_MOESM12_ESM.pdf]

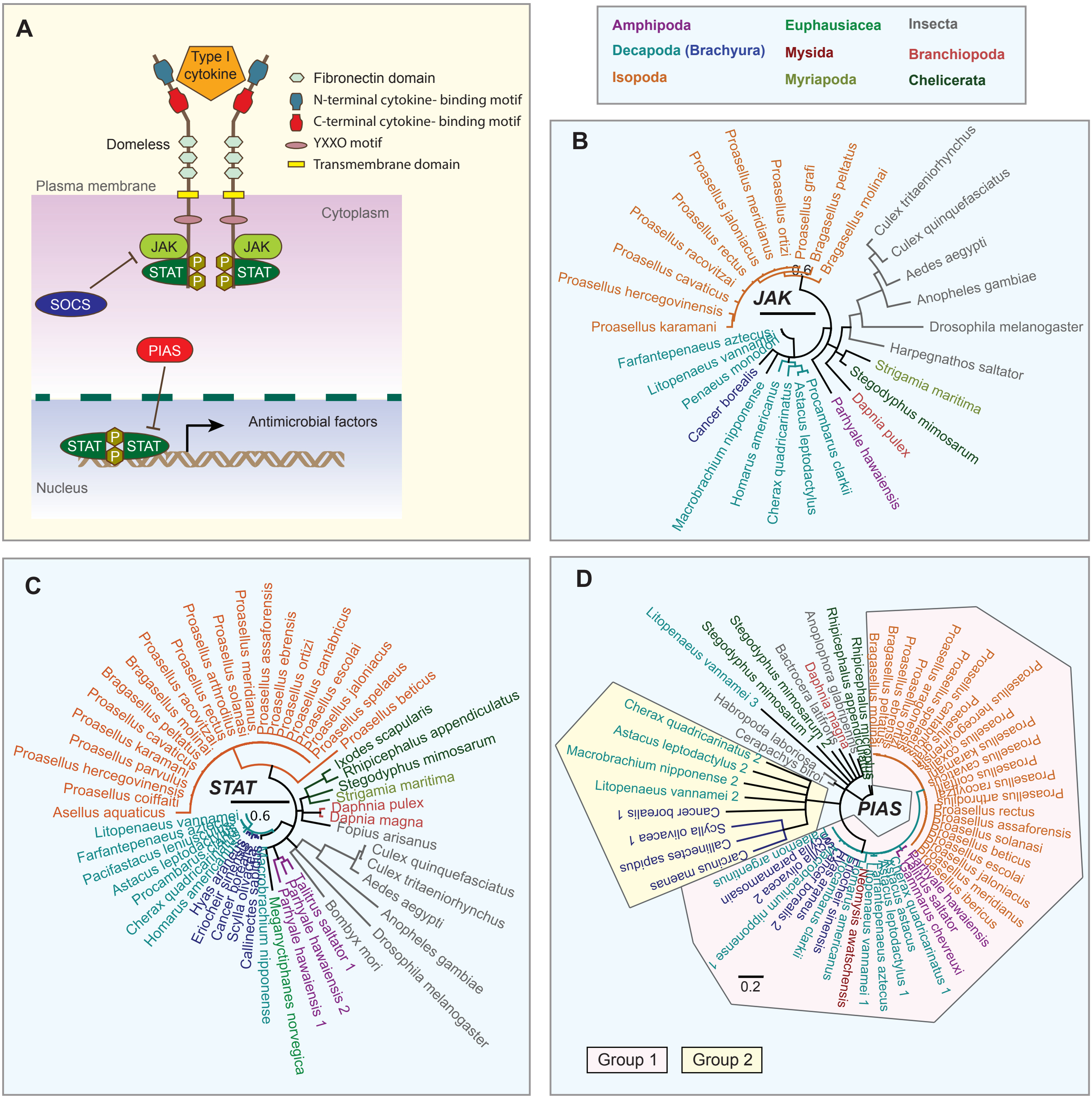

A
Type I cytokine
Fibronectin domain
N-terminal cytokine- binding motif
C-terminal cytokine- binding motif
YXXO motif
Transmembrane domain
Domeless
Plasma membrane
Cytoplasm
JAK
STAT
P
SOCS
PIAS
Antimicrobial factors
Nucleus
Amphipoda
Euphausiacea
Insecta
Decapoda (Brachyura)
Mysida
Branchiopoda
Isopoda
Myriapoda
Chelicerata
B
JAK
0.6
Proasellus jaloniacus
Proasellus meridianus
Proasellus ortizi
Proasellus grafi
Bragasellus peltatus
Bragasellus molinai
Proasellus rectus
Proasellus racovitzai
Proasellus cavaticus
Proasellus hercegovinensis
Proasellus karamani
Culex tritaeniorhynchus
Culex quinquefasciatus
Aedes aegypti
Anopheles gambiae
Drosophila melanogaster
Harpegnathos saltator
Strigamia maritima
Stegodyphus mimosarum
Dapnia pulex
Parhyale hawaiensis
Procambarus clarkii
Astacus leptodactylus
Cherax quadricarinatus
Homarus americanus
Macrobrachium nipponense
Cancer borealis
Penaeus monodon
Litopenaeus vannamei
Farfantepenaeus aztecus
C
STAT
0.6
Proasellus assaforensis
Proasellus meridianus
Proasellus solanasi
Proasellus arthrodilus
Proasellus rectus
Proasellus racovitzai
Bragasellus molinai
Bragasellus peltatus
Proasellus cavaticus
Proasellus karamani
Proasellus parvulus
Proasellus hercegovinensis
Proasellus coiffaiti
Asellus aquaticus
Proasellus ebrensis
Proasellus ortizi
Proasellus cantabricus
Proasellus escolai
Proasellus jaloniacus
Proasellus spelaeus
Proasellus beticus
Ixodes scapularis
Rhipicephalus appendiculatus
Stegodyphus mimosarum
Strigamia maritima
Daphnia pulex
Daphnia magna
Fopius arisanus
Culex quinquefasciatus
Culex tritaeniorhynchus
Aedes aegypti
Anopheles gambiae
Drosophila melanogaster
Bombyx mori
Talitrus saltator 1
Talitrus saltator 2
Parhyale hawaiensis 2
Parhyale hawaiensis 1
Meganyctiphanes norvegica
Macrobrachium nipponense
Callinectes sapidus
Scylla olivacea
Cancer borealis
Eriocheir sinensis
Hyas araneus
Homarus americanus
Cherax quadricarinatus
Procambarus clarkii
Astacus leptodactylus
Pacifastacus leniusculus
Farfantepenaeus aztecus
Litopenaeus vannamei
D
PIAS
0.2
Litopenaeus vannamei 3
Stegodyphus mimosarum 1
Stegodyphus mimosarum 2
Habropoda laboriosa
Cerapachys biroi
Bactrocera latifrons
Anoplophora glabripennis
Daphnia magna
Rhipicephalus appendiculatus
Rhipicephalus microplus
Bragasellus molinai
Bragasellus peltatus
Cherax quadricarinatus 2
Astacus leptodactylus 2
Macrobrachium nipponense 2
Litopenaeus vannamei 2
Cancer borealis 1
Scylla olivacea 1
Callinectes sapidus
Carcinus maenas
Palaemon argentinus
Hyas araneus
Scylla olivacea 2
Cancer borealis 2
Procambarus clarkii
Eriocheir sinensis
Homarus americanus
Neomysis awatschensis
Litopenaeus vannamei 1
Farfantepenaeus aztecus
Astacus leptodactylus
Cherax quadricarinatus 1
Gammarus chevreuxi
Talitrus saltator
Parhyale hawaiensis
Proasellus rectus
Proasellus assaforensis
Proasellus solanasi
Proasellus beticus
Proasellus escolai
Proasellus jaloniacus
Proasellus meridianus
Proasellus ibericus
Group 1
Group 2
