# Supplementary material for: Comparative genomic analysis of innate immunity reveals novel and conserved components in crustacean food crop species
Source: BMC Genomics. 2017 May 18;18:389. doi: 10.1186/s12864-017-3769-4 (PMC5437397; doi:10.1186/s12864-017-3769-4)
Supplement: Supplementary file 13 — Malacostracans JAK-STAT pathway components. (PDF 228 kb) [file 12864_2017_3769_MOESM13_ESM.pdf]

Additional file 6. Malacostracans JAK-STAT pathway components.

Additional file 6A. Janus kinase.

Arthropoda

Class (subphylum)	Species	Tissue type	Total gene counts	References
Insecta	Drosophila melanogaster	whole organism	1	Palmer et al., 2015
Insecta	Anopheles gambiae	whole organism	1	ImmunoDB
Insecta	Aedes aegypti	whole organism	1	ImmunoDB
Chilopoda (Myriapoda)	Strigamia maritima	whole organism	1	Palmer et al., 2015
Arachnida (Chelicerata)	Mesobuthus martensii	whole organism	0	Palmer et al., 2015
Arachnida (Chelicerata)	Ixodes scapularis	whole organism	1	Palmer et al., 2015
Branchiopoda (Crustacea)	Daphnia pulex	whole organism	1	Palmer et al., 2015

Malacostraca

Order	Species/Datasets	Tissue type	Total gene counts	Total number of non-redundant genes per species
Amphipoda	Echinogammarus veneris	NA	0	0
Amphipoda	Gammarus chevreuxi	NA	0	0
Amphipoda	Gammarus pulex	NA	0	0
Amphipoda	Hyalella azteca_1	NA	0	
Amphipoda	Hyalella azteca_2	NA	0	
Amphipoda	Hyalella azteca_3	whole organism	0	0
Amphipoda	Melita plumulosa	whole organism	0	0
Amphipoda	Parhyale hawaiensis	whole organism	1	1
Amphipoda	Talitrus saltator	brain	1	1
Decapoda	Astacus astacus	hepatopancreas, ovaries, green glands, abdominal musculature	0	0
Decapoda	Astacus leptodactylus_1	hypodermis; Y organ	1	
Decapoda	Astacus leptodactylus_2	hepatopancreas	0	
Decapoda	Astacus leptodactylus_3	hypodermis, Y organ, hepatopancreas, gills, hematocytes, muscle	1	1
Decapoda	Callinectes sapidus	gill 7	0	0
Decapoda	Cancer borealis	nervous system	1	1
Decapoda	Carcinus maenas	NA	1	1
Decapoda	Cherax quadricarinatus_1	hypodermis and gastrolith disc	0	
Decapoda	Cherax quadricarinatus_2	heart, kidney, liver, nerve, testis	1	
Decapoda	Cherax quadricarinatus_3	heart, kidney, liver, nerve, testis	1	1
Decapoda	Eriocheir sinensis_1	NA	1	
Decapoda	Eriocheir sinensis_2	eyestalk, Y-organ, and hepatopancreas	1	
Decapoda	Eriocheir sinensis_3	hepatopancreas	0	1
Decapoda	Farfantepenaeus aztecus	hepatopancreas	1	1
Decapoda	Homarus americanus	nervous system	1	1
Decapoda	Hyas araneus_1	adult	0	
Decapoda	Hyas araneus_2	gill	1	1
Decapoda	Litopenaeus vannamei_1	Ghaffari et al., 2014	1	
Decapoda	Litopenaeus vannamei_2	hepatopancreas	1	
Decapoda	Litopenaeus vannamei_3	hepatopancreas	1	
Decapoda	Litopenaeus vannamei_4	hemocytes	1	1
Decapoda	Macrobrachium nipponense	NA	1	1
Decapoda	Pacifastacus leniusculus	Brain, HPT, Hemocyte, Hepatopancreas	1	1
Decapoda	Palaemon argentinus	whole organism	0	0
Decapoda	Penaeus monodon_1	hepatopancreas	1	
Decapoda	Penaeus monodon_2	hepatopancreas	1	1
Decapoda	Procambarus clarkii_1	Eyestalk	1	
Decapoda	Procambarus clarkii_2	Eyestalk, brain, hemocytes, gills, testis, ovary, hepatopancreas, heart, green gland, ventralganglia, Y-organ, hypodermis, muscle	1	1
Decapoda	Scylla olivacea	Na	0	0
Decapoda	Scylla paramamosain	gill	1	1
Euphausiacea	Euphausia superba	NA	0	0
Euphausiacea	Meganyctiphanes norvegica	adult	0	0
Isopoda	Asellus aquaticus	NA	0	0
Isopoda	Bragasellus molinai	whole organism	1	1
Isopoda	Bragasellus peltatus	whole organism	1	1
Isopoda	Proasellus aragonensis	whole organism	0	0
Isopoda	Proasellus arthrodilus	whole organism	1	1
Isopoda	Proasellus assaforensis	whole organism	0	0
Isopoda	Proasellus beticus	whole organism	0	0
Isopoda	Proasellus cantabricus	whole organism	1	1
Isopoda	Proasellus cavaticus	whole organism	1	1
Isopoda	Proasellus coiffaiti	whole organism	1	1

Isopoda	Proasellus coxalis	whole organism	0	0
Isopoda	Proasellus ebrensis	whole organism	1	1
Isopoda	Proasellus escolai	whole organism	0	0
Isopoda	Proasellus grafi	whole organism	1	1
Isopoda	Proasellus granadensis	whole organism	1	1
Isopoda	Proasellus hercegovinensis	whole organism	1	1
Isopoda	Proasellus ibericus	whole organism	1	1
Isopoda	Proasellus jaloniacus	whole organism	1	1
Isopoda	Proasellus karamani	whole organism	1	1
Isopoda	Proasellus margalefi	whole organism	0	0
Isopoda	Proasellus meridianus	whole organism	1	1
Isopoda	Proasellus ortizi	whole organism	1	1
Isopoda	Proasellus parvulus	whole organism	1	1
Isopoda	Proasellus racovitzai	whole organism	1	1
Isopoda	Proasellus rectus	whole organism	1	1
Isopoda	Proasellus solanasi	whole organism	0	0
Isopoda	Proasellus spelaeus	whole organism	0	0
Mysida	Neomysis awatschensis	whole organism	0	0
			**Total malacostracan genes**	34

Additional file 6B. STAT.

Arthropoda

Class (subphylum)	Species	Tissue type	Total gene counts	References
Insecta	Drosophila melanogaster	whole organism	1	Palmer et al., 2015
Insecta	Anopheles gambiae	whole organism	1	ImmunoDB
Insecta	Aedes aegypti	whole organism	1	ImmunoDB
Chilopoda (Myriapoda)	Strigamia maritima	whole organism	1	Palmer et al., 2015
Arachnida (Chelicerata)	Mesobuthus martensii	whole organism	2	Palmer et al., 2015
Arachnida (Chelicerata)	Ixodes scapularis	whole organism	1	Palmer et al., 2015
Branchiopoda (Crustacea)	Daphnia pulex	whole organism	1	Palmer et al., 2015

Malacostraca

Order	Species/Datasets	Tissue type	Total gene counts	Total number of non-redundant genes per species
Amphipoda	Echinogammarus veneris	NA	1	1
Amphipoda	Gammarus chevreuxi	NA	0	0
Amphipoda	Gammarus pulex	NA	0	0
Amphipoda	Hyalella azteca_1	NA	0	
Amphipoda	Hyalella azteca_2	NA	0	
Amphipoda	Hyalella azteca_3	whole organism	2	2
Amphipoda	Melita plumulosa	whole organism	1	1
Amphipoda	Parhyale hawaiensis	whole organism	2	2
Amphipoda	Talitrus saltator	brain	3	3
Decapoda	Astacus astacus	hepatopancreas, ovaries, green glands, abdominal musculature	0	0
Decapoda	Astacus leptodactylus_1	hypodermis; Y organ	1	
Decapoda	Astacus leptodactylus_2	hepatopancreas	1	
Decapoda	Astacus leptodactylus_3	hypodermis, Y organ, hepatopancreas, gills, hematocytes, muscle	1	1
Decapoda	Callinectes sapidus	gill 7	1	1
Decapoda	Cancer borealis	nervous system	1	1
Decapoda	Carcinus maenas	NA	0	0
Decapoda	Cherax quadricarinatus_1	hypodermis and gastrolith disc	0	
Decapoda	Cherax quadricarinatus_2	heart, kidney, liver, nerve, testis	1	
Decapoda	Cherax quadricarinatus_3	heart, kidney, liver, nerve, testis	1	1
Decapoda	Eriocheir sinensis_1	NA	1	
Decapoda	Eriocheir sinensis_2	eyestalk, Y-organ, and hepatopancreas	1	
Decapoda	Eriocheir sinensis_3	hepatopancreas	1	1
Decapoda	Farfantepenaeus aztecus	hepatopancreas	1	1
Decapoda	Homarus americanus	nervous system	1	1
Decapoda	Hyas araneus_1	adult	1	
Decapoda	Hyas araneus_2	gill	1	1
Decapoda	Litopenaeus vannamei_1	Ghaffari et al., 2014	1	
Decapoda	Litopenaeus vannamei_2	hepatopancreas	1	
Decapoda	Litopenaeus vannamei_3	hepatopancreas	1	
Decapoda	Litopenaeus vannamei_4	hemocytes	1	1
Decapoda	Macrobrachium nipponense	NA	1	1
Decapoda	Pacifastacus leniusculus	Brain, HPT, Hemocyte, Hepatopancreas	1	1
Decapoda	Palaemon argentinus	whole organism	1	1
Decapoda	Penaeus monodon_1	hepatopancreas	1	
Decapoda	Penaeus monodon_2	hepatopancreas	1	1
Decapoda	Procambarus clarkii_1	Eyestalk	1	
Decapoda	Procambarus clarkii_2	Eyestalk, brain, hemocytes, gills, testis, ovary, hepatopancreas, heart, green gland, ventralganglia, Y-organ, hypodermis, muscle	1	1
Decapoda	Scylla olivacea	Na	1	1
Decapoda	Scylla paramamosain	gill	0	0
Euphausiacea	Euphausia superba	NA	1	1
Euphausiacea	Meganyctiphanes norvegica	adult	1	1
Isopoda	Asellus aquaticus	NA	1	1
Isopoda	Bragasellus molinai	whole organism	1	1
Isopoda	Bragasellus peltatus	whole organism	1	1
Isopoda	Proasellus aragonensis	whole organism	1	1
Isopoda	Proasellus arthrodilus	whole organism	1	1
Isopoda	Proasellus assaforensis	whole organism	1	1
Isopoda	Proasellus beticus	whole organism	1	1
Isopoda	Proasellus cantabricus	whole organism	1	1
Isopoda	Proasellus cavaticus	whole organism	1	1
Isopoda	Proasellus coiffaiti	whole organism	1	1
Isopoda	Proasellus coxalis	whole organism	1	1

Isopoda	Proasellus ebrensis	whole organism	1	1
Isopoda	Proasellus escolai	whole organism	1	1
Isopoda	Proasellus grafi	whole organism	1	1
Isopoda	Proasellus granadensis	whole organism	0	0
Isopoda	Proasellus hercegovinensis	whole organism	1	1
Isopoda	Proasellus ibericus	whole organism	1	1
Isopoda	Proasellus jaloniacus	whole organism	1	1
Isopoda	Proasellus karamani	whole organism	1	1
Isopoda	Proasellus margalefi	whole organism	1	1
Isopoda	Proasellus meridianus	whole organism	1	1
Isopoda	Proasellus ortizi	whole organism	1	1
Isopoda	Proasellus parvulus	whole organism	1	1
Isopoda	Proasellus racovitzai	whole organism	1	1
Isopoda	Proasellus rectus	whole organism	1	1
Isopoda	Proasellus solanasi	whole organism	1	1
Isopoda	Proasellus spelaeus	whole organism	1	1
Mysida	Neomysis awatschensis	whole organism	0	0
			**Total malacostracan genes**	52

Additional file 6C. PIAS.

Arthropoda

Class (subphylum)	Species	Tissue type	Total gene counts	References
Insecta	Drosophila melanogaster	whole organism	1	proteome
Insecta	Anopheles gambiae	whole organism	1	proteome
Insecta	Aedes aegypti	whole organism	1	proteome
Chilopoda (Myriapoda)	Strigamia maritima	whole organism	1	proteome
Arachnida (Chelicerata)	Mesobuthus martensii	whole organism	0	proteome
Arachnida (Chelicerata)	Ixodes scapularis	whole organism	2	proteome
Branchiopoda (Crustacea)	Daphnia pulex	whole organism	1	proteome

Malacostraca

Order	Species/Datasets	Tissue type	Total gene counts	Total number of non-redundant genes per species
Amphipoda	Echinogammarus veneris	NA	0	0
Amphipoda	Gammarus chevreuxi	NA	1	1
Amphipoda	Gammarus pulex	NA	0	0
Amphipoda	Hyalella azteca_1	NA	1	
Amphipoda	Hyalella azteca_2	NA	1	
Amphipoda	Hyalella azteca_3	whole organism	0	1
Amphipoda	Melita plumulosa	whole organism	1	1
Amphipoda	Parhyale hawaiensis	whole organism	1	1
Amphipoda	Talitrus saltator	brain	1	1
Decapoda	Astacus astacus	hepatopancreas, ovaries, green glands, abdominal musculature	1	1
Decapoda	Astacus leptodactylus_1	hypodermis; Y organ	2	
Decapoda	Astacus leptodactylus_2	hepatopancreas	2	
Decapoda	Astacus leptodactylus_3	hypodermis, Y organ, hepatopancreas, gills, hematocytes, muscle	2	2
Decapoda	Callinectes sapidus	gill 7	1	1
Decapoda	Cancer borealis	nervous system	2	2
Decapoda	Carcinus maenas	NA	1	1
Decapoda	Cherax quadricarinatus_1	hypodermis and gastrolith disc	1	
Decapoda	Cherax quadricarinatus_2	heart, kidney, liver, nerve, testis	2	
Decapoda	Cherax quadricarinatus_3	heart, kidney, liver, nerve, testis	2	2
Decapoda	Eriocheir sinensis_1	NA	1	
Decapoda	Eriocheir sinensis_2	eyestalk, Y-organ, and hepatopancreas	1	
Decapoda	Eriocheir sinensis_3	hepatopancreas	1	1
Decapoda	Farfantepenaeus aztecus	hepatopancreas	1	1
Decapoda	Homarus americanus	nervous system	1	1
Decapoda	Hyas araneus_1	adult	0	
Decapoda	Hyas araneus_2	gill	1	1
Decapoda	Litopenaeus vannamei_1	Ghaffari et al., 2014	3	
Decapoda	Litopenaeus vannamei_2	hepatopancreas	1	
Decapoda	Litopenaeus vannamei_3	hepatopancreas	2	
Decapoda	Litopenaeus vannamei_4	hemocytes	1	3
Decapoda	Macrobrachium nipponense	NA	2	2
Decapoda	Pacifastacus leniusculus	Brain, HPT, Hemocyte, Hepatopancreas	1	1
Decapoda	Palaemon argentinus	whole organism	1	1
Decapoda	Penaeus monodon_1	hepatopancreas	1	
Decapoda	Penaeus monodon_2	hepatopancreas	1	1
Decapoda	Procambarus clarkii_1	Eyestalk	1	
Decapoda	Procambarus clarkii_2	Eyestalk, brain, hemocytes, gills, testis, ovary, hepatopancreas, heart, green gland, ventralganglia, Y-organ, hypodermis, muscle	1	1
Decapoda	Scylla olivacea	Na	2	2
Decapoda	Scylla paramamosain	gill	0	0
Euphausiacea	Euphausia superba	NA	1	1
Euphausiacea	Meganyctiphanes norvegica	adult	0	0
Isopoda	Asellus aquaticus	NA	1	1
Isopoda	Bragasellus molinai	whole organism	1	1
Isopoda	Bragasellus peltatus	whole organism	1	1
Isopoda	Proasellus aragonensis	whole organism	1	1
Isopoda	Proasellus arthrodilus	whole organism	1	1
Isopoda	Proasellus assaforensis	whole organism	1	1
Isopoda	Proasellus beticus	whole organism	1	1
Isopoda	Proasellus cantabricus	whole organism	1	1
Isopoda	Proasellus cavaticus	whole organism	1	1
Isopoda	Proasellus coiffaiti	whole organism	1	1
Isopoda	Proasellus coxalis	whole organism	1	1
Isopoda	Proasellus ebrensis	whole organism	1	1
Isopoda	Proasellus escolai	whole organism	1	1
Isopoda	Proasellus grafi	whole organism	1	1

Isopoda	Proasellus granadensis	whole organism	1	1
Isopoda	Proasellus hercegovinensis	whole organism	1	1
Isopoda	Proasellus ibericus	whole organism	1	1
Isopoda	Proasellus jaloniacus	whole organism	1	1
Isopoda	Proasellus karamani	whole organism	1	1
Isopoda	Proasellus margalefi	whole organism	1	1
Isopoda	Proasellus meridianus	whole organism	1	1
Isopoda	Proasellus ortizi	whole organism	1	1
Isopoda	Proasellus parvulus	whole organism	0	0
Isopoda	Proasellus racovitzai	whole organism	1	1
Isopoda	Proasellus rectus	whole organism	1	1
Isopoda	Proasellus solanasi	whole organism	1	1
Isopoda	Proasellus spelaeus	whole organism	1	1
Mysida	Neomysis awatschensis	whole organism	1	1
			**Total malacostracan genes**	57

Additional file 6D. SOCS.

Arthropoda

Class (subphylum)	Species	Tissue type	Total gene counts	References
Insecta	Drosophila melanogaster	whole organism	Uniprot	Uniprot
Insecta	Anopheles gambiae	whole organism	Uniprot	Uniprot
Insecta	Aedes aegypti	whole organism	Uniprot	Uniprot
Chilopoda (Myriapoda)	Strigamia maritima	whole organism	Uniprot	Uniprot
Arachnida (Chelicerata)	Mesobuthus martensii	whole organism	2	proteome
Arachnida (Chelicerata)	Ixodes scapularis	whole organism	3	proteome
Branchiopoda (Crustacea)	Daphnia pulex	whole organism	3	proteome

Malacostraca

Order	Species/Datasets	Tissue type	Total gene counts	Total number of non-redundant genes per species
Amphipoda	Echinogammarus veneris	NA	1	1
Amphipoda	Gammarus chevreuxi	NA	3	3
Amphipoda	Gammarus pulex	NA	0	0
Amphipoda	Hyalella azteca_1	NA	1	
Amphipoda	Hyalella azteca_2	NA	2	
Amphipoda	Hyalella azteca_3	whole organism	2	2
Amphipoda	Melita plumulosa	whole organism	1	1
Amphipoda	Parhyale hawaiensis	whole organism	6	6
Amphipoda	Talitrus saltator	brain	3	3
Decapoda	Astacus astacus	hepatopancreas, ovaries, green glands, abdominal musculature	4	4
Decapoda	Astacus leptodactylus_1	hypodermis; Y organ	5	
Decapoda	Astacus leptodactylus_2	hepatopancreas	2	
Decapoda	Astacus leptodactylus_3	hypodermis, Y organ, hepatopancreas, gills, hematocytes, muscle	5	5
Decapoda	Callinectes sapidus	gill 7	3	3
Decapoda	Cancer borealis	nervous system	5	5
Decapoda	Carcinus maenas	NA	4	4
Decapoda	Cherax quadricarinatus_1	hypodermis and gastrolith disc	0	
Decapoda	Cherax quadricarinatus_2	heart, kidney, liver, nerve, testis	4	
Decapoda	Cherax quadricarinatus_3	heart, kidney, liver, nerve, testis	4	4
Decapoda	Eriocheir sinensis_1	NA	4	
Decapoda	Eriocheir sinensis_2	eyestalk, Y-organ, and hepatopancreas	5	
Decapoda	Eriocheir sinensis_3	hepatopancreas	3	5
Decapoda	Farfantepenaeus aztecus	hepatopancreas	4	4
Decapoda	Homarus americanus	nervous system	5	5
Decapoda	Hyas araneus_1	adult	1	
Decapoda	Hyas araneus_2	gill	5	5
Decapoda	Litopenaeus vannamei_1	Ghaffari et al., 2014	6	
Decapoda	Litopenaeus vannamei_2	hepatopancreas	5	
Decapoda	Litopenaeus vannamei_3	hepatopancreas	5	
Decapoda	Litopenaeus vannamei_4	hemocytes	3	6
Decapoda	Macrobrachium nipponense	NA	5	5
Decapoda	Pacifastacus leniusculus	Brain, HPT, Hemocyte, Hepatopancreas	2	2
Decapoda	Palaemon argentinus	whole organism	1	1
Decapoda	Penaeus monodon_1	hepatopancreas	2	
Decapoda	Penaeus monodon_2	hepatopancreas	2	2
Decapoda	Procambarus clarkii_1	Eyestalk	5	
Decapoda	Procambarus clarkii_2	Eyestalk, brain, hemocytes, gills, testis, ovary, hepatopancreas, heart, green gland, ventralganglia, Y-organ, hypodermis, muscle	5	5
Decapoda	Scylla olivacea	Na	5	5
Decapoda	Scylla paramamosain	gill	2	2
Euphausiacea	Euphausia superba	NA	1	1
Euphausiacea	Meganyctiphanes norvegica	adult	1	1
Isopoda	Asellus aquaticus	NA	0	0
Isopoda	Bragasellus molinai	whole organism	4	4
Isopoda	Bragasellus peltatus	whole organism	4	4
Isopoda	Proasellus aragonensis	whole organism	3	3
Isopoda	Proasellus arthrodilus	whole organism	4	4
Isopoda	Proasellus assaforensis	whole organism	4	4
Isopoda	Proasellus beticus	whole organism	3	3
Isopoda	Proasellus cantabricus	whole organism	4	4
Isopoda	Proasellus cavaticus	whole organism	5	5
Isopoda	Proasellus coiffaiti	whole organism	1	1
Isopoda	Proasellus coxalis	whole organism	5	5
Isopoda	Proasellus ebrensis	whole organism	5	5

Isopoda	Proasellus escolai	whole organism	1	1
Isopoda	Proasellus grafi	whole organism	4	4
Isopoda	Proasellus granadensis	whole organism	2	2
Isopoda	Proasellus hercegovinensis	whole organism	3	3
Isopoda	Proasellus ibericus	whole organism	3	3
Isopoda	Proasellus jaloniacus	whole organism	4	4
Isopoda	Proasellus karamani	whole organism	4	4
Isopoda	Proasellus margalefi	whole organism	1	1
Isopoda	Proasellus meridianus	whole organism	5	5
Isopoda	Proasellus ortizi	whole organism	4	4
Isopoda	Proasellus parvulus	whole organism	4	4
Isopoda	Proasellus racovitzai	whole organism	4	4
Isopoda	Proasellus rectus	whole organism	5	5
Isopoda	Proasellus solanasi	whole organism	4	4
Isopoda	Proasellus spelaeus	whole organism	3	3
Mysida	Neomysis awatschensis	whole organism	0	0
			**Total malacostracan genes**	183
