# Supplementary material for: Comparative genomic analysis of innate immunity reveals novel and conserved components in crustacean food crop species
Source: BMC Genomics. 2017 May 18;18:389. doi: 10.1186/s12864-017-3769-4 (PMC5437397; doi:10.1186/s12864-017-3769-4)
Supplement: Supplementary file 15 — Malacostracans antimicrobial peptides. (PDF 107 kb) [file 12864_2017_3769_MOESM15_ESM.pdf]

Additional file 7. Malacostracans antimicrobial peptides.

Additional file 7A. Antilipopolysaccharide factors.

Arthropoda

Class (subphylum)	Species	Tissue type	Total gene counts	References
Insecta	Drosophila melanogaster	whole organism	0	proteome
Insecta	Anopheles gambiae	whole organism	0	proteome
Insecta	Aedes aegypti	whole organism	0	proteome
Chilopoda (Myriapoda)	Strigamia maritima	whole organism	0	proteome
Arachnida (Chelicerata)	Mesobuthus martensii	whole organism	0	proteome
Arachnida (Chelicerata)	Ixodes scapularis	whole organism	0	proteome
Branchiopoda (Crustacea)	Daphnia pulex	whole organism	0	proteome

Malacostraca

Order	Species/Datasets	Tissue type	Total gene counts	Total number of non-redundant genes per species
Amphipoda	Echinogammarus veneris	NA	1	1
Amphipoda	Gammarus pulex	NA	4	4
Amphipoda	Hyalella azteca_1	NA	3	
Amphipoda	Hyalella azteca_2	NA	4	
Amphipoda	Hyalella azteca_3	whole organism	3	4
Amphipoda	Melita plumulosa	whole organism	2	2
Amphipoda	Parhyale hawaiensis	whole organism	8	8
Amphipoda	Talitrus saltator	brain	6	6
Decapoda	Astacus astacus	hepatopancreas, ovaries, green glands, abdominal musculature	7	7
Decapoda	Astacus leptodactylus_1	hypodermis; Y organ	7	
Decapoda	Astacus leptodactylus_2	hepatopancreas	9	
Decapoda	Astacus leptodactylus_3	hypodermis, Y organ, hepatopancreas, gills, hematocytes, muscle	9	13
Decapoda	Cherax quadricarinatus_1	hypodermis and gastrolith disc	11	
Decapoda	Cherax quadricarinatus_2	heart, kidney, liver, nerve, testis	9	
Decapoda	Cherax quadricarinatus_3	heart, kidney, liver, nerve, testis	12	14
Decapoda	Farfantepenaeus aztecus	hepatopancreas	7	7
Decapoda	Homarus americanus	nervous system	4	4
Decapoda	Litopenaeus vannamei_1	Ghaffari et al., 2014	8	
Decapoda	Litopenaeus vannamei_2	hepatopancreas	5	
Decapoda	Litopenaeus vannamei_3	hepatopancreas	6	
Decapoda	Litopenaeus vannamei_4	hemocytes	5	9
Decapoda	Pacifastacus leniusculus	Brain, HPT, Hemocyte, Hepatopancreas	5	5
Decapoda	Penaeus monodon_1	hepatopancreas	17	
Decapoda	Penaeus monodon_2	hepatopancreas	17	8
Decapoda	Procambarus clarkii_1	Eyestalk	12	
Decapoda	Procambarus clarkii_2	Eyestalk, brain, hemocytes, gills, testis, ovary, hepatopancreas, heart, green gland, ventralganglia, Y-organ, hypodermis, muscle	7	13
Decapoda	Callinectes sapidus	gill 7	5	5
Decapoda	Cancer borealis	nervous system	1	1
Decapoda	Carcinus maenas	NA	3	3
Decapoda	Eriocheir sinensis_1	NA	4	
Decapoda	Eriocheir sinensis_2	eyestalk, Y-organ, and hepatopancreas	2	
Decapoda	Eriocheir sinensis_3	hepatopancreas	5	6
Decapoda	Hyas araneus_1	adult	28	
Decapoda	Hyas araneus_2	gill	6	20
Decapoda	Scylla olivacea	Na	6	6
Decapoda	Scylla paramamosain	gill	8	8
Decapoda	Macrobrachium nipponense	NA	5	5
Decapoda	Palaemon argentinus	whole organism	4	4
Euphausiacea	Euphausia superba	NA	8	8
Euphausiacea	Meganyctiphanes norvegica	adult	2	2
Isopoda	Asellus aquaticus	NA	3	3
Isopoda	Bragasellus molinai	whole organism	4	4
Isopoda	Bragasellus peltatus	whole organism	5	5
Isopoda	Proasellus aragonensis	whole organism	7	7
Isopoda	Proasellus arthrodilus	whole organism	8	8
Isopoda	Proasellus assaforensis	whole organism	7	7
Isopoda	Proasellus beticus	whole organism	4	4
Isopoda	Proasellus cantabricus	whole organism	6	6
Isopoda	Proasellus cavaticus	whole organism	7	7
Isopoda	Proasellus coiffaiti	whole organism	8	8
Isopoda	Proasellus coxalis	whole organism	8	8

Isopoda	Proasellus ebrensis	whole organism	5	5
Isopoda	Proasellus escolai	whole organism	4	4
Isopoda	Proasellus grafi	whole organism	6	6
Isopoda	Proasellus granadensis	whole organism	4	4
Isopoda	Proasellus hercegovinensis	whole organism	3	3
Isopoda	Proasellus ibericus	whole organism	6	6
Isopoda	Proasellus jaloniacus	whole organism	5	5
Isopoda	Proasellus karamani	whole organism	8	8
Isopoda	Proasellus margalefi	whole organism	4	4
Isopoda	Proasellus meridianus	whole organism	6	6
Isopoda	Proasellus ortizi	whole organism	7	7
Isopoda	Proasellus parvulus	whole organism	4	4
Isopoda	Proasellus racovitzai	whole organism	5	5
Isopoda	Proasellus rectus	whole organism	7	7
Isopoda	Proasellus solanasi	whole organism	7	7
Isopoda	Proasellus spelaeus	whole organism	6	6
Mysida	Neomysis awatschensis	whole organism	10	10
			**Total malacostracan genes**	337

Additional file 7B. Crustins.
Arthropoda

Class (subphylum)	Species	Tissue type	Total gene counts	References
Insecta	Drosophila melanogaster	whole organism	0	proteome
Insecta	Anopheles gambiae	whole organism	0	proteome
Insecta	Aedes aegypti	whole organism	0	proteome
Chilopoda (Myriapoda)	Strigamia maritima	whole organism	0	proteome
Arachnida (Chelicerata)	Mesobuthus martensii	whole organism	0	proteome
Arachnida (Chelicerata)	Ixodes scapularis	whole organism	0	proteome
Branchiopoda (Crustacea)	Daphnia pulex	whole organism	0	proteome

Malacostraca

Order	Species/Datasets	Tissue type	Total gene counts	Total number of non-redundant genes per species
Amphipoda	Echinogammarus veneris	NA	11	11
Amphipoda	Gammarus chevreuxi	NA	13	13
Amphipoda	Gammarus pulex	NA	2	2
Amphipoda	Hyalella azteca_1	NA	4	
Amphipoda	Hyalella azteca_2	NA	5	
Amphipoda	Hyalella azteca_3	whole organism	8	11
Amphipoda	Melita plumulosa	whole organism	7	7
Amphipoda	Parhyale hawaiensis	whole organism	9	9
Amphipoda	Talitrus saltator	brain	8	8
Decapoda	Astacus astacus	hepatopancreas, ovaries, green glands, abdominal musculature	7	7
Decapoda	Astacus leptodactylus_1	hypodermis; Y organ	11	
Decapoda	Astacus leptodactylus_2	hepatopancreas	7	
Decapoda	Astacus leptodactylus_3	hypodermis, Y organ, hepatopancreas, gills, hematocytes, muscle	11	18
Decapoda	Callinectes sapidus	gill 7	5	5
Decapoda	Cancer borealis	nervous system	4	4
Decapoda	Carcinus maenas	NA	7	7
Decapoda	Cherax quadricarinatus_1	hypodermis and gastrolith disc	10	
Decapoda	Cherax quadricarinatus_2	heart, kidney, liver, nerve, testis	7	
Decapoda	Cherax quadricarinatus_3	heart, kidney, liver, nerve, testis	9	12
Decapoda	Eriocheir sinensis_1	NA	9	
Decapoda	Eriocheir sinensis_2	eyestalk, Y-organ, and hepatopancreas	2	
Decapoda	Eriocheir sinensis_3	hepatopancreas	5	10
Decapoda	Farfantepenaeus aztecus	hepatopancreas	2	2
Decapoda	Homarus americanus	nervous system	4	4
Decapoda	Hyas araneus_1	adult	13	
Decapoda	Hyas araneus_2	gill	6	9
Decapoda	Litopenaeus vannamei_1	Ghaffari et al., 2014	16	
Decapoda	Litopenaeus vannamei_2	hepatopancreas	3	
Decapoda	Litopenaeus vannamei_3	hepatopancreas	4	
Decapoda	Litopenaeus vannamei_4	hemocytes	5	15
Decapoda	Macrobrachium nipponense	NA	11	11
Decapoda	Pacifastacus leniusculus	Brain, HPT, Hemocyte, Hepatopancreas	5	5
Decapoda	Palaemon argentinus	whole organism	5	5
Decapoda	Penaeus monodon_1	hepatopancreas	7	
Decapoda	Penaeus monodon_2	hepatopancreas	7	4
Decapoda	Procambarus clarkii_1	Eyestalk	9	
Decapoda	Procambarus clarkii_2	Eyestalk, brain, hemocytes, gills, testis, ovary, hepatopancreas, heart, green gland, ventralganglia, Y-organ, hypodermis, muscle	10	13
Decapoda	Scylla olivacea	Na	4	4
Decapoda	Scylla paramamosain	gill	9	9
Euphausiacea	Euphausia superba	NA	50	50
Euphausiacea	Meganyctiphanes norvegica	adult	14	14
Isopoda	Asellus aquaticus	NA	2	2
Isopoda	Bragasellus molinai	whole organism	8	8
Isopoda	Bragasellus peltatus	whole organism	8	8
Isopoda	Proasellus aragonensis	whole organism	9	9
Isopoda	Proasellus arthrodilus	whole organism	14	14
Isopoda	Proasellus assaforensis	whole organism	13	13
Isopoda	Proasellus beticus	whole organism	3	3
Isopoda	Proasellus cantabricus	whole organism	9	9
Isopoda	Proasellus cavaticus	whole organism	11	11
Isopoda	Proasellus coiffaiti	whole organism	10	10
Isopoda	Proasellus coxalis	whole organism	13	13
Isopoda	Proasellus ebrensis	whole organism	8	8

Isopoda	Proasellus escolai	whole organism	9	9
Isopoda	Proasellus grafi	whole organism	9	9
Isopoda	Proasellus granadensis	whole organism	8	8
Isopoda	Proasellus hercegovinensis	whole organism	6	6
Isopoda	Proasellus ibericus	whole organism	9	9
Isopoda	Proasellus jaloniacus	whole organism	9	9
Isopoda	Proasellus karamani	whole organism	9	9
Isopoda	Proasellus margalefi	whole organism	7	7
Isopoda	Proasellus meridianus	whole organism	8	8
Isopoda	Proasellus ortizi	whole organism	7	7
Isopoda	Proasellus parvulus	whole organism	6	6
Isopoda	Proasellus racovitzai	whole organism	11	11
Isopoda	Proasellus rectus	whole organism	10	10
Isopoda	Proasellus solanasi	whole organism	10	10
Isopoda	Proasellus spelaeus	whole organism	9	9
Mysida	Neomysis awatschensis	whole organism	9	9
			**Total malacostracan genes**	513
