# Supplementary material for: Comparative genomic analysis of innate immunity reveals novel and conserved components in crustacean food crop species
Source: BMC Genomics. 2017 May 18;18:389. doi: 10.1186/s12864-017-3769-4 (PMC5437397; doi:10.1186/s12864-017-3769-4)
Supplement: Supplementary file 16 — Malacostracans antiviral RNAi pathway genes. (PDF 378 kb) [file 12864_2017_3769_MOESM16_ESM.pdf]

Additional file 8. Malacostracans antiviral RNAi pathway genes.

Additional file 8A. Dicer.
Arthropoda

Class (subphylum)	Species	Tissue type	Total gene counts	References
Insecta	Drosophila melanogaster	whole organism	2	ImmunoDB
Insecta	Anopheles gambiae	whole organism	2	ImmunoDB
Insecta	Aedes aegypti	whole organism	2	ImmunoDB
Chilopoda (Myriapoda)	Strigamia maritima	whole organism	1	Palmer et al., 2015
Arachnida (Chelicerata)	Mesobuthus martensii	whole organism	0	Palmer et al., 2015
Arachnida (Chelicerata)	Ixodes scapularis	whole organism	1	Palmer et al., 2015
Branchiopoda (Crustacea)	Daphnia pulex	whole organism	3	McTaggart et al., 2009

Malacostraca

Order	Species/Datasets	Tissue type	Dicer 1	Dicer 2
Amphipoda	Echinogammarus veneris	NA	0	0
Amphipoda	Gammarus chevreuxi	NA	0	0
Amphipoda	Gammarus pulex	NA	0	2
Amphipoda	Hyalella azteca_1	NA	0	0
Amphipoda	Hyalella azteca_2	NA	0	0
Amphipoda	Hyalella azteca_3	whole organism	1	1
Amphipoda	Melita plumulosa	whole organism	1	0
Amphipoda	Parhyale hawaiensis	whole organism	1	1
Amphipoda	Talitrus saltator	brain	1	1
Decapoda	Astacus astacus	hepatopancreas, ovaries, green glands, abdominal musculature	0	1
Decapoda	Astacus leptodactylus_1	hypodermis; Y organ	1	1
Decapoda	Astacus leptodactylus_2	hepatopancreas	0	0
Decapoda	Astacus leptodactylus_3	hypodermis, Y organ, hepatopancreas, gills, hematocytes, muscle	1	1
Decapoda	Callinectes sapidus	gill 7	1	0
Decapoda	Cancer borealis	nervous system	1	1
Decapoda	Carcinus maenas	NA	1	0
Decapoda	Cherax quadricarinatus_1	hypodermis and gastrolith disc	0	1
Decapoda	Cherax quadricarinatus_2	heart, kidney, liver, nerve, testis	1	1
Decapoda	Cherax quadricarinatus_3	heart, kidney, liver, nerve, testis	1	1
Decapoda	Eriocheir sinensis_1	NA	1	1
Decapoda	Eriocheir sinensis_2	eyestalk, Y-organ, and hepatopancreas	1	1
Decapoda	Eriocheir sinensis_3	hepatopancreas	1	1
Decapoda	Farfantepenaeus aztecus	hepatopancreas	1	1
Decapoda	Homarus americanus	nervous system	1	1
Decapoda	Hyas araneus_1	adult	1	1
Decapoda	Hyas araneus_2	gill	1	1
Decapoda	Litopenaeus vannamei_1	Ghaffari et al., 2014	1	1
Decapoda	Litopenaeus vannamei_2	hepatopancreas	1	1
Decapoda	Litopenaeus vannamei_3	hepatopancreas	1	1
Decapoda	Litopenaeus vannamei_4	hemocytes	1	1
Decapoda	Macrobrachium nipponense	NA	1	1
Decapoda	Pacifastacus leniusculus	Brain, HPT, Hemocyte, Hepatopancreas	1	1
Decapoda	Palaemon argentinus	whole organism	0	0
Decapoda	Penaeus monodon_1	hepatopancreas	1	1
Decapoda	Penaeus monodon_2	hepatopancreas	1	1
Decapoda	Procambarus clarkii_1	Eyestalk	1	1
Decapoda	Procambarus clarkii_2	Eyestalk, brain, hemocytes, gills, testis, ovary, hepatopancreas, heart, green gland, ventralganglia, Y-organ, hypodermis, muscle	1	2
Decapoda	Scylla olivacea	Na	1	1
Decapoda	Scylla paramamosain	gill	0	1
Euphausiacea	Euphausia superba	NA	0	3
Euphausiacea	Meganyctiphanes norvegica	adult	0	2
Isopoda	Asellus aquaticus	NA	0	1
Isopoda	Bragasellus molinai	whole organism	1	1
Isopoda	Bragasellus peltatus	whole organism	1	1
Isopoda	Proasellus aragonensis	whole organism	1	0
Isopoda	Proasellus arthrodilus	whole organism	1	0
Isopoda	Proasellus assaforensis	whole organism	1	1
Isopoda	Proasellus beticus	whole organism	1	1
Isopoda	Proasellus cantabricus	whole organism	1	1
Isopoda	Proasellus cavaticus	whole organism	1	1
Isopoda	Proasellus coiffaiti	whole organism	1	1
Isopoda	Proasellus coxalis	whole organism	1	1
Isopoda	Proasellus ebrensis	whole organism	1	1
Isopoda	Proasellus escolai	whole organism	1	1
Isopoda	Proasellus grafi	whole organism	1	1

Isopoda	Proasellus granadensis	whole organism	0	2
Isopoda	Proasellus hercegovinensis	whole organism	1	2
Isopoda	Proasellus ibericus	whole organism	2	0
Isopoda	Proasellus jaloniacus	whole organism	1	1
Isopoda	Proasellus karamani	whole organism	0	2
Isopoda	Proasellus margalefi	whole organism	1	1
Isopoda	Proasellus meridianus	whole organism	1	1
Isopoda	Proasellus ortizi	whole organism	1	1
Isopoda	Proasellus parvulus	whole organism	1	1
Isopoda	Proasellus racovitzai	whole organism	1	1
Isopoda	Proasellus rectus	whole organism	2	0
Isopoda	Proasellus solanasi	whole organism	2	0
Isopoda	Proasellus spelaeus	whole organism	1	1
Mysida	Neomysis awatschensis	whole organism	0	0

	Non redundant gene counts	
	**Dicer 1**	**Dicer 2**
Echinogammarus ve	0	0
Gammarus chevreux	0	0
Gammarus pulex	0	2
Hyalella azteca	1	1
Melita plumulosa	1	0
Parhyale hawaiensis	1	1
Talitrus saltator	1	1
Astacus astacus	0	1
Astacus leptodactylu	1	1
Callinectes sapidus	1	0
Cancer borealis	1	1
Carcinus maenas	1	0
Cherax quadricarina	1	1
Eriocheir sinensis	1	1
Farfantepenaeus azt	1	1
Homarus americanu	1	1
Hyas araneus	1	1
Litopenaeus vannam	1	1
Macrobrachium nipp	1	1
Pacifastacus leniuscu	1	1
Palaemon argentinu	0	0
Penaeus monodon	1	1
Procambarus clarkii	1	1
Scylla olivacea	1	1
Scylla paramamosair	0	1
Euphausia superba	0	3
Meganyctiphanes no	0	2
Asellus aquaticus	0	1
Bragasellus molinai	1	1
Bragasellus peltatus	1	1
Proasellus aragonen	1	0
Proasellus arthrodilu	1	0
Proasellus assaforen	1	1
Proasellus beticus	1	1
Proasellus cantabric	1	1
Proasellus cavaticus	1	1
Proasellus coiffaiti	1	1
Proasellus coxalis	1	1
Proasellus ebrensis	1	1
Proasellus escolai	1	1
Proasellus grafi	1	1
Proasellus granaden	0	2
Proasellus hercegovi	1	2
Proasellus ibericus	2	0
Proasellus jaloniacus	1	1
Proasellus karamani	0	2
Proasellus margalefi	1	1
Proasellus meridianu	1	1
Proasellus ortizi	1	1
Proasellus parvulus	1	1
Proasellus racovitzai	1	1
Proasellus rectus	2	0
Proasellus solanasi	2	0
Proasellus spelaeus	1	1
Neomysis awatscher	0	0

**Total malacostracan**	46	50

Additional file 8B. Drosha.
Arthropoda

Class (subphylum)	Species	Tissue type	Total gene counts	References
Insecta	Drosophila melanogaster	whole organism	1	ImmunoDB
Insecta	Anopheles gambiae	whole organism	1	ImmunoDB
Insecta	Aedes aegypti	whole organism	1	ImmunoDB
Chilopoda (Myriapoda)	Strigamia maritima	whole organism	1	proteome
Arachnida (Chelicerata)	Mesobuthus martensii	whole organism	1	proteome
Arachnida (Chelicerata)	Ixodes scapularis	whole organism	1	Uniprot
Branchiopoda (Crustacea)	Daphnia pulex	whole organism	1	proteome

Malacostraca

Order	Species/Datasets	Tissue type	Total gene counts	Total number of non-redundant genes per species
Amphipoda	Echinogammarus veneris	NA	0	0
Amphipoda	Gammarus chevreuxi	NA	0	0
Amphipoda	Gammarus pulex	NA	0	0
Amphipoda	Hyalella azteca_1	NA	0	
Amphipoda	Hyalella azteca_2	NA	0	
Amphipoda	Hyalella azteca_3	whole organism	1	1
Amphipoda	Melita plumulosa	whole organism	0	0
Amphipoda	Parhyale hawaiensis	whole organism	1	1
Amphipoda	Talitrus saltator	brain	1	1
Decapoda	Astacus astacus	hepatopancreas, ovaries, green glands, abdominal musculature	1	1
Decapoda	Astacus leptodactylus_1	hypodermis; Y organ	1	
Decapoda	Astacus leptodactylus_2	hepatopancreas	1	
Decapoda	Astacus leptodactylus_3	hypodermis, Y organ, hepatopancreas, gills, hematocytes, muscle	1	1
Decapoda	Callinectes sapidus	gill 7	1	1
Decapoda	Cancer borealis	nervous system	1	1
Decapoda	Carcinus maenas	NA	0	0
Decapoda	Cherax quadricarinatus_1	hypodermis and gastrolith disc	0	
Decapoda	Cherax quadricarinatus_2	heart, kidney, liver, nerve, testis	1	
Decapoda	Cherax quadricarinatus_3	heart, kidney, liver, nerve, testis	1	1
Decapoda	Eriocheir sinensis_1	NA	1	
Decapoda	Eriocheir sinensis_2	eyestalk, Y-organ, and hepatopancreas	1	
Decapoda	Eriocheir sinensis_3	hepatopancreas	0	1
Decapoda	Farfantepenaeus aztecus	hepatopancreas	1	1
Decapoda	Homarus americanus	nervous system	1	1
Decapoda	Hyas araneus_1	adult	0	
Decapoda	Hyas araneus_2	gill	1	1
Decapoda	Litopenaeus vannamei_1	Ghaffari et al., 2014	1	
Decapoda	Litopenaeus vannamei_2	hepatopancreas	1	
Decapoda	Litopenaeus vannamei_3	hepatopancreas	1	
Decapoda	Litopenaeus vannamei_4	hemocytes	1	1
Decapoda	Macrobrachium nipponense	NA	1	1
Decapoda	Pacifastacus leniusculus	Brain, HPT, Hemocyte, Hepatopancreas	1	1
Decapoda	Palaemon argentinus	whole organism	0	0
Decapoda	Penaeus monodon_1	hepatopancreas	1	
Decapoda	Penaeus monodon_2	hepatopancreas	1	1
Decapoda	Procambarus clarkii_1	Eyestalk	1	
Decapoda	Procambarus clarkii_2	Eyestalk, brain, hemocytes, gills, testis, ovary, hepatopancreas, heart, green gland, ventralganglia, Y-organ, hypodermis, muscle	1	1
Decapoda	Scylla olivacea	Na	1	1
Decapoda	Scylla paramamosain	gill	0	0
Euphausiacea	Euphausia superba	NA	0	0
Euphausiacea	Meganyctiphanes norvegica	adult	1	1
Isopoda	Asellus aquaticus	NA	0	0
Isopoda	Bragasellus molinai	whole organism	1	1
Isopoda	Bragasellus peltatus	whole organism	1	1
Isopoda	Proasellus aragonensis	whole organism	0	0
Isopoda	Proasellus arthrodilus	whole organism	1	1
Isopoda	Proasellus assaforensis	whole organism	1	1
Isopoda	Proasellus beticus	whole organism	1	1
Isopoda	Proasellus cantabricus	whole organism	1	1
Isopoda	Proasellus cavaticus	whole organism	1	1
Isopoda	Proasellus coiffaiti	whole organism	1	1
Isopoda	Proasellus coxalis	whole organism	1	1
Isopoda	Proasellus ebrensis	whole organism	1	1

Isopoda	Proasellus escolai	whole organism	0	0
Isopoda	Proasellus grafi	whole organism	1	1
Isopoda	Proasellus granadensis	whole organism	1	1
Isopoda	Proasellus hercegovinensis	whole organism	1	1
Isopoda	Proasellus ibericus	whole organism	1	1
Isopoda	Proasellus jaloniacus	whole organism	1	1
Isopoda	Proasellus karamani	whole organism	0	0
Isopoda	Proasellus margalefi	whole organism	1	1
Isopoda	Proasellus meridianus	whole organism	0	0
Isopoda	Proasellus ortizi	whole organism	0	0
Isopoda	Proasellus parvulus	whole organism	1	1
Isopoda	Proasellus racovitzai	whole organism	1	1
Isopoda	Proasellus rectus	whole organism	1	1
Isopoda	Proasellus solanasi	whole organism	1	1
Isopoda	Proasellus spelaeus	whole organism	0	0
Mysida	Neomysis awatschensis	whole organism	0	0
			**Total malacostracan genes**	39

Additional file 8C. Argonaute and Piwi.

Arthropoda

Class (subphylum)	Species	Tissue type	Argonaute	References	Piwi	References
Insecta	Drosophila melanogaster	whole organism	3	ImmunoDB	1	ImmunoDB
Insecta	Anopheles gambiae	whole organism	3	ImmunoDB	1	ImmunoDB
Insecta	Aedes aegypti	whole organism	4	ImmunoDB	7	ImmunoDB
Chilopoda (Myriapoda)	Strigamia maritima	whole organism	2	Palmer et al., 2015	4	proteome
Arachnida (Chelicerata)	Mesobuthus martensii	whole organism	6	Palmer et al., 2015	3	proteome
Arachnida (Chelicerata)	Ixodes scapularis	whole organism	3	Palmer et al., 2015	Multiple	NCBI
Branchiopoda (Crustacea)	Daphnia pulex	whole organism	1	Palmer et al., 2015	Multiple	NCBI

Malacostraca

Order	Species/Datasets	Tissue type	Argonaute	Piwi
Amphipoda	Echinogammarus veneris	NA	1	1
Amphipoda	Gammarus chevreuxi	NA	4	0
Amphipoda	Gammarus pulex	NA	4	0
Amphipoda	Hyalella azteca_1	NA	0	0
Amphipoda	Hyalella azteca_2	NA	0	0
Amphipoda	Hyalella azteca_3	whole organism	0	0
Amphipoda	Melita plumulosa	whole organism	3	0
Amphipoda	Parhyale hawaiensis	whole organism	4	2
Amphipoda	Talitrus saltator	brain	5	0
Decapoda	Astacus astacus	hepatopancreas, ovaries, green glands, abdominal musculature	2	2
Decapoda	Astacus leptodactylus_1	hypodermis; Y organ	3	1
Decapoda	Astacus leptodactylus_2	hepatopancreas	1	1
Decapoda	Astacus leptodactylus_3	hypodermis, Y organ, hepatopancreas, gills, hematocytes, muscle	3	1
Decapoda	Callinectes sapidus	gill 7	2	0
Decapoda	Cancer borealis	nervous system	3	1
Decapoda	Carcinus maenas	NA	4	1
Decapoda	Cherax quadricarinatus_1	hypodermis and gastrolith disc	0	0
Decapoda	Cherax quadricarinatus_2	heart, kidney, liver, nerve, testis	3	2
Decapoda	Cherax quadricarinatus_3	heart, kidney, liver, nerve, testis	3	2
Decapoda	Eriocheir sinensis_1	NA	3	0
Decapoda	Eriocheir sinensis_2	eyestalk, Y-organ, and hepatopancreas	3	0
Decapoda	Eriocheir sinensis_3	hepatopancreas	3	0
Decapoda	Farfantepenaeus aztecus	hepatopancreas	3	1
Decapoda	Homarus americanus	nervous system	2	2
Decapoda	Hyas araneus_1	adult	2	0
Decapoda	Hyas araneus_2	gill	3	0
Decapoda	Litopenaeus vannamei_1	Ghaffari et al., 2014	3	3
Decapoda	Litopenaeus vannamei_2	hepatopancreas	2	1
Decapoda	Litopenaeus vannamei_3	hepatopancreas	3	1
Decapoda	Litopenaeus vannamei_4	hemocytes	1	2
Decapoda	Macrobrachium nipponense	NA	3	0
Decapoda	Pacifastacus leniusculus	Brain, HPT, Hemocyte, Hepatopancreas	3	0
Decapoda	Palaemon argentinus	whole organism	1	0
Decapoda	Penaeus monodon_1	hepatopancreas	5	0
Decapoda	Penaeus monodon_2	hepatopancreas	5	0
Decapoda	Procambarus clarkii_1	Eyestalk	1	1
Decapoda	Procambarus clarkii_2	Eyestalk, brain, hemocytes, gills, testis, ovary, hepatopancreas, heart, green gland, ventralganglia, Y-organ, hypodermis, muscle	4	3
Decapoda	Scylla olivacea	Na	3	3
Decapoda	Scylla paramamosain	gill	0	0
Euphausiacea	Euphausia superba	NA	2	0
Euphausiacea	Meganyctiphanes norvegica	adult	0	1
Isopoda	Asellus aquaticus	NA	0	0
Isopoda	Bragasellus molinai	whole organism	2	0
Isopoda	Bragasellus peltatus	whole organism	3	1
Isopoda	Proasellus aragonensis	whole organism	4	2
Isopoda	Proasellus arthrodilus	whole organism	3	2
Isopoda	Proasellus assaforensis	whole organism	3	2
Isopoda	Proasellus beticus	whole organism	2	0
Isopoda	Proasellus cantabricus	whole organism	2	1
Isopoda	Proasellus cavaticus	whole organism	3	2

Isopoda	Proasellus coiffaiti	whole organism	3	2
Isopoda	Proasellus coxalis	whole organism	3	2
Isopoda	Proasellus ebrensis	whole organism	2	1
Isopoda	Proasellus escolai	whole organism	3	2
Isopoda	Proasellus grafi	whole organism	3	2
Isopoda	Proasellus granadensis	whole organism	2	1
Isopoda	Proasellus hercegovinensis	whole organism	2	2
Isopoda	Proasellus ibericus	whole organism	3	1
Isopoda	Proasellus jaloniacus	whole organism	3	2
Isopoda	Proasellus karamani	whole organism	3	1
Isopoda	Proasellus margalefi	whole organism	1	1
Isopoda	Proasellus meridianus	whole organism	3	3
Isopoda	Proasellus ortizi	whole organism	4	2
Isopoda	Proasellus parvulus	whole organism	2	2
Isopoda	Proasellus racovitzai	whole organism	2	2
Isopoda	Proasellus rectus	whole organism	3	2
Isopoda	Proasellus solanasi	whole organism	3	2
Isopoda	Proasellus spelaeus	whole organism	2	2
Mysida	Neomysis awatschensis	whole organism	1	1

	Argonaute	Piwi
Echinogamm	1	1
Gammarus cl	4	0
Gammarus p	4	0
Hyalella azte	0	0
Melita plumu	3	0
Parhyale haw	4	2
Talitrus salta	5	0
Astacus astac	2	2
Astacus lepto	3	1
Callinectes sa	2	0
Cancer borea	3	1
Carcinus mae	4	1
Cherax quadr	3	2
Eriocheir sine	3	0
Farfantepena	3	1
Homarus am	2	2
Hyas araneus	3	0
Litopenaeus	3	3
Macrobrachi	3	0
Pacifastacus	3	0
Palaemon ar	1	0
Penaeus mor	5	0
Procambarus	4	3
Scylla olivace	3	3
Scylla param	0	0
Euphausia su	2	0
Meganyctiph	0	1
Asellus aquat	0	0
Bragasellus n	2	0
Bragasellus p	3	1
Proasellus ar	4	2
Proasellus ar	3	2
Proasellus as	3	2
Proasellus be	2	0
Proasellus ca	2	1
Proasellus ca	3	2
Proasellus co	3	2
Proasellus co	3	2
Proasellus eb	2	1
Proasellus es	3	2
Proasellus gr	3	2
Proasellus gr	2	1
Proasellus he	2	2
Proasellus ib	3	1
Proasellus jal	3	2
Proasellus ka	3	1
Proasellus m	1	1
Proasellus m	3	3
Proasellus or	4	2
Proasellus pa	2	2
Proasellus ra	2	2

Proasellus re	3	2
Proasellus so	3	2
Proasellus sp	2	2
Neomysis aw	1	1
**Total malaco**	143	66

Additional file 8D. TRBP (TAR RNA binding protein).

Arthropoda

Class (subphylum)	Species	Tissue type	Total gene counts	References
Insecta	Drosophila melanogaster	whole organism	1 Loquacious	ImmunoDB
Insecta	Anopheles gambiae	whole organism	1	ImmunoDB
Insecta	Aedes aegypti	whole organism	1	ImmunoDB
Chilopoda (Myriapoda)	Strigamia maritima	whole organism	1	proteome
Arachnida (Chelicerata)	Mesobuthus martensii	whole organism	1	proteome
Arachnida (Chelicerata)	Ixodes scapularis	whole organism	1	uniprot
Branchiopoda (Crustacea)	Daphnia pulex	whole organism	1	proteome

Malacostraca

Order	Species/Datasets	Tissue type	Total gene counts	Total number of non-redundant genes per species
Amphipoda	Echinogammarus veneris	NA	1	1
Amphipoda	Gammarus chevreuxi	NA	1	1
Amphipoda	Gammarus pulex	NA	1	1
Amphipoda	Hyalella azteca_1	NA	1	
Amphipoda	Hyalella azteca_2	NA	0	
Amphipoda	Hyalella azteca_3	whole organism	0	1
Amphipoda	Melita plumulosa	whole organism	1	1
Amphipoda	Parhyale hawaiensis	whole organism	1	1
Amphipoda	Talitrus saltator	brain	1	1
Decapoda	Astacus astacus	hepatopancreas, ovaries, green glands, abdominal musculature	1	1
Decapoda	Astacus leptodactylus_1	hypodermis; Y organ	1	
Decapoda	Astacus leptodactylus_2	hepatopancreas	1	
Decapoda	Astacus leptodactylus_3	hypodermis, Y organ, hepatopancreas, gills, hematocytes, muscle	1	1
Decapoda	Callinectes sapidus	gill 7	1	1
Decapoda	Cancer borealis	nervous system	1	1
Decapoda	Carcinus maenas	NA	1	1
Decapoda	Cherax quadricarinatus_1	hypodermis and gastrolith disc	1	
Decapoda	Cherax quadricarinatus_2	heart, kidney, liver, nerve, testis	1	
Decapoda	Cherax quadricarinatus_3	heart, kidney, liver, nerve, testis	1	1
Decapoda	Eriocheir sinensis_1	NA	1	
Decapoda	Eriocheir sinensis_2	eyestalk, Y-organ, and hepatopancreas	1	
Decapoda	Eriocheir sinensis_3	hepatopancreas	1	1
Decapoda	Farfantepenaeus aztecus	hepatopancreas	1	1
Decapoda	Homarus americanus	nervous system	1	1
Decapoda	Hyas araneus_1	adult	0	
Decapoda	Hyas araneus_2	gill	1	1
Decapoda	Litopenaeus vannamei_1	Ghaffari et al., 2014	1	
Decapoda	Litopenaeus vannamei_2	hepatopancreas	1	
Decapoda	Litopenaeus vannamei_3	hepatopancreas	1	
Decapoda	Litopenaeus vannamei_4	hemocytes	1	1
Decapoda	Macrobrachium nipponense	NA	1	1
Decapoda	Pacifastacus leniusculus	Brain, HPT, Hemocyte, Hepatopancreas	1	1
Decapoda	Palaemon argentinus	whole organism	1	1
Decapoda	Penaeus monodon_1	hepatopancreas	1	
Decapoda	Penaeus monodon_2	hepatopancreas	1	1
Decapoda	Procambarus clarkii_1	Eyestalk	1	
Decapoda	Procambarus clarkii_2	Eyestalk, brain, hemocytes, gills, testis, ovary, hepatopancreas, heart, green gland, ventralganglia, Y-organ, hypodermis, muscle	1	1
Decapoda	Scylla olivacea	Na	1	1
Decapoda	Scylla paramamosain	gill	0	0
Euphausiacea	Euphausia superba	NA	1	1
Euphausiacea	Meganyctiphanes norvegica	adult	0	0
Isopoda	Asellus aquaticus	NA	1	1
Isopoda	Bragasellus molinai	whole organism	1	1
Isopoda	Bragasellus peltatus	whole organism	1	1
Isopoda	Proasellus aragonensis	whole organism	1	1
Isopoda	Proasellus arthrodilus	whole organism	1	1
Isopoda	Proasellus assaforensis	whole organism	1	1
Isopoda	Proasellus beticus	whole organism	0	0
Isopoda	Proasellus cantabricus	whole organism	1	1
Isopoda	Proasellus cavaticus	whole organism	1	1
Isopoda	Proasellus coiffaiti	whole organism	1	1
Isopoda	Proasellus coxalis	whole organism	1	1
Isopoda	Proasellus ebrensis	whole organism	1	1

Isopoda	Proasellus escolai	whole organism	1	1
Isopoda	Proasellus grafi	whole organism	1	1
Isopoda	Proasellus granadensis	whole organism	1	1
Isopoda	Proasellus hercegovinensis	whole organism	1	1
Isopoda	Proasellus ibericus	whole organism	1	1
Isopoda	Proasellus jaloniacus	whole organism	1	1
Isopoda	Proasellus karamani	whole organism	1	1
Isopoda	Proasellus margalefi	whole organism	0	0
Isopoda	Proasellus meridianus	whole organism	1	1
Isopoda	Proasellus ortizi	whole organism	1	1
Isopoda	Proasellus parvulus	whole organism	0	0
Isopoda	Proasellus racovitzai	whole organism	1	1
Isopoda	Proasellus rectus	whole organism	1	1
Isopoda	Proasellus solanasi	whole organism	1	1
Isopoda	Proasellus spelaeus	whole organism	1	1
Mysida	Neomysis awatschensis	whole organism	1	1
			**Total malacostracan genes**	50

Additional file 8E. Pasha.

Arthropoda

Class (subphylum)	Species	Tissue type	Total gene counts	References
Insecta	Drosophila melanogaster	whole organism	1	ImmunoDB
Insecta	Anopheles gambiae	whole organism	1	ImmunoDB
Insecta	Aedes aegypti	whole organism	1	ImmunoDB
Chilopoda (Myriapoda)	Strigamia maritima	whole organism	1	proteome
Arachnida (Chelicerata)	Mesobuthus martensii	whole organism	2	proteome
Arachnida (Chelicerata)	Ixodes scapularis	whole organism	1	proteome
Branchiopoda (Crustacea)	Daphnia pulex	whole organism	1	proteome

Malacostraca

Order	Species/Datasets	Tissue type	Total gene counts	Total number of non-redundant genes per species
Amphipoda	Echinogammarus veneris	NA	0	0
Amphipoda	Gammarus chevreuxi	NA	1	1
Amphipoda	Gammarus pulex	NA	0	0
Amphipoda	Hyalella azteca_1	NA	1	
Amphipoda	Hyalella azteca_2	NA	0	
Amphipoda	Hyalella azteca_3	whole organism	1	1
Amphipoda	Melita plumulosa	whole organism	0	0
Amphipoda	Parhyale hawaiensis	whole organism	1	1
Amphipoda	Talitrus saltator	brain	1	1
Decapoda	Astacus astacus	hepatopancreas, ovaries, green glands, abdominal musculature	1	1
Decapoda	Astacus leptodactylus_1	hypodermis; Y organ	1	
Decapoda	Astacus leptodactylus_2	hepatopancreas	0	
Decapoda	Astacus leptodactylus_3	hypodermis, Y organ, hepatopancreas, gills, hematocytes, muscle	1	1
Decapoda	Callinectes sapidus	gill 7	1	1
Decapoda	Cancer borealis	nervous system	1	1
Decapoda	Carcinus maenas	NA	1	1
Decapoda	Cherax quadricarinatus_1	hypodermis and gastrolith disc	0	
Decapoda	Cherax quadricarinatus_2	heart, kidney, liver, nerve, testis	1	
Decapoda	Cherax quadricarinatus_3	heart, kidney, liver, nerve, testis	0	1
Decapoda	Eriocheir sinensis_1	NA	1	
Decapoda	Eriocheir sinensis_2	eyestalk, Y-organ, and hepatopancreas	0	
Decapoda	Eriocheir sinensis_3	hepatopancreas	0	1
Decapoda	Farfantepenaeus aztecus	hepatopancreas	1	1
Decapoda	Homarus americanus	nervous system	1	1
Decapoda	Hyas araneus_1	adult	0	
Decapoda	Hyas araneus_2	gill	1	1
Decapoda	Litopenaeus vannamei_1	Ghaffari et al., 2014	1	
Decapoda	Litopenaeus vannamei_2	hepatopancreas	1	
Decapoda	Litopenaeus vannamei_3	hepatopancreas	1	
Decapoda	Litopenaeus vannamei_4	hemocytes	1	1
Decapoda	Macrobrachium nipponense	NA	0	0
Decapoda	Pacifastacus leniusculus	Brain, HPT, Hemocyte, Hepatopancreas	0	0
Decapoda	Palaemon argentinus	whole organism	0	0
Decapoda	Penaeus monodon_1	hepatopancreas	1	
Decapoda	Penaeus monodon_2	hepatopancreas	1	1
Decapoda	Procambarus clarkii_1	Eyestalk	0	
Decapoda	Procambarus clarkii_2	Eyestalk, brain, hemocytes, gills, testis, ovary, hepatopancreas, heart, green gland, ventralganglia, Y-organ, hypodermis, muscle	1	1
Decapoda	Scylla olivacea	Na	1	1
Decapoda	Scylla paramamosain	gill	1	1
Euphausiacea	Euphausia superba	NA	1	1
Euphausiacea	Meganyctiphanes norvegica	adult	0	0
Isopoda	Asellus aquaticus	NA	0	0
Isopoda	Bragasellus molinai	whole organism	1	1
Isopoda	Bragasellus peltatus	whole organism	1	1
Isopoda	Proasellus aragonensis	whole organism	1	1
Isopoda	Proasellus arthrodilus	whole organism	1	1
Isopoda	Proasellus assaforensis	whole organism	1	1
Isopoda	Proasellus beticus	whole organism	0	0
Isopoda	Proasellus cantabricus	whole organism	1	1
Isopoda	Proasellus cavaticus	whole organism	1	1
Isopoda	Proasellus coiffaiti	whole organism	1	1
Isopoda	Proasellus coxalis	whole organism	1	1
Isopoda	Proasellus ebrensis	whole organism	1	1

Isopoda	Proasellus escolai	whole organism	1	1
Isopoda	Proasellus grafi	whole organism	1	1
Isopoda	Proasellus granadensis	whole organism	1	1
Isopoda	Proasellus hercegovinensis	whole organism	1	1
Isopoda	Proasellus ibericus	whole organism	1	1
Isopoda	Proasellus jaloniacus	whole organism	1	1
Isopoda	Proasellus karamani	whole organism	1	1
Isopoda	Proasellus margalefi	whole organism	1	1
Isopoda	Proasellus meridianus	whole organism	1	1
Isopoda	Proasellus ortizi	whole organism	1	1
Isopoda	Proasellus parvulus	whole organism	1	1
Isopoda	Proasellus racovitzai	whole organism	1	1
Isopoda	Proasellus rectus	whole organism	1	1
Isopoda	Proasellus solanasi	whole organism	1	1
Isopoda	Proasellus spelaeus	whole organism	1	1
Mysida	Neomysis awatschensis	whole organism	1	1
			**Total gene counts**	46
