# Supplementary material for: Comparative genomic analysis of innate immunity reveals novel and conserved components in crustacean food crop species
Source: BMC Genomics. 2017 May 18;18:389. doi: 10.1186/s12864-017-3769-4 (PMC5437397; doi:10.1186/s12864-017-3769-4)
Supplement: Supplementary file 19 — Primer sequences used for RT-PCR of novel malacostracan genes in Parhyale hawaiensis. (PDF 33 kb) [file 12864_2017_3769_MOESM19_ESM.pdf]

Additional file 10. Primer sequences used for RT-PCR of novel malacostracan genes in Parhyale hawaiensis.

Parhyale hawaiensis transcript IDs	Pfam domain	Forward primers	5'-3'	Reverse primers	5'3'	Length (bp)
phaw_30_tra_m.000427	Death	Mala_1F	CATACATTACAGCTTCGAGTGCAG	Mala_1R	TTTGTTTGTTGCTAAGCGAGTGAT	508
phaw_30_tra_m.014321	ML	Mala_2F	TCGACCAGTTCAGTATTCGTTCAG	Mala_2R	AACTCAGTCATCATCATACCGTGG	563
phaw_30_tra_m.013935	Perithropin	Mala_3F	TACCGAGTACGACCACTGAATTTT	Mala_3R	AGTTACGAGCAATGGTGAAGTAGT	500
phaw_30_tra_m.003904	vWFA	Mala_4F	CATGAGTAGCGTCTGGAGACTC	Mala_4R	CTCAACAGGGAAAGACCGACC	355
phaw_30_tra_m.006435	vWFA	Mala_5F	TTTGCCGTTAGTTCCAGAGACTAA	Mala_5R	CGTGTCCTTTGATTTTGAGAAGCT	509
phaw_30_tra_m.015532	vWFA	Mala_6F	CTGGGAAGATTGTACACCACAGTA	Mala_6R	TAGAACTTTTCCTGGAATGGAGCA	611
phaw_30_tra_m.023789	vWFA	Mala_7F	TAGAGAAGGACGATTTTGACTGCA	Mala_7R	ATCATCGACAACTTTCAGCGTTTT	503
phaw_30_tra_m.025676	vWFA	Mala_8F	AATGGTTGATGTGTCAGGCTCTAT	Mala_8R	AGCAAGTACACTTCGATGGTTTTG	348
