# Supplementary material for: Comparative genomic analysis of innate immunity reveals novel and conserved components in crustacean food crop species
Source: BMC Genomics. 2017 May 18;18:389. doi: 10.1186/s12864-017-3769-4 (PMC5437397; doi:10.1186/s12864-017-3769-4)
Supplement: Supplementary file 26 — Supplementary references (DOCX 31 kb) [file 12864_2017_3769_MOESM24_ESM.docx]

**Supplementary references (for transcriptome datasets used in this study)**

Christie AE. 2014. Identification of the first neuropeptides from the Amphipoda (Arthropoda, Crustacea). Gen Comp Endocrinol. 206:96–110.

Gismondi E, Thomé JP. 2016. Transcriptome of the freshwater amphipod *Gammarus pulex* hepatopancreas. Genomics Data 8:91–92.

Glazer L, Tom M, Weil S, Roth Z, Khalaila I, Mittelman B, Sagi A. 2013. Hemocyanin with phenoloxidase activity in the chitin matrix of the crayfish gastrolith. J Exp Biol. 216:1898–1904.

Harms L, Frickenhaus S, Schiffer M, Mark FC, Storch D, Pörtner H-O, Held C, Lucassen M. 2013. Characterization and analysis of a transcriptome from the boreal spider crab *Hyas* *araneus*. Comp Biochem Physiol Part D Genomics Proteomics 8:344–351.

Hook SE, Twine NA, Simpson SL, Spadaro DA, Moncuquet P, Wilkins MR. 2014. 454 pyrosequencing-based analysis of gene expression profiles in the amphipod *Melita* *plumulosa*: Transcriptome assembly and toxicant induced changes. Aquat Toxicol. 153:73–88.

Johnson JG, Burnett LE, Burnett KG. 2016. Uncovering Hemocyanin Subunit Heterogeneity in Penaeid Shrimp using RNA-Seq. Integr Comp Biol.:icw088–12.

Johnson JG, Paul MR, Kniffin CD, Anderson PE, Burnett LE, Burnett KG. 2015. High CO2 alters the hypoxia response of the Pacific whiteleg shrimp (*Litopenaeus vannamei*) transcriptome including known and novel hemocyanin isoforms. Physiol Genomics 47:548–558.

Manfrin C, Tom M, De Moro G, Gerdol M, Giulianini PG, Pallavicini A. 2015. The eyestalk transcriptome of red swamp crayfish *Procambarus clarkii*. Gene 557:28–34.

Manfrin C, Tom M, De Moro G, Gerdol M, Guarnaccia C, Mosco A, Pallavicini A, Giulianini PG. 2013. Application of D-crustacean hyperglycemic hormone induces peptidases transcription and suppresses glycolysis-related transcripts in the hepatopancreas of the crayfish *Pontastacus leptodactylus* — Results of a Transcriptomic Study. PLoS ONE 8:e65176–10.

Northcutt AJ, Lett KM, Garcia VB, Diester CM, Lane BJ, Marder E, Schulz DJ. 2016. Deep sequencing of transcriptomes from the nervous systems of two decapod crustaceans to characterize genes important for neural circuit function and modulation. BMC Genomics 17:1–22.

O’Grady JF, Hoelters LS, Swain MT, Wilcockson DC. 2016. Identification and temporal expression of putative circadian clock transcripts in the amphipod crustacean *Talitrus saltator*. PeerJ 4:e2555–27.

Theissinger K, Falckenhayn C, Blande D, Toljamo A, Gutekunst J, Makkonen J, Jussila J, Lyko F, Schrimpf A, Schulz R, et al. 2016. De Novo assembly and annotation of the freshwater crayfish *Astacus astacus* transcriptome. Mar Genom. 28:7–10.

Tom M, Manfrin C, Chung SJ, Sagi A, Gerdol M, De Moro G, Pallavicini A, Giulianini PG. 2014. Expression of cytoskeletal and molt-related genes is temporally scheduled in the hypodermis of the crayfish *Procambarus clarkii* during premolt. J Exp Biol. 217:4193–4202.

Tom M, Manfrin C, Giulianini PG, Pallavicini A. 2013. Crustacean oxi-reductases protein sequences derived from a functional genomic project potentially involved in ecdysteroid hormones metabolism – A starting point for function examination. Gen Comp Endocrinol. 194:71–80.

Weston DP, Poynton HC, Wellborn GA. 2013. Multiple origins of pyrethroid insecticide resistance across the species complex of a nontarget aquatic crustacean, *Hyalella azteca*. Proc Natl Acad Sci U.S.A. 110:16532-16537.

Xu Y, Li X, Deng Y, Lu Q, Yang Y, Pan J, Ge J, Xu Z. 2016. Comparative transcriptome sequencing of the hepatopancreas reveals differentially expressed genes in the precocious juvenile Chinese mitten crab, *Eriocheir sinensis*(Crustacea: Decapoda). Aquac Res. 1–12.

Xu Z, Zhao M, Li X, Lu Q, Li Y, Ge J, Pan J. 2015. Transcriptome profiling of the eyestalk of precocious juvenile Chinese mitten crab reveals putative neuropeptides and differentially expressed genes. Gene 569:280–286.

Xue S, Liu Y, Zhang Y, Sun Y, Geng X, Sun J. 2013. Sequencing and de novo analysis of the hemocytes transcriptome in *Litopenaeus vannamei* response to White Spot Syndrome Virus infection. PLoS ONE 8:e76718–12.
