# Supplementary material for: Comparative genomic analysis of innate immunity reveals novel and conserved components in crustacean food crop species
Source: BMC Genomics. 2017 May 18;18:389. doi: 10.1186/s12864-017-3769-4 (PMC5437397; doi:10.1186/s12864-017-3769-4)
Supplement: Supplementary file 14 — Suppressor of cytokine signalling (SOCS) gene family in malacostracans. Phylogenetic tree of the SOCS gene family is constructed using the maximum-likelihood method from an amino acid multiple sequence alignment. Seven main groups of SOCS proteins are identified in malacostracans. Taxa labels are depicted as their respective colour codes. Bootstrap support values (n=1000) for all trees can be found in Additional file 14: Figure S8. Scale bar represents substitution per site. (PDF 1157 kb) [file 12864_2017_3769_MOESM14_ESM.pdf]

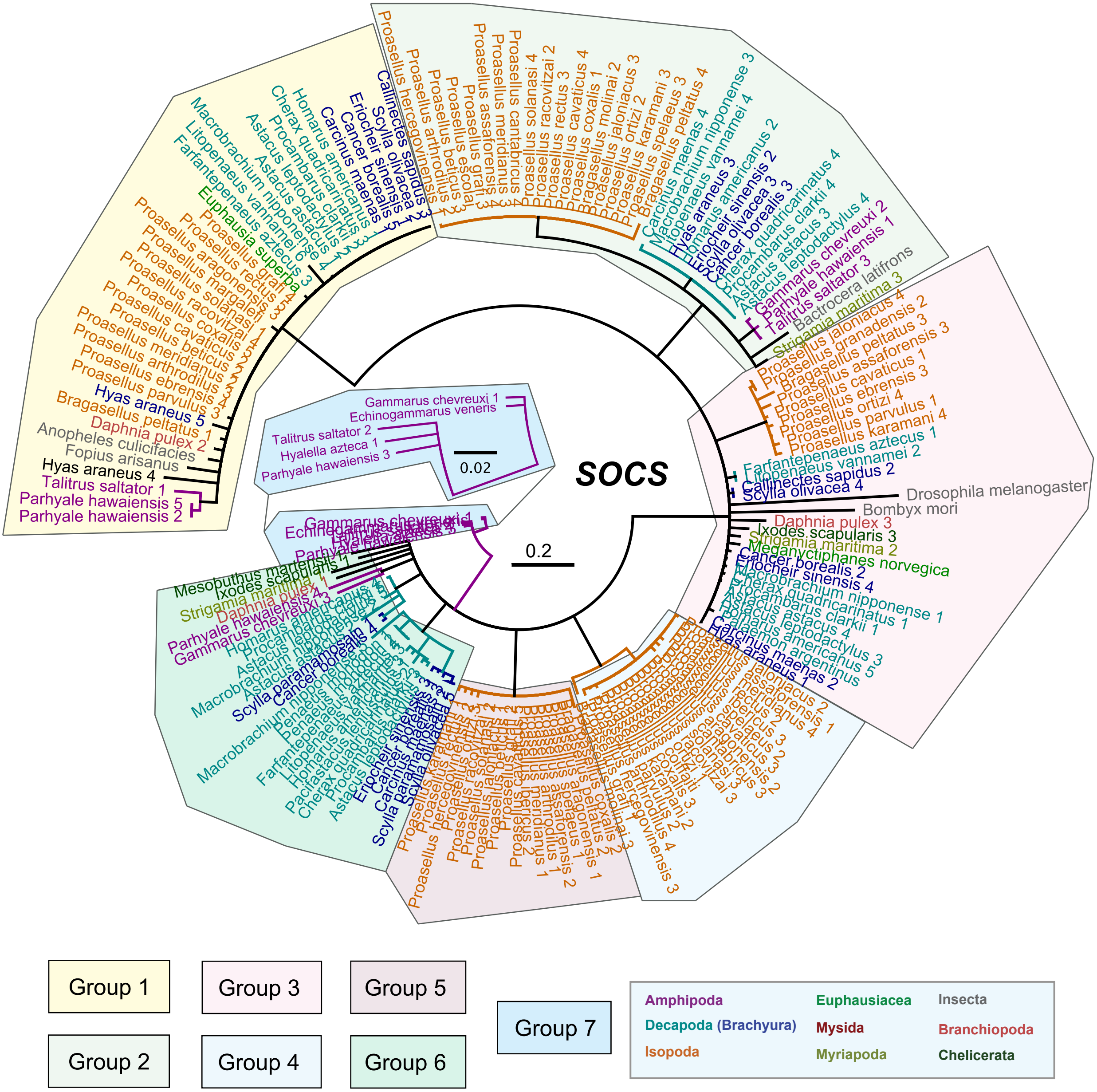

SOCS
0.2
0.02
Group 1
Group 2
Group 3
Group 4
Group 5
Group 6
Group 7
Amphipoda
Decapoda (Brachyura)
Isopoda
Euphausiacea
Mysida
Myriapoda
Insecta
Branchiopoda
Chelicerata
