# Supplementary material for: Comparative genomic analysis of innate immunity reveals novel and conserved components in crustacean food crop species
Source: BMC Genomics. 2017 May 18;18:389. doi: 10.1186/s12864-017-3769-4 (PMC5437397; doi:10.1186/s12864-017-3769-4)
Supplement: Supplementary file 23 — Novel gene family with potential innate immunity function characterised by the Von Willebrand factor type A domain (pfam05762) found only in malacostracans. (A) Phylogenetic tree of proteins containing the Von Willebrand factor type A domain is constructed using the maximum-likelihood method from an amino acid multiple sequence alignment. Taxa labels are depicted as their respective colour codes. Node labels represent bootstrap support values from 1000 replicates. Scale bar represents substitution per site. (B) Multiple sequence alignment of the Von Willebrand factor type A domain from this gene family. (PDF 5243 kb) [file 12864_2017_3769_MOESM23_ESM.pdf]

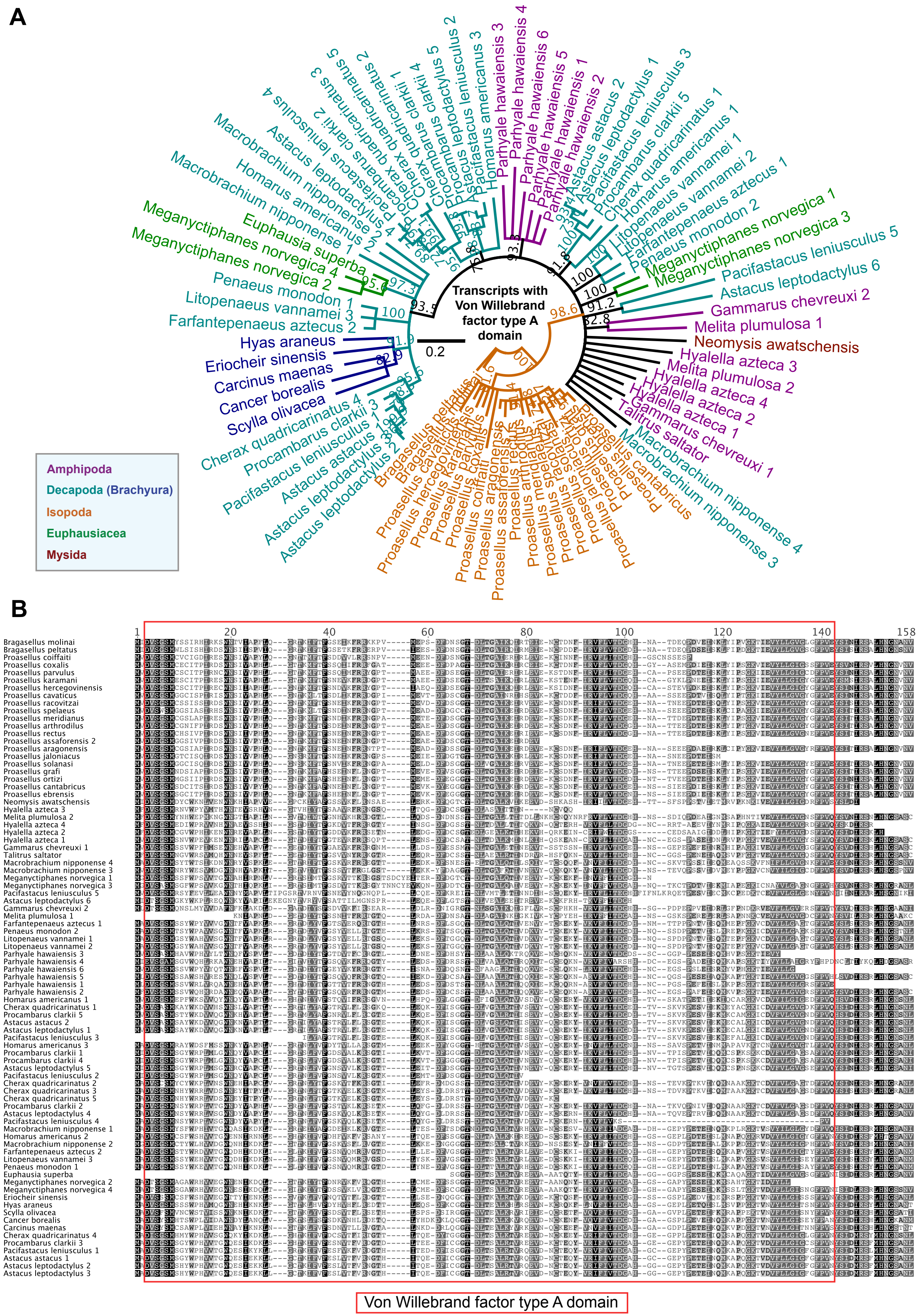
A
Transcripts with Von Willebrand factor type A domain
0.2
Amphipoda
Decapoda (Brachyura)
Isopoda
Euphausiacea
Mysida
B
Von Willebrand factor type A domain
